# Prevalence of and risk factors for surgical site infections after pancreaticoduodenectomy: a systematic review and meta-analysis

**DOI:** 10.1097/MS9.0000000000001455

**Published:** 2023-11-07

**Authors:** Hongfei Hu, Ting Zhou, Yijin Qiu, Yuxin Li, Wei Liu, Rui Meng, Xueke Zhang, Aixia Ma, Hongchao Li

**Affiliations:** a School of International Pharmaceutical Business; b Center for Pharmacoeconomics and Outcomes Research, China Pharmaceutical University, Nanjing, Jiangsu, People’s Republic of China

**Keywords:** pancreaticoduodenectomy, prevalence, risk factor, surgical site infections

## Abstract

**Background:**

Surgical site infections (SSIs) are one of the most common complications after pancreaticoduodenectomy (PD); however, the global prevalence and risk factors for SSIs after PD remain unknown.

**Objectives:**

To investigate the prevalence of and risk factors for SSIs after PD.

**Methods:**

The PubMed, Embase, Cochrane Library, Web of Science, and Science Direct databases were systematically searched from inception to 1 December 2022. Observational studies reporting adjusted odds ratios (ORs) and 95% confidence intervals (CIs) of risk factors for SSIs in patients undergoing PD were included. Two independent reviewers in teams performed data extraction, risk of bias assessment, and level of evidence analysis. The pooled results were estimated using a random-effects model. The *I* ^2^ statistic and Q *χ* ^2^ statistic were used to assess heterogeneity. Funnel plots, Egger’s regression test, and the trim-and-fill method were used to determine publication bias. The primary outcomes were identifying risk factors for SSIs after PD. The secondary outcomes were the pooled prevalence rates of SSIs.

**Results:**

A total of 98 704 patients from 45 studies were included, and 80% of the studies were considered high quality. The estimated pooled prevalence of SSIs was 23% (0.19–0.27, *I* ^2^=97%). The prevalence of SSIs was found to be higher in Japan and lower in USA. Preoperative biliary stenting, higher body mass index (BMI), longer operation time, postoperative pancreatic fistula, soft pancreatic texture, perioperative blood transfusion, and cardiac disease were identified as significant risk factors for the development of SSIs after PD. Additionally, broad-spectrum antibiotics were a significant protective factor against SSIs. Subgroup analysis and sensitivity analysis showed that the results were robust.

**Conclusion and relevance:**

The prevalence of SSIs remains high and varies widely among regions. It is necessary to take effective preventive measures and carry out more prospective studies to further verify these results.

## Introduction

Highlights

- This is the first systematic review and meta-analysis of multiple risk factors for surgical site infections (SSIs) after pancreaticoduodenectomy (PD).
- Nearly 23% of patients after PD had SSIs. And the prevalence of SSIs remains high and varies widely among regions.
- Preoperative biliary stenting, higher body mass index, longer operation time, postoperative pancreatic fistula, soft pancreatic texture, perioperative blood transfusion, and cardiac disease were significant risk factors for SSIs.
- Further studies are needed to elucidate the differences among regions and to determine the potential risk of SSIs.

Pancreaticoduodenectomy (PD) is a complicated medical surgical procedure used to treat diseases that are found in the periampullary region. It is one of the most challenging surgical procedures due to the complexity of the region being operated on and the high prevalence of postoperative complications^1,2^. Nearly one-third of patients have experienced complications after PD, among which surgical site infections (SSIs) are one of the most common complications, with a prevalence of up to 23.5%^1,3^.

According to the US Centers for Disease Control and Prevention (CDC) criteria, SSIs include superficial incisional SSIs, deep incisional SSIs, and organ/space SSIs^4^. A correlation has been observed between SSIs and various adverse outcomes, such as heightened postoperative pain and psychosocial distress, impaired wound healing, prolonged hospitalization, frequent readmissions, and elevated healthcare expenses^5–7^.

Therefore, it is necessary to implement measures to safeguard patients at a high risk of developing SSIs and to prevent potentially severe outcomes. Multiple factors, such as preoperative biliary stenting^8,9^, antibiotic prophylaxis^10^, and wound protector use^11^, have been reported to be related to SSIs. However, this evidence is scattered and difficult to identify. In addition, most of the studies were focused on one specific factor^8–11^. The main objective of this study was to systematically explore risk factors for SSIs after PD. We also explored and estimated the prevalence of SSIs based on the literature included in the analysis of risk factors.

## Methods

This study was performed in accordance with the Preferred Reporting Items for Systematic Review and Meta-analysis (PRISMA)^12^ reporting guidelines (Supplemental Digital Content 1, http://links.lww.com/MS9/A298; Supplemental Digital Content 4, http://links.lww.com/MS9/A301) and A MeaSurement Tool to Assess systematic Reviews (AMSTAR) 2^13^ (Supplemental Digital Content 2, http://links.lww.com/MS9/A299). This review was registered in the Research Registry under the UIN: reviewregistry1669 (www.researchregistry.com).

### Search strategy

Search strategies were developed by a senior health sciences investigator. A pilot search was performed via PubMed to determine the search strategy. Then, we systematically searched the PubMed, Embase, Cochrane Library, Web of Science, and Science Direct databases up to 1 December 2022 to identify relevant articles. The search strategy was adjusted based on the syntax and subject headings of each database. The retrieval strategy adopted a combination of subject heading terms and keywords. The details of the search strategy for each database are presented in the Supplemental Digital Content 3, Table 1 (http://links.lww.com/MS9/A300).

### Inclusion/exclusion criteria

Studies were included if they met the following criteria: (1) patients who underwent PD; (2) evaluated the associated factors of SSIs; (3) reported adjusted odds ratios (ORs) and 95% confidence intervals (CIs) for SSIs; (4) observational study designs (cohort study, case–control study, and cross-sectional study); (5) published in English language; and (6) full text available. Except for not meeting the inclusion criteria, the exclusion criteria were as follows: (1) failure to determine the outcome of interest and (2) conference abstracts, study protocols, case reports, reviews, letters, and animal studies.

### Study selection

All research literature retrieved was imported into EndNote X9.1. The study selection process included initial screening, secondary screening, and a final check. After removing duplicates, two reviewers screened titles and abstracts to exclude unrelated studies and conference abstracts according to predefined selection criteria. The eligible studies were included through full-text screening according to the criteria. If a conference abstract met our inclusion criteria, we proceeded to search for the corresponding full-text articles. In cases where full-text articles were not available, we contacted the principal investigators to verify whether the studies were published and to acquire data. Detailed discussion took place if there were any disagreements between the two reviewers. A third investigator would join and make the decision if the agreement could not be reached.

### Risk of bias assessment

Quality assessment of the included studies was independently assessed by two reviewers using the Newcastle–Ottawa scale (NOS)^14^, a revised Cochrane risk-of-bias tool for observational studies that include the following domains: the selection of the study groups (0–4 points), the comparability of the groups (0–2 points), and the determination of either the exposure for cohort study or the outcome of interest for case–control study (0–3 points). The maximum score is 9 points. A score greater than 6 points was considered to indicate high quality. A third researcher was consulted to resolve the disagreement in case of any discrepancy.

### Data extraction

Two reviewers collected data independently from the eligible studies and recorded them in predesigned study information tables in Microsoft Excel 2019. The following data were extracted and recorded: first author, publication year, region, study period, type of surgery, the definition of SSIs, data source, sample size, study design, type of SSIs, the prevalence of SSIs, risk factors, and adjusted ORs with 95% CIs. Any disagreement was settled by discussion.

SSIs were categorized into different groups: total SSIs, superficial incisional SSIs, deep incisional SSIs, organ/space incisional SSIs, and superficial/deep incisional SSIs according to the US CDC criteria and study description. Total SSIs indicated that SSI types were not distinguished in the included study. Superficial incisional SSIs, deep incisional SSIs, and organ/space incisional SSIs are consistent with the US CDC criteria.

### Statistical analysis

To obtain the pooled prevalence of SSI and ORs with 95% CIs, a meta-analysis using the random-effects model of DerSimonian and Laird^15^ was performed, given the unavoidable heterogeneity among the included studies. In addition, the results of the common-effects model (namely the fixed-effects model) are also presented in the figures and tables. A normality test was performed on the prevalence, which was transformed by four estimation methods, including logarithmic, logit, arcsine, and Freeman–Tukey double arcsine transformations. The normality of the untransformed prevalence was also assessed before the meta-analysis. Then, we selected the results that were close to the normal distribution. ORs and corresponding 95% CIs were also log transformed before analysis.

Heterogeneity was tested using the Cochrane’s Q *χ* ^2^ statistics (significance level at *P*<0.10) and the *I* ^2^ statistic (significance level at *I* ^2^ >50%)^16^. In case of heterogeneity, subgroup analysis was carried out based on the characteristics of studies, including region, study design, and type of SSIs. Sensitivity analysis was performed by excluding one study at a time to test the robustness of the results. In order to analyze the prevalence of and risk factors for SSIs in specific countries, two countries with a substantial number of studies can be selected.

Moreover, potential publication bias was assessed by examining the symmetry of the funnel plot and Egger’s regression test (significance level at *P*<0.10)^17^. The trim-and-fill method was used to determine the impact of publication bias and determine whether the results were robust^18^.

Descriptive analysis was performed by using Microsoft Excel to provide summative figures. Meta-analysis was performed using the software R 4.2.2. The threshold for statistically significant differences was a two-tailed *P* value less than 0.05, except for the tests of heterogeneity and publication bias (*P*<0.10).

## Results

### Search results

In the initial search, 875 studies were identified. Of these, after excluding duplicated studies and other unrelated articles, 45 studies were eligible and ultimately included in the meta-analysis^19–63^. The details of the screening process are shown in Supplemental Digital Content 3, Figure 1 (http://links.lww.com/MS9/A300).

### Baseline characteristics of the included studies

The characteristics of the included studies are summarized in Table 1. A total of 45 studies involving 98 704 patients were included in the analysis^19–63^. The number of participants in each study ranged from 51 to 11 562. All included studies were published between 2001 and 2022, with the highest number of publications in 2021^27,36–38,44,46,58,61–63^. More than half of the studies were conducted in the USA (*n*=24)^19,22–26,28–30,35–37,41,43–46,49–51,57,58,62,63^, followed by Japan (*n*=11)^32,33,39,40,42,48,52–55,59^ and China (*n*=4)^27,38,60,61^. Twenty (44.4%) studies^21,24,25,28,31,33,34,39,40,42,43,47,48,51–53,55,56,61,62^ defined SSIs according to the CDC criteria but did not report the detailed definitions of SSIs. Most studies (*n*=26)^20,21,24,25,27,28,31–34,38–40,42,43,47,51–56,59–62^ were conducted at hospitals, while the others used database information. Of these studies, the American College of Surgeons National Surgical Quality Improvement Program (ACS-NSQIP) database was the most commonly used^19,22,23,26,29,30,36,37,41,44–46,49,57,58,63^. For study design, 32 (71.1%) articles^19,22–24,26–31,35–37,40–42,44,46,47,49–52,54,55,57–63^ were retrospective cohort studies, 8 (17.7%)^21,25,32,33,38,45,48,56^ were case–control studies, and 5 (11.1%)^20,34,39,43,53^ were prospective cohort studies. Most studies (*n*=31)^20,21,23,24,26–29,31–34,37–40,43,44,46,47,50–57,59,61,63^ did not report the type of surgery. In terms of risk bias assessment, 36 studies (80.0%)^19–32,34–41,43,44,47,49–53,56,58–61,63^ had high quality, while 9 studies (20.0%)^33,42,45,46,48,54,55,57,62^ had low quality (the details are shown in Supplemental Digital Content 3, Table 2, http://links.lww.com/MS9/A300).

**Table 1:** Baseline characteristics of the included studies

Study	Public year	Region	Study period	Type of surgery	Definition of SSIs	Data source	Sample size	Study design	Type of SSIs	Prevalence (%)	Risk factors^a^	Quality score
Addison *et al*.^19^	2019	USA	2015–2016	MIS vs. Open vs Convert to Open	NR	ACS-NSQIP database	7583	Retrospective cohort study	Organ/space SSIs	15.1	1;2	8
Akerberg *et al*.^20^	2019	Sweden	2010–2016	NR	NR	A surgical unit in Sweden	1036	Prospective cohort study	Total SSIs	8.1	3	8
Barreto *et al*.^21^	2015	India	2010–2014	NR	CDC criteria	A tertiary care referral center, India	277	Case–control study	Superficial/deep incisional SSIs	12.6	4;5;6;17	7
Beane *et al*.^22^	2017	USA	2014–2015	MIS vs. Open vs Convert to Open	ACS-NSQIP	ACS-NSQIP database	6244	Retrospective cohort study	Superficial incisional SSIs	8.7	30	8
									Deep incisional SSIs	2.3	NR	
									Organ/space SSIs	13.6	30	
Bergquist *et al*.^23^	2016	USA	2005–2012	NR	NR	ACS-NSQIP database	923	Retrospective cohort study	Total SSIs	21.5	7	8
									Superficial incisional SSIs	9.8	NR	
									Deep incisional SSIs	2.2	NR	
									Organ/space SSIs	10.8	7	
Bilgic *et al*.^24^	2020	Turkey; USA	2010–2018	NR	CDC criteria	American Hospital & Koc University Hospital	214	Retrospective cohort study	Total SSIs	24.3	4;8–13	7
Burkhart *et al*.^25^	2017	USA	2014–2016	Open	CDC criteria	Johns Hopkins Hospital, USA	394	Case–control study	Total SSIs	19.8	14–17;33	7
Chang *et al*.^26^	2020	USA	2010–2015	NR	NR	ACS-NSQIP database	3484	Retrospective cohort study	Superficial incisional SSIs	8.8	12	7
									Deep incisional SSIs	2.4	12	
									Organ/space SSIs	9.3	12	
Chen *et al*.^27^	2021	China	2012–2018	NR	NHSN criteria	First Affiliated Hospital of Nanjing Medical University, China	1365	Retrospective cohort study	Organ/space SSIs	10.0	18–22	8
Donald *et al*.^28^	2013	USA	2008–2009	NR	CDC criteria	Hospital epidemiology and the Division of Infectious Diseases, USA	140	Retrospective cohort study	Total SSIs	12.9	16;23;24;27;46	7
Dosch *et al*.^29^	2019	USA	2014–2015	NR	NR	ACS-NSQIP database	6869	Retrospective cohort study	Organ/space SSIs	NR	25	8
									Superficial/deep incisional SSIs	NR	25	
Flick *et al*.^30^	2020	USA	2015–2018	Open	NR	ACS-NSQIP database	205	Retrospective cohort study	Superficial/deep incisional SSIs	11.2	26	9
Fromentin *et al*.^31^	2022	France	2010–2016	NR	CDC criteria	3 hospitals^b^	146	Retrospective cohort study	Total SSIs	33.6	27	9
Funamizu *et al*.^32^	2018	Japan	2008–2017	NR	NR	Kawaguchi Municipal Medical Center, Japan	106	Case–control study	Total SSIs	14.2	12;23;28;29	7
Funamizu *et al*.^33^	2020	Japan	2015–2019	NR	CDC criteria	Ageo Central General Hospital, Japan	93	Case–control study	Total SSIs	32.3	4;23;25;28;29	6
Gavazzi *et al*.^34^	2016	Italy	2010–2013	NR	CDC&IDSA criteria	Humanitas Research Hospital, Rozzano, Italy	178	Prospective cohort study	Total SSIs	20.8	NR	8
									Superficial incisional SSIs	11.8	NR	
									Deep incisional SSIs	9.0	11;12;16	
									Organ/space SSIs	27.0	NR	
Girgis *et al*.^35^	2017	USA	2011–2015	Robotic vs. Open	NR	Prospectively maintained surgical databases and patients’ electronic medical records	474	Retrospective cohort study	Total SSIs	25.9	12;30;31	8
Hall *et al*.^36^	2021	USA	2016–2018	Open vs. MIS	NR	ACS-NSQIP database	9665	Retrospective cohort study	Total SSIs	20.8	43	9
									Superficial incisional SSIs	7.1	43	
									Organ/space SSIs	14.6	43	
									Deep incisional SSIs	0.9	NR	
Hamidi *et al*.^37^	2021	USA	2014–2017	NR	NR	ACS-NSQIP database	5851	Retrospective cohort study	Superficial incisional SSIs	10.0	16	9
Hu *et al*.^38^	2021	China	2015–2019	NR	NNIS criteria	Second Affiliated Hospital of Chongqing Medical University	177	Case–control study	Total SSIs	49.2	9;25;29;33–36	7
Kato *et al*.^39^	2018	Japan	2014–2016	NR	CDC criteria	Hospital, Japan	51	Prospective cohort study	Total SSIs	27.5	25;36	8
									Organ/space SSIs	17.6	NR	
									Superficial/deep incisional SSIs	9.8	NR	
Kondo *et al*.^40^	2013	Japan	2007–2011	NR	CDC&IDSA criteria	Miyazaki University School of Medicine, Japan	116	Retrospective cohort study	Total SSIs	35.3	12;27;37;38	7
									Superficial incisional SSIs	14.7	NR	
									Deep incisional SSIs	3.4	NR	
									Organ/space SSIs	22.4	NR	
Kone *et al*.^41^	2022	USA	2016–2018	Open	NR	ACS-NSQIP database	9136	Retrospective cohort study	Total SSIs	23.0	27	8
									Superficial incisional SSIs	NR	27	
									Organ/space SSIs	NR	27	
Kumagai *et al*.^42^	2019	Japan	2015–2017	Open	CDC&IDSA criteria	Jikei Kashiwa Hospital, Japan	61	Retrospective cohort study	Organ/space SSIs	27.9	39;40	6
Lawrence *et al*.^43^	2019	USA	2016–2018	NR	CDC & NSQIP criteria	Memorial Sloan Kettering, USA	300	Prospective cohort study	Superficial/deep incisional SSIs	22.3	15;16;38;41;42	9
Lemke *et al*.^44^	2021	USA	2016–2016	NR	NR	ACS-NSQIP database	3430	Retrospective cohort study	Total SSIs	22.0	8;9;12;16;39;43–49	8
									Superficial incisional SSIs	7.3	NR	
									Deep incisional SSIs	0.9	NR	
									Organ/space SSIs	15.8	NR	
Liu *et al*.^45^	2019	USA	2016–2017	Open	NR	ACS-NSQIP database	5969	Case–control study	Total SSIs	20.3	27;50;51	6
									Superficial incisional SSIs	7.2	27;50;51	
									Organ/space SSIs	14.1	27;50;51	
Mangieri *et al*.^46^	2021	USA	2014–2016	NR	NR	ACS-NSQIP database	2371	Retrospective cohort study	Total SSIs	NR	7	6
									Organ/space SSIs	NR	7	
Mintziras *et al*.^47^	2020	Germany	2009–2019	NR	CDC criteria	University Hospital Marburg, Germany	170	Retrospective cohort study	Total SSIs	9.4	12;46;52	9
Morikane^48^	2017	Japan	2012–2014	Open	CDC criteria	JANIS database	4567	Case–control study	Total SSIs	27.9	8;9;38;47	4
Nassour *et al*.^49^	2018	USA	2014–2015	Open vs. MIS	NR	ACS-NSQIP database	1336	Retrospective cohort study	Total SSIs	20.5	30	7
									Superficial incisional SSIs	6.7	30	
									Deep incisional SSIs	2.2	30	
									Organ/space SSIs	13.4	30	
Pisters *et al*.^50^	2001	USA	1990–1999	NR	NR	Pancreatic and periampullary cancer database	300	Retrospective cohort study	Total SSIs	10.0	16	9
Poruk *et al*.^51^	2016	USA	2011–2014	NR	CDC criteria	Johns Hopkins Hospital, USA	679	Retrospective cohort study	Total SSIs	17.2	15–17;25;38;41;53	9
Shinkawa *et al*.^52^	2013	Japan	2004–2010	NR	CDC criteria	Osaka City University Hospital, Japan	64	Retrospective cohort study	Total SSIs	32.8	28;29;54	8
Suenaga *et al*.^53^	2020	Japan	2011–2015	NR	CDC criteria	Nagoya University Hospital, Japan	155	Prospective cohort study	Total SSIs	31.6	24;38;39;53;54	8
Sugimachi *et al*.^54^	2019	Japan	2014–2017	NR	NR	Kyushu Cancer Center, Japan	69	Retrospective cohort study	Organ/space SSIs	8.7	39;56	6
									Superficial/deep incisional SSIs	47.8	NR	
Sugiura *et al*.^55^	2012	Japan	2002–2010	NR	CDC criteria	Shizuoka Cancer Center, Japan	408	Retrospective cohort study	Total SSIs	51.0	NR	6
									Organ/space SSIs	48.0	12;29;38;43;48	
									Superficial/deep incisional SSIs	15.0	32;38;48	
Suragul *et al*.^56^	2020	Thailand	2010–2018	NR	CDC criteria	Ramathibodi Hospital, Thailand	280	Case–control study	Total SSIs	32.1	NR	7
									Organ/space SSIs	13.9	12;29;37	
									Superficial/deep incisional SSIs	18.2	16;29;37	
Sutton *et al*.^57^	2022	USA	2008–2020	NR	NR	ACS-NSQIP database	483	Retrospective cohort study	Total SSIs	32.9	9;16;33;57	6
									Organ/space SSIs	17.2	9;33;58	
									Superficial/deep incisional SSIs	19.0	16;57	
Tee *et al*.^58^	2021	USA	2016–2018	Open	ACS-NSQIP criteria	ACS-NSQIP database	11 562	Retrospective cohort study	Superficial incisional SSIs	7.5	NR	8
									Deep incisional SSIs	1.0	NR	
									Organ/space SSIs	16.8	NR	
									Superficial/deep incisional SSIs	8.5	4;8–10;12;15;16;20;21;23;27;38;39;43;45;47;48;50;51;56;68–73	
Yamamoto *et al*.^59^	2020	Japan	2009–2018	NR	JCOG PC criteria	Yokohama City University Hospital, Japan	326	Retrospective cohort study	Total SSIs	18.4	11;38;59;60	8
Yang *et al*.^60^	2019	China	2009–2016	Open	NR	Hospital, China	603	Retrospective cohort study	Total SSIs	3.2	9;16	9
Yang *et al*.^61^	2021	China	2018–2021	NR	CDC criteria	Nanjing DrumTower Hospital, China	284	Retrospective cohort study	Organ/space SSIs	29.6	23;61	7
									Superficial/deep incisional SSIs	5.6	16;62–66	
Yun *et al*.^62^	2021	USA	2015–2016	Open	CDC criteria	Johns Hopkins Hospital, USA	244	Retrospective cohort study	Total SSIs	11.1	4;67	6
Zorbas *et al*.^63^	2021	USA	2014–2016	NR	NR	ACS-NSQIP database	10 316	Retrospective cohort study	Total SSIs	22.5	12	7

a 1. Intraoperative drain placement; 2. length of drainage after surgery; 3. fluid retention; 4. diabetes mellitus; 5. non-diabetic endocrine comorbidity; 6. duct-to-mucosa pancreaticojejunostomy; 7. high-risk diagnosis; 8. age; 9. sex; 10. pulmonary disease; 11. cardiac disease; 12. body mass index (BMI); 13. preoperative bile duct catheterization; 14. prior abdominal surgery; 15. neoadjuvant therapy; 16. preoperative biliary stenting; 17. wound negative pressure dressing; 18. endoscopic retrograde cholangio-pancreatography exposure before pancreaticoduodenectomy (EEBPD); 19. preoperative low-density lipoprotein (LDL); 20. preoperative alkaline phosphatase (ALP); 21. preoperative creatinine (Cr); 22. hypertension; 23. preoperative albumin; 24. blood loss; 25. perioperative blood transfusion; 26. wound closure type; 27. antibiotic prophylaxis; 28. geriatric nutritional risk index; 29. postoperative pancreatic fistula; 30. surgical types; 31. Charlson comorbidity index; 32. visceral fat thickness; 33. tumor nature; 34. ascites; 35. postoperative cough; 36. Candida species; 37. preoperative cholangitis; 38. operation time; 39. pancreatic texture; 40. bile exposure; 41. preoperative jaundice; 42. use of perioperative bundle; 43. drainage techniques; 44. race; 45. preoperative weight loss; 46. American Society of Anesthesiology (ASA) score; 47. wound classification; 48. duct size; 49. pylorus preserving; 50. wound protector used; 51. incision type; 52. bile culture; 53. concomitant vascular resection; 54. nutritional risk screening; 55. RNA-targeted reverse transcriptase–quantitative polymerase chain reaction in preoperative blood; 56. smoking history; 57. year of operation; 58. vascular reconstruction; 59. desflurane used; 60. cerebrovascular disease; 61. *Staphylococcus epidermidis*; 62. *Klebsiella pneumoniae*; 63. *Staphylococcus aureus*; 64. *Enterococcus faecalis*; 65. *Enterococcus faecium*; 66. *Escherichia coli*; 67. abnormal blood sugar; 68. open wound; 69. Preoperative Systemic Inflammatory Response Syndrome; 70. preoperative Sodium; 71. preoperative aspartate amino-transferase (AST); 72. preoperative International Normalized Ratio (INR); 73. pancreatic reconstruction.

b Three hospitals including Cochin University Hospital Paris, France, Claude Huriez University Hospital Lille, France, and European Georges Pompidou University Hospital Paris, France.

ACS-NSQIP, American College of Surgeons National Surgical Quality Improvement Program; CDC, Centers for Disease Control and Prevention; IDSA, Infection Diseases Society of America; JANIS, Japan Nosocomial Infections Surveillance; JCOG PC, Japan Clinical Oncology Group Postoperative Complications ; MIS, minimally invasive surgery; NHSN, National Healthcare Safety Network; NNIS, National Nosocomial Infections Surveillance system; NR, not report.

### Prevalence of surgical site infection

A total of 43 studies^19–28,30–45,47–63^ were included in the analysis of the prevalence of SSIs. Two studies^29,46^ did not report it and thus were excluded. From a descriptive analysis perspective, the highest prevalence of SSIs was estimated in Japan. There were 51.0%^55^, 14.7%^40^, 3.4%^40^, 48.0%^55^, and 47.8%^54^ in total SSIs, superficial incisional SSIs, deep incisional SSIs, organ/space SSIs, and superficial/deep incisional SSIs, respectively (Table 1).

The pooled prevalence of different types of SSIs was 23% (95% CI: 0.19–0.27), 8% (95% CI: 0.07–0.09), 2% (95% CI: 0.01–0.03), 17% (95% CI: 0.14–0.21), and 16% (95% CI: 0.10–0.22) for total SSIs, superficial incisional SSIs, deep incisional SSIs, organ/space SSIs, and superficial/deep incisional SSIs, respectively (Fig. 1). These pooled results all have a significant level of heterogeneity. To identify the sources of heterogeneity, subgroup analysis in terms of region and study design as grouping variables was performed (Supplemental Digital Content 3, Fig. 2, http://links.lww.com/MS9/A300 and Fig. 3, http://links.lww.com/MS9/A300). The results showed that the prevalence of SSIs in Japan was the highest among all types of SSIs. The results of the sensitivity analysis were robust (Supplemental Digital Content 3, Fig. 4, http://links.lww.com/MS9/A300). There was potential publication bias among the included studies regardless of which method was used, except for studies analyzed in total SSIs, superficial incisional SSIs, and organ/space SSIs subgroups (Table 2; Supplemental Digital Content 3, Fig. 5, http://links.lww.com/MS9/A300).

Figure 1Forest plots of prevalence of SSIs. (A) Total SSIs. (B) Superficial incisional SSIs. (C) Deep incisional SSIs. (D) Organ/space SSIs. (E) Superficial/deep incisional SSIs.

**Figure:**
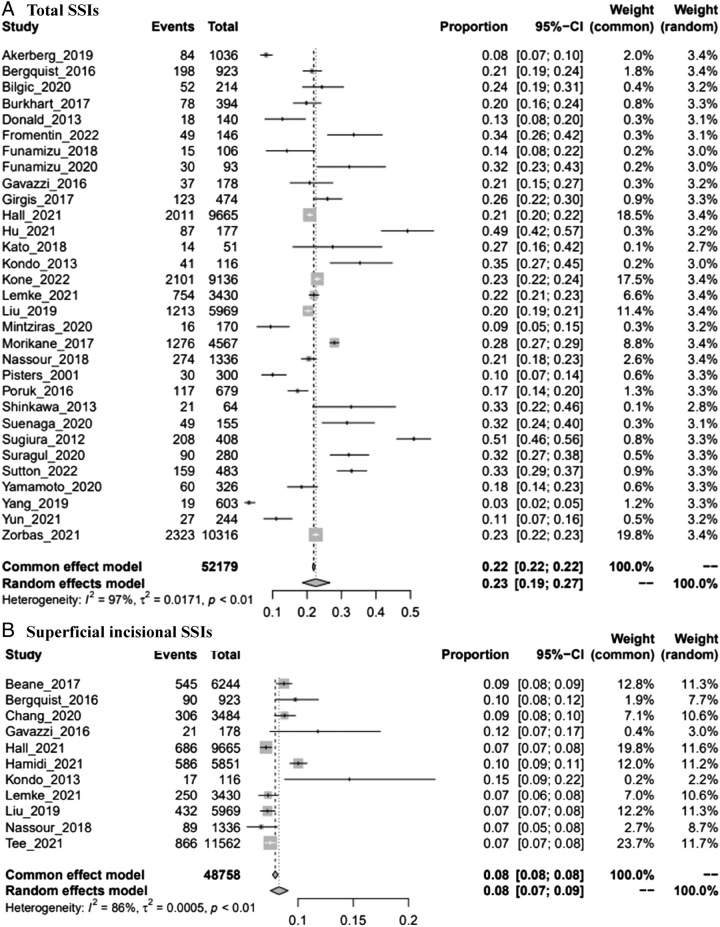

**Figure:**
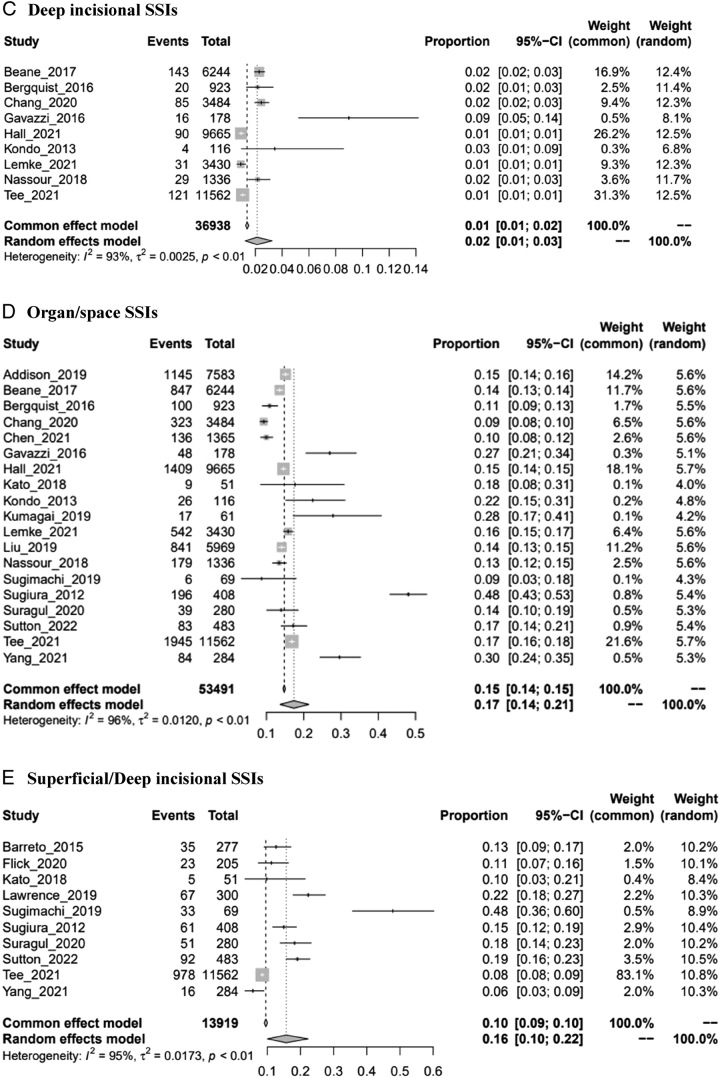

**Table 2:** Results of publication bias assessment of SSIs

	No. of studies	Egger’s test	Trim-and-fill method^a^
		*P*	OR (95% CI)
Prevalence			
Total SSIs	31	0.946	0.225 (0.187, 0.266)
Superficial incisional SSIs	11	0.227	0.078 (0.068, 0.088)
Deep incisional SSIs	9	0.081	0.012 (0.004, 0.024)
Organ/space SSIs	19	0.365	0.146 (0.105, 0.193)
Superficial/deep incisional SSIs	10	0.019	0.088 (0.038, 0.156)
Risk factors			
Preoperative biliary stenting	14	0.021	1.766 (1.282, 2.432)
BMI	12	0.003	1.067 (0.867, 1.314)
Antibiotic prophylaxis-1^b^	3	0.142	0.928 (0.871, 0.989)
Antibiotic prophylaxis-2^c^	3	0.053	0.753 (0.696, 0.813)
Operation time	8	0.002	1.026 (0.708, 1.488)
Perioperative blood transfusion	3	0.210	1.217 (1.068, 1.387)
Sex	5	0.481	1.196 (0.827, 1.729)
Postoperative pancreatic fistula	6	0.272	5.491 (3.546, 8.505)
Pancreatic texture	4	0.004	1.684 (0.354, 8.007)
Preoperative albumin	5	0.403	1.088 (0.225, 5.266)
Diabetes mellitus	5	0.568	0.864 (0.488, 1.530)
Cardiac disease	3	0.137	1.370 (0.340, 5.523)
Age	4	0.972	1.077 (0.799, 1.453)
ASA classification	3	0.756	1.067 (0.683, 1.667)
USA			
Preoperative biliary stenting	9	0.185	1.646 (1.132, 2.394)
BMI	6	0.007	1.059 (0.856, 1.310)
Japan			
Operation time	5	0.008	1.024 (0.503, 2.085)
Postoperative pancreatic fistula	4	0.222	7.738 (4.881, 12.270)

a Using random-effect model.

b Antibiotic prophylaxis-1: second-generation or third-generation cephalosporin vs. first-generation.

c Antibiotic prophylaxis-2: broad-spectrum antibiotics vs. first-generation.

ASA, American Society of Anesthesiologists; BMI, body mass index.

### Risk factors for SSI

A total of 73 risk factors for SSI were identified in the analysis. However, 60 factors were excluded without sufficient data for quantitative analysis. We included 13 risk factors that were reported in more than three studies for meta-analysis.

### Preoperative biliary stenting

The pooled effects of 14 studies^21,25,28,34,37,43,44,50,51,56–58,60,61^ suggested that patients who had preoperative biliary stents were 2.43 times more likely to develop SSIs than patients without biliary stents (OR=2.43, 1.86–3.19, *I* ^2^=74%) (Fig. 2A). The results of subgroup analysis showed that patients with preoperative biliary stents were more likely to develop SSIs regardless of the grouping variable, including region, study design, or type of SSIs (Supplemental Digital Content 3, Figs 6–8, http://links.lww.com/MS9/A300).

Figure 2Forest plots of risk factors of SSIs. ASA: American Society of Anesthesiologists; BMI: body mass index. *Author_year_#: please see Supplementary material Note (Supplemental Digital Content 3, http://links.lww.com/MS9/A300). (A) Preoperative biliary stenting. (B) BMI (high vs. low). (C1) Antibiotic prophylaxis (second-generation or third-generation cephalosporin vs first-generation). (C2) Antibiotic prophylaxis (broad-spectrum antibiotics vs. first generation). (D) Operation time (long vs. short). (E) Perioperative blood transfusion. (F) Sex (male vs. female). (G) Postoperative pancreatic fistula. (H) Pancreatic texture (soft vs. hard). (I) Preoperative albumin (low level vs. high). (J) Diabetes mellitus. (K) Cardiac disease. (L) Age (older vs. young). (M) ASA classification (≥3 score vs. <3 score).

**Figure:**
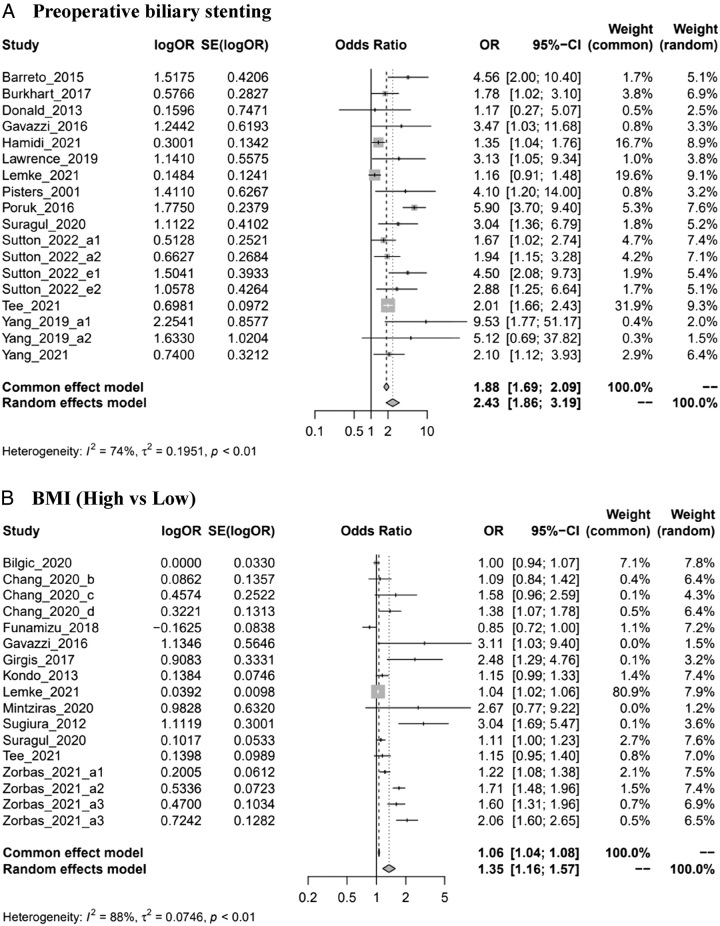

**Figure:**
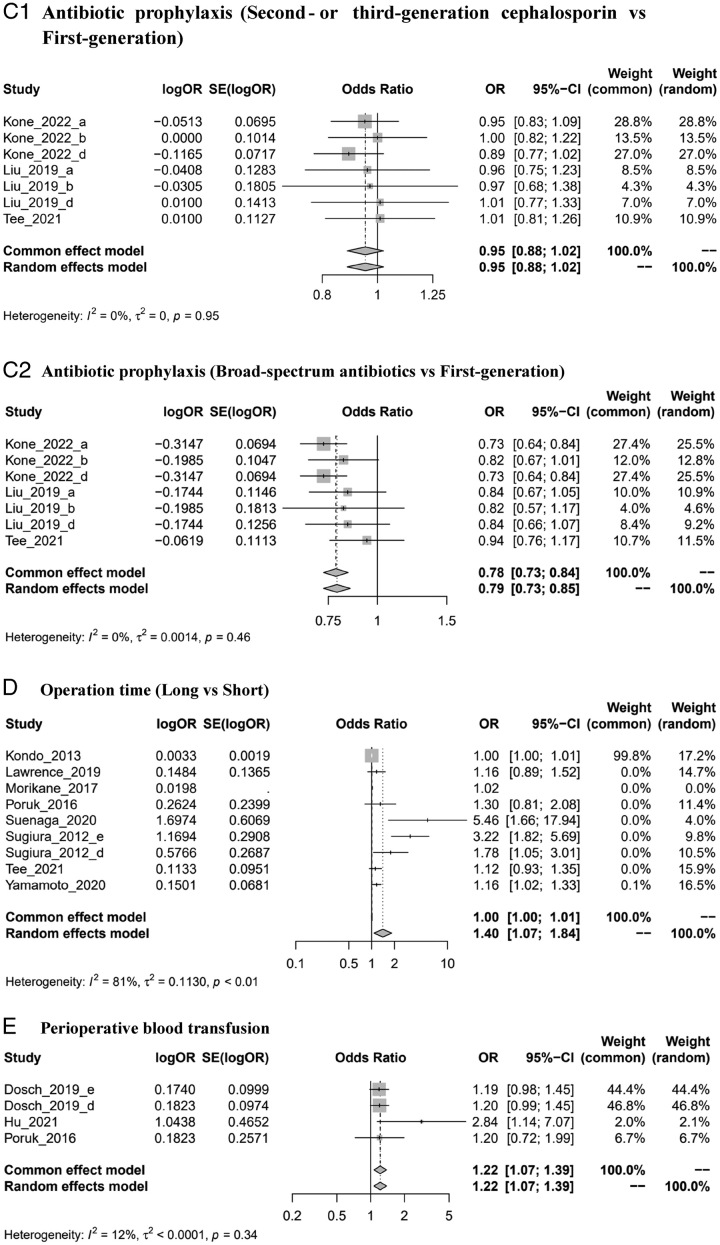

**Figure:**
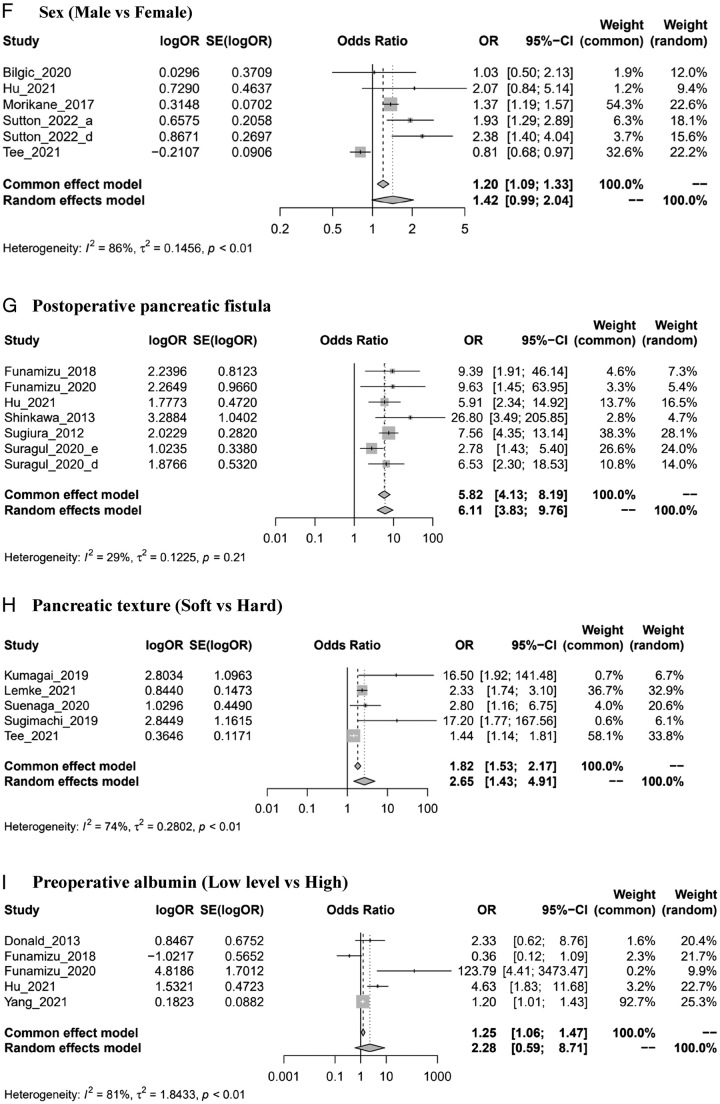

**Figure:**
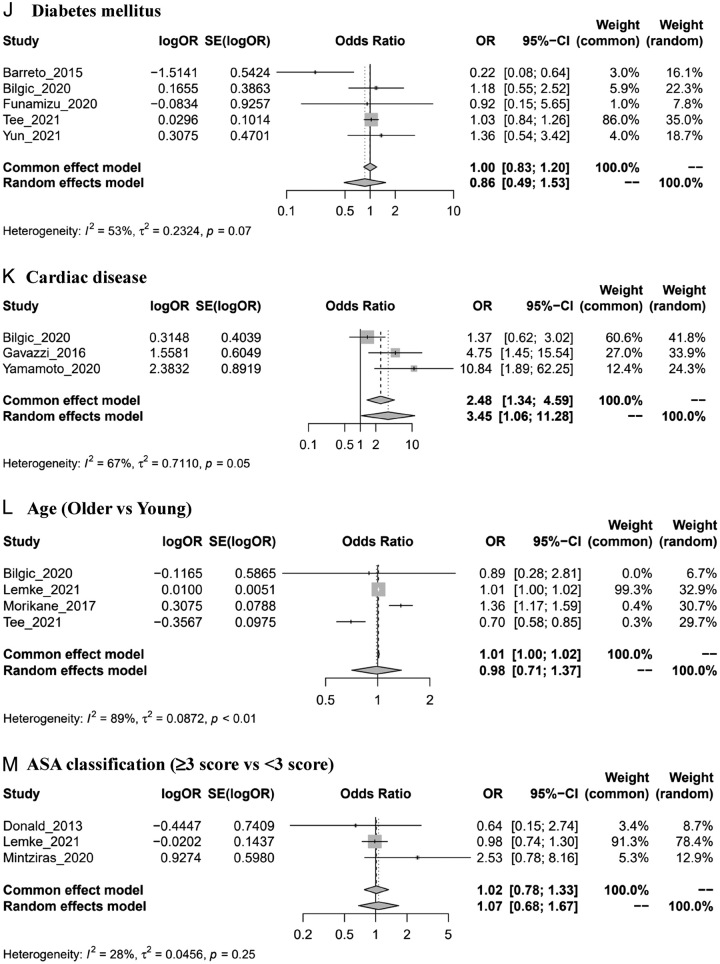

### Body mass index (BMI)

Classification standards for BMI cutoff were inconsistent or not clearly reported in the 12 included studies^24,26,32,34,35,40,44,47,55,56,58,63^. Therefore, higher BMI was not clearly defined in this study. According to the meta-analysis, people with a higher BMI were more likely to develop SSIs than those with a lower BMI (OR=1.35, 1.16–1.57, *I* ^2^=88%) (Fig. 2B). Except for the combined results in Japan (OR=1.37, 0.68–2.76, *I* ^2^=90%) and case–control studies (OR=0.98, 0.76–1.27, *I* ^2^=86%), the results of the other subgroup analysis suggested that higher BMI was a significant risk factor (Supplemental Digital Content 3, Figs 6–8, http://links.lww.com/MS9/A300).

### Antibiotic prophylaxis

Four studies^31,41,45,58^ reported on antibiotic prophylaxis. Of these, one study^31^ focused on extended antibiotic prophylaxis versus standard antibiotic prophylaxis was excluded. The results of the remaining three studies showed that the use of second-generation or third-generation cephalosporin could not reduce the prevalence of SSIs compared with first-generation cephalosporin (OR=0.95, 0.88–1.02, *I* ^2^=0%) (Fig. 2C1). However, the use of broad-spectrum antibiotics significantly reduced the prevalence of SSIs compared with first-generation cephalosporins (OR=0.79, 0.73–0.85, *I* ^2^=0%) (Fig. 2C2).

### Operation time

Although the eight included studies^40,43,48,51,53,55,58,59^ had inconsistent definitions of the longer operation time, we can roughly conclude that patients treated with longer operation time were 40% more likely to develop SSIs than patients whose operation time was shorter (OR=1.40, 1.07, 1.84, *I* ^2^=81%) (Fig. 2D). The subgroup analysis showed similar results. However, there were no statistically significant differences in the subgroups for the USA (OR=1.15, 0.99, 1.33, *I* ^2^=0%), total SSIs (OR=1.13, 0.95, 1.35, *I* ^2^=78%), and superficial/deep incisional SSIs (OR=1.53, 0.82, 2.87, *I* ^2^=83%) (Supplemental Digital Content 3, Figs 6–8, http://links.lww.com/MS9/A300).

### Perioperative blood transfusion

The meta-analysis of three studies^29,38,51^ showed that there was a significant correlation between perioperative blood transfusion and SSIs (OR=1.22, 1.07–1.39, *I* ^2^=12%) (Fig. 2E).

### Sex

Five studies^24,38,48,57,58^ were included in the analysis. The results suggested that there was a potentially higher prevalence of SSIs (OR=1.42, 0.99–2.04, *I* ^2^=86%) in males than in females, although the difference was not significant (Supplemental Digital Content 3, Fig. 2F, http://links.lww.com/MS9/A300). However, it was significant in the total SSI subgroup (OR=1.48, 1.18–1.86, *I* ^2^=23%) (Supplemental Digital Content 3, Figs 6–8, http://links.lww.com/MS9/A300).

### Postoperative pancreatic fistula

The pooled results of six studies^32,33,38,52,55,56^ suggested that patients with postoperative pancreatic fistula were 6.11 times more likely to develop SSIs than people without postoperative pancreatic fistula (OR=6.11, 3.83, 9.76, *I* ^2^=29%) (Fig. 2G). All subgroup analysis results were in line with the pooled result (Supplemental Digital Content 3, Figs 6–8, http://links.lww.com/MS9/A300).

### Pancreatic texture

Five studies^42,53,54,58^ were finally included in the analysis. The results showed that a soft pancreatic texture could increase the risk of developing SSIs by 2.65 times compared with a hard texture (OR=2.65, 1.43, 4.91, *I* ^2^=74%) (Fig. 2H). Similar results were observed in the subgroup analysis (Supplemental Digital Content 3, Figs 6–8, http://links.lww.com/MS9/A300).

### Preoperative albumin

A total of six studies^28,32,33,38,58,61^ evaluated the relationship between preoperative albumin and SSIs. Of these, one study^58^ was excluded due to the inconsistency of the direction of the reported effect (high levels of preoperative albumin compared to low levels). According to the meta-analysis of the remaining studies, people with lower levels of preoperative albumin were 2.28 times more likely to develop SSIs than patients with higher levels (OR=2.28, 0.59–8.71, *I* ^2^=81%), but the difference was not significant (Fig. 2I). Subgroup analyses revealed similar results (Supplemental Digital Content 3, Figs 6–8, http://links.lww.com/MS9/A300).

### Diabetes mellitus

Five studies^21,24,33,58,62^ were included in the meta-analysis. The results suggested that patients with diabetes mellitus were less likely to develop SSIs (OR=0.86, 0.49–1.53, *I* ^2^=53%), but the difference was not significant (Fig. 2J). The same results were observed in subgroup analyses (Supplemental Digital Content 3, Figs 6–8, http://links.lww.com/MS9/A300).

### Cardiac disease

The pooled results of three studies^24,34,59^ indicated that patients with cardiac disease were 3.45 times more likely to develop SSIs than those without cardiac disease (OR=3.45, 1.06–11.28, *I* ^2^=67%) (Fig. 2K).

### Age

Four studies^24,44,48,58^ with different definitions of older age were included in the analysis. The results suggested that older patients were more likely to suffer from SSIs than younger people (OR=0.98, 0.71–1.37, *I* ^2^=89%), although this result was not statistically significant (Fig. 2L). The same results were observed in each subgroup analysis (Supplemental Digital Content 3, Figs 6–8, http://links.lww.com/MS9/A300).

### American Society of Anesthesiologists (ASA) classification

The association between ASA score and SSIs was estimated in three studies^28,44,47^. The meta-analysis results showed that patients with an ASA score ≥3 were more likely to develop SSIs than those with an ASA score <3 (OR=1.07, 0.68–1.67, *I* ^2^=28%), but the difference was not statistically significant (Fig. 2M).

### Sensitivity analyses

The significance of the result changed when one of the three studies^42,53,54^ was removed at a time for pancreatic texture using a random-effects model. For the sex factor, removing Tee *et al*.^58^ from the analysis in the random-effects model and Morikane^48^ in the common-effects model changed the significance of the conclusion. The exclusion of the study of Gavazzi *et al*.^34^ or Yamamoto *et al*.^59^ in the analysis of cardiac disease using a random-effects model and the removal of Gavazzi *et al*.^34^ in the common-effects model resulted in a change in the significance of the conclusion. Other sensitivity analysis results showed that the pooled results were robust after omitting any of the studies once a time. The pooled results of any other risk factors did not change the significance, regardless of whether a random-effects model or common-effects model was used (Supplemental Digital Content 3, Fig. 9, http://links.lww.com/MS9/A300).

### Assessment of publication bias

There was a publication bias in the analysis of preoperative biliary stenting, BMI, operation time, broad-spectrum antibiotics, and pancreatic texture based on the asymmetric funnel plots. The results of Egger’s regression test also indicated publication bias (Table 2). The funnel plot asymmetry corrected using the trim-and-fill method suggested the existence of some publication bias and unrobust results (Table 2; Supplemental Digital Content 3, Fig. 10, http://links.lww.com/MS9/A300).

### Subgroup analysis of the USA and Japan studies

For studies conducted in the USA, the pooled prevalence of SSIs varied across different categories. In total SSIs, the prevalence was 20% (0.17–0.23, *I* ^2^=89%), while for superficial incisional SSIs, deep incisional SSIs, organ/space SSIs, and superficial/deep incisional SSIs, it was 8% (0.07–0.09, *I* ^2^=88%), 2% (0.01–0.02, *I* ^2^=94%), 14% (0.12–0.16, *I* ^2^=95%), and 15% (0.09–0.22, *I* ^2^=97%), respectively (Supplemental Digital Content 3, Fig. 11, http://links.lww.com/MS9/A300). Preoperative biliary stenting, higher BMI, longer operation time, perioperative blood transfusion, and cardiac disease were identified as statistically significant risk factors for SSIs, and broad-spectrum antibiotic use was a significant protective factor (Supplemental Digital Content 3, Fig. 6, http://links.lww.com/MS9/A300). For preoperative biliary stenting and BMI in the subgroup analysis, the results were consistent with the full sample results (Supplemental Digital Content 3, Fig. 12, http://links.lww.com/MS9/A300, and Fig. 13, http://links.lww.com/MS9/A300). Sensitivity analysis results also show that the results were robust (Supplemental Digital Content 3, Fig. 14, http://links.lww.com/MS9/A300). The evidence from funnel plot and Egger’s regression test shows that there was a publication bias in BMI analysis of USA (Table 2; Supplemental Digital Content 3, Fig. 15, http://links.lww.com/MS9/A300).

For studies carried out in Japan, the pooled prevalence of SSIs was 30% (0.23–0.37, *I* ^2^=94%) in total SSIs, 24% (0.12–0.39, *I* ^2^=95%) in organ/space SSIs, and 22% (0.05–0.47, *I* ^2^=95%) in superficial/deep incisional SSIs (Supplemental Digital Content 3, Fig. 16, http://links.lww.com/MS9/A300). Longer operation time, postoperative pancreatic fistula, and soft pancreatic texture were significant risk factors for SSIs. A similar result was found in further operation time and postoperative pancreatic fistula subgroup analysis (Supplemental Digital Content 3, Fig. 17, http://links.lww.com/MS9/A300, and Fig. 18, http://links.lww.com/MS9/A300). The results of the sensitivity analysis showed the robustness of the full sample analysis, except for removing Suenaga *et al*.^53^ or Sugiura *et al*.^55^ from the operation time analysis in the random-effects model (Supplemental Digital Content 3, Fig. 19, http://links.lww.com/MS9/A300). The publication bias was found in operative time analysis of Japan (Table 2; Supplemental Digital Content 3, Fig. 20, http://links.lww.com/MS9/A300).

## Discussion

In this study, we systematically estimated the prevalence of SSIs and evaluated the potential risk factors in patients undergoing PD. The analysis of 98 704 patients from 45 included studies suggested that SSIs were a serious postoperative complication after PD. The pooled prevalence of total SSIs was 23%. Preoperative biliary stenting, higher BMI, longer operation time, postoperative pancreatic fistula, soft pancreatic texture, perioperative blood transfusion, and cardiac disease were identified as significant risk factors for the development of SSIs after PD. However, antibiotic prophylaxis (second-generation or third-generation cephalosporin), sex, preoperative albumin, diabetes mellitus, age, and ASA score were not significant risk factors. Moreover, we identified the use of broad-spectrum antibiotics as a significant protective factor against SSIs. Sensitivity analysis showed the robustness of the base-case results.

To the best of our knowledge, few reviews and meta-analyses have focused on the prevalence of SSIs after PD. According to the included studies, the prevalence rate of SSIs after PD varied from 3.2% to 51.0%. The pooled total SSI rate was 23% (0.19–0.27, *I* ^2^=97%), which was consistent with findings from previous research, up to 23.5%^1^. For the SSI type, the meta-analysis results showed that the lowest prevalence was estimated in deep incisional SSIs (2%), and the highest prevalence was in organ/space SSIs (17%), which is similar to the published studies^22,23,26^. In terms of the region-specific SSI rate, Japan was reported to have the highest prevalence of SSIs compared with the United States, China, and other countries.

Several studies^8–11,64–66^ have explored the potential risk factors associated with SSIs after PD; however, they were limited to a narrow scope with a particular factor. We systematically reviewed the risk factors in the analysis. Preoperative biliary stent placement is a surgical procedure used for preoperative biliary drainage in patients with obstructive jaundice who are about to undergo neoadjuvant chemotherapy^1^. Preoperative biliary stenting has been demonstrated to increase the development of postoperative SSIs in previous studies^8,9^, which was consistent with our findings. A possible explanation is the transport of enteric bacteria from bile, which can cause bacteremia and then lead to infections^1^. A longer duration of drainage could be a potential risk factor for SSIs, although the optimal duration of drainage has not been determined^19^. The superiority of metal stents with fewer stent-related complications compared to plastic stents has been demonstrated^67^. The application of the common bile duct should be strictly limited to clear indications^68^. Thus, preoperative biliary stent placement using metal stents should be considered a priority.

A higher BMI may increase the risk of developing SSIs after PD, which is similar to other abdominal surgery studies^69^. Although BMI cannot directly and accurately reflect the body’s fat composition, it can reflect the thickness of subcutaneous fat and the area of visceral fat to a certain extent. BMI may be a reliable parameter that has an impact on infection after laparotomy^70,71^.

It is noteworthy that compared with first-generation cephalosporins, there was no statistically significant protective effect of second-generation or third-generation cephalosporins against SSIs, while broad-spectrum antibiotics show a protective effect. One study^10^ also indicated that the use of targeted broad-spectrum antibiotics was linked to a significantly lower prevalence of SSIs after PD compared to standard antibiotic therapy. This is important for patients undergoing preoperative biliary drainage^41^.

As expected, a longer operating time was an independent risk factor for SSIs. Usually, a longer operation time leads to a longer exposure time to the environment and an increase in blood loss. In addition, a longer operation time itself may also reflect the complexity of the surgical procedure. A positive linear relationship between operation time and SSIs was demonstrated in a previous study^72^. In addition, there was a strong relationship between perioperative blood transfusion and the development of SSIs after PD. Blood transfusions may lead to immunosuppression and transmission of pathogens^73,74^. Therefore, it is recommended that clinicians prioritize their surgical skills, aim to decrease operation time, and minimize both intraoperative blood loss and perioperative blood transfusions.

Postoperative pancreatic fistula was found to be an independent risk factor for SSIs in our analysis. Pancreatic fistula is one of the most common complications after PD^75^. Pancreatic fistula can cause pancreatic secretions (digestive enzymes and other substances) to leak into the surrounding tissues or abdominal cavity, leading to SSIs. For the texture of the pancreas, soft pancreas is a risk factor for SSIs compared to hard pancreas. A soft pancreas is leaked more easily during and after PD than a hard pancreas, which can increase the risk of infection^76,77^.

Although not statistically significant, our analysis found that preoperative albumin was a potential risk factor for SSIs. Albumin could be used as an indicator of nutritional status. Low levels of albumin often suggest malnutrition, which can increase the risk of infection^78^.

Contrary to the findings of previous studies on the risk factors for SSIs, our analysis did not find a significant association between diabetes mellitus and increased susceptibility to SSIs^69,79^. Similarly, male sex was not a significant risk factor in our study. Cardiac disease can be a risk factor for SSIs. Kent *et al*.^80^ also found that coronary artery disease is a predictive factor for SSIs in a prospective study.

The ASA score is a classification system of the ASA based on the patient’s physical condition and the risk of surgery. The results showed that an ASA score ≥3 was not significantly associated with an increased risk of developing SSIs. This finding is inconsistent with previous research, which has suggested that a higher ASA score is linked to a higher risk of SSIs^69^. Similarly, older age, although a potential risk factor for SSIs, was not significant in our study or in previous studies^64,65^.

In addition to the established risk factors for SSIs after PD, our analysis suggested some other potential risk factors that have not been extensively explored (Supplemental Digital Content 3, Fig. 21, http://links.lww.com/MS9/A300). To explore these potential risk factors, we used two different approaches. One method was to treat different types of SSIs as separate studies within the same study, thus categorizing at least three independent groups. Pancreatic duct not over 3 mm compared to over 6 mm, unknown incision compared to midline incision, and preoperative cholangitis were risk factors for SSIs. Robot-assisted surgery compared with open surgery and wound protection were identified as protective factors against SSIs. The second method was to conduct subgroup analysis using studies that did not clearly specify their category. For example, we analyzed the results of neoadjuvant therapy under both neoadjuvant chemotherapy and neoadjuvant radiotherapy categories. The pooled effects showed that neoadjuvant chemotherapy, contaminated wounds compared to clean wounds, and dirty wounds compared to clean wounds were risk factors for SSIs, while neoadjuvant radiotherapy was not a significant risk factor. In addition to wound protector use^11^ and robot-assisted surgery^81^, which have been shown to be protective factors for SSIs after PD, further research is required to investigate other potential risk factors. Additionally, Vasavada and Patel^66^ also found that laparoscopic PD was associated with fewer SSIs than open PD.

There are some limitations. First, only English-published studies were included, which may limit the findings. Second, not all included studies were designed to report prevalence rates, which can lead to inaccuracies in the merged data. Third, it is important to note that some studies rely on retrospective analysis, which may be affected by nonrandomized strategies and unmeasured confounding factors. Fourth, although sensitivity and subgroup analysis were performed, heterogeneity among the included studies cannot be eliminated, especially for the unclear or inconsistent criteria of SSI diagnosis, surgical approach, the cause of the surgery, and the baseline characteristics variation of each study.

## Conclusion

The prevalence rate of SSIs remains high, and potential risk factors related to SSIs should be given more attention. Regional variation exists, and a higher prevalence is observed in Japan. More efforts should be devoted to monitoring and preventing the development of SSIs in different regions, and more prospective studies are needed to assess potential influencing factors in the future.

## Ethical approval

As this is a systematic review and meta-analysis, the study did not require ethical approval.

## Consent

Not applicable as it is a meta-analysis study.

## Sources of funding

None.

## Author contribution

H.H., A.M., and H.L.: concept and design; H.H., Y.Q., Y.L., W.L., R.M., and X.Z.: acquisition, analysis, or interpretation of data; H.H., T.Z., and H.L.: statistical analysis; H.H., T.Z., Y.Q., and Y.L.: drafting of the manuscript; A.M. and H.L.: critical revision of the manuscript for important intellectual content; A.M. and H.L.: administrative, technical, or material support and supervision. All authors contributed and approved the final version of the manuscript.

## Conflicts of interest disclosure

The authors declare no conflicts of interest.

## Research registration unique identifying number (UIN)

1. Name of the registry used: Prevalence of and risk factors for surgical site infections after pancreaticoduodenectomy: a systematic review and meta-analysis.
2. Unique identifying number or registration ID: reviewregistry1669.
3. Hyperlink to your specific registration (must be publicly accessible and will be checked): https://www.researchregistry.com/browse-the-registry#registryofsystematicreviewsmetaanalyses/registryofsystematicreviewsmeta-analysesdetails/64b8b0e94d51160027dc2c21/.

## Guarantor

Hongfei Hu, MD; Aixia Ma, PhD; Hongchao Li, PhD.

## Data availability statement

As this is a systematic literature review, the data supporting the findings of this study are available within the published article and/or its supplementary materials.

## Provenance and peer review

Not commissioned, externally peer-reviewed.

## Assistance with the study

None.

## Presentation

None.

## Supplementary Material

SUPPLEMENTARY MATERIAL
