# Supplementary material for: Prevalence of and risk factors for surgical site infections after pancreaticoduodenectomy: a systematic review and meta-analysis
Source: Ann Med Surg (Lond). 2023 Nov 7;86(1):439–55. doi: 10.1097/MS9.0000000000001455 (PMC10783382; doi:10.1097/MS9.0000000000001455)
Supplement: SUPPLEMENTARY MATERIAL [file ms9-86-439-s003.docx]

**Prevalence of and risk factors for surgical site infections after pancreaticoduodenectomy: A systematic review and meta-analysis**

**Supplemental Digital Content 3**

**Table 1.** Detailed search strategies of each database

**Table 2.** Quality assessment of included studied using NOS

**Figure 1.** PIRSMA Flowchart of the study selection

**Figure 2.** Forest plots of prevalence of SSIs grouped by region

**Figure 3.** Forest plots of prevalence of SSIs grouped by study design

**Figure 4.** Sensitivity analysis graphs of prevalence of SSIs

**Figure 5.** Publication bias assessment graphs of prevalence of SSIs

**Figure 6.** Forest plots of risk factors of SSIs grouped by region

**Figure 7.** Forest plots of risk factors of SSIs grouped by study design

**Figure 8.** Forest plots of risk factors of SSIs grouped by type of SSIs

**Figure 9.** Sensitivity analysis graphs of risk factors of SSIs

**Figure 10.** Publication bias assessment graphs of risk factors of SSIs

**Figure 11.** Forest plots of prevalence of SSIs in USA

**Figure 12.** Forest plot of risk factors grouped by study design in USA

**Figure 13.** Forest plots of risk factors grouped by type of SSIs in USA

**Figure 14.** Sensitivity analysis of risk factors of SSIs in USA

**Figure 15.** Publication bias assessment graphs of risk factors of SSIs in USA

**Figure 16.** Forest plots of prevalence of SSIs in Japan

**Figure 17.** Forest plot of risk factors grouped by study design in Japan

**Figure 18.** Forest plot of risk factors grouped by type of SSIs in Japan

**Figure 19.** Sensitivity analysis of risk factors of SSIs in Japan

**Figure 20.** Publication bias assessment graphs of risk factors of SSIs in Japan

**Figure 21.** Forest plot of risk factors of SSIs based on insufficient evidence

# Note

| **Author_year_a/b/c/d/e** | |
| --- | --- |
| a | Total SSIs |
| b | Superficial incisional SSIs |
| c | Deep incisional SSIs |
| d | Organ/space SSIs |
| e | Superficial/Deep incisional SSIs |
| **Special case** | |
| Sutton_2022_a1 | Total SSIs; Plastic stent |
| Sutton_2022_a2 | Total SSIs; Mental stent |
| Sutton_2022_e1 | Superficial/Deep incisional SSIs; Plastic stent |
| Sutton_2022_e2 | Superficial/Deep incisional SSIs; Mental stent |
| Yang_2019_a1 | Total SSIs; Short duration stent |
| Yang_2019_a2 | Total SSIs; Long duration stent |
| Zorbas_2021_a1 | Total SSIs; BMI: 25-29.9 |
| Zorbas_2021_a2 | Total SSIs; BMI: 30-34.9 |
| Zorbas_2021_a3 | Total SSIs; BMI: 35-39.9 |
| Zorbas_2021_a3 | Total SSIs; BMI: ≥40 |

# Detailed search strategies for each database

**Table 1.** Detailed search strategies of each database

| **Number** | **Search Details** | | | **Results** | |
| --- | --- | --- | --- | --- | --- |
| **PubMed** | | | | | |
| #1 | "Surgical Wound Infection". MeSH Terms | | | 39,704 | |
| #2 | "Surgical Wound Infection" OR "Surgical Wound Infections" OR "Infection, Surgical Wound" OR "Infections, Surgical Wound" OR "Wound Infection, Surgical" OR "Wound Infections, Surgical" OR "Surgical Site Infection" OR "Surgical Site Infections" OR "Infection, Surgical Site" OR "Infections, Surgical Site" OR "Wound Infection, Postoperative" OR "Wound Infections, Postoperative" OR "Infection, Postoperative Wound" OR "Infections, Postoperative Wound" OR "Postoperative Wound Infections" OR "Postoperative Wound Infection" OR "Post-operative Wound Infection" OR "Post-operative Wound Infections" OR "Surgical Infection" OR "Surgical Infections" OR "Infection, Surgical" OR "Infections, Surgical". Title/Abstract | | | 20,808 | |
| #3 | #1 or #2 | | | 49,241 | |
| #4 | "Pancreaticoduodenectomy". MeSH Terms | | | 9,765 | |
| #5 | "Pancreaticoduodenectomy" OR "Pancreaticoduodenectomies" OR "Pancreatoduodenectomy" OR "Pancreatoduodenectomies" OR "Duodenopancreatectomy" OR "Duodenopancreatectomies" OR "Brunschwig Operation" OR "Pancreatico Duodenectomy" OR "Pancreato Duodenal Resection" OR "Pancreato Duodenectomy" OR "Pancreatoduodenal Resection" OR "Whipple Operation" OR "Whipple Procedure" OR "Whipple Resection". Title/Abstract | | | 13,884 | |
| #6 | #4 OR #5 | | | 15,816 | |
| #7 | "Risk". MeSH Terms | | | 1,359,579 | |
| #8 | "Risk" OR "Risks" OR "Predictor" OR "Predictors" OR "Predictive factor" OR "Predictive factors". Title/Abstract | | | 3,069,191 | |
| #9 | #7 OR #8 | | | 3,522,009 | |
| #10 | #3 AND #6 AND #9 | | | **168** | |
| **EMBASE** | | | | |  |
| #1 | | 'surgical infection'/exp | 61,766 | |  |
| #2 | | 'surgical wound infection' OR 'surgical wound infections' OR 'infection, surgical wound' OR 'infections, surgical wound' OR 'wound infection, surgical' OR 'wound infections, surgical' OR 'surgical site infection' OR 'surgical site infections' OR 'infection, surgical site' OR 'infections, surgical site' OR 'wound infection, postoperative' OR 'wound infections, postoperative' OR 'infection, postoperative wound' OR 'infections, postoperative wound' OR 'postoperative wound infections' OR 'postoperative wound infection' OR 'post-operative wound infection' OR 'post-operative wound infections' OR 'surgical infection' OR 'surgical infections' OR 'infection, surgical' OR 'infections, surgical':ti,ab,kw | 71,012 | |  |
| #3 | | #1 OR #2 | 71,012 | |  |
| #4 | | 'pancreaticoduodenectomy'/exp | 26,649 | |  |
| #5 | | 'pancreaticoduodenectomy' OR 'pancreaticoduodenectomies' OR 'pancreatoduodenectomy' OR 'pancreatoduodenectomies' OR 'duodenopancreatectomy' OR 'duodenopancreatectomies' OR 'brunschwig operation' OR 'pancreatico duodenectomy' OR 'pancreato duodenal resection' OR 'pancreato duodenectomy' OR 'pancreatoduodenal resection' OR 'total pancreatic duodenectomy' OR 'whipple operation' OR 'whipple procedure' OR 'whipple resection':ti,ab,kw | 29,947 | |  |
| #6 | | #4 OR #5 | 29,947 | |  |
| #7 | | 'risk'/exp | 2,936,695 | |  |
| #8 | | 'Risk' OR 'Risks' OR 'Predictor' OR 'Predictors' OR 'Predictive factor' OR 'Predictive factors':ti,ab,kw | 5,271,705 | |  |
| #9 | | #7 OR #8 | 5,287,134 | |  |
| #10 | | #3 AND #6 AND #9 | **469** | |  |
| **Cochran Library** | | | | |  |
| #1 | | "Surgical Wound Infection". MeSH Terms | | 3,677 |  |
| #2 | | "Surgical Wound Infection" OR "Surgical Wound Infections" OR "Infection, Surgical Wound" OR "Infections, Surgical Wound" OR "Wound Infection, Surgical" OR "Wound Infections, Surgical" OR "Surgical Site Infection" OR "Surgical Site Infections" OR "Infection, Surgical Site" OR "Infections, Surgical Site" OR "Wound Infection, Postoperative" OR "Wound Infections, Postoperative" OR "Infection, Postoperative Wound" OR "Infections, Postoperative Wound" OR "Postoperative Wound Infections" OR "Postoperative Wound Infection" OR "Post-operative Wound Infection" OR "Post-operative Wound Infections" OR "Surgical Infection" OR "Surgical Infections" OR "Infection, Surgical" OR "Infections, Surgical":ti,ab,kw | | 7,361 |  |
| #3 | | #1 or #2 | | 7,361 |  |
| #4 | | "Pancreaticoduodenectomy". MeSH Terms | | 306 |  |
| #5 | | "Pancreaticoduodenectomy" OR "Pancreaticoduodenectomies" OR "Pancreatoduodenectomy" OR "Pancreatoduodenectomies" OR "Duodenopancreatectomy" OR "Duodenopancreatectomies" OR "Brunschwig Operation" OR "Pancreatico Duodenectomy" OR "Pancreato Duodenal Resection" OR "Pancreato Duodenectomy" OR "Pancreatoduodenal Resection" OR "Total Pancreatic Duodenectomy" OR "Whipple Operation" OR "Whipple Procedure" OR "Whipple Resection":ti,ab,kw | | 1,294 |  |
| #6 | | #4 OR #5 | | 1,294 |  |
| #7 | | "Risk". MeSH Terms | | 39,714 |  |
| #8 | | "Risk" OR "Risks" OR "Predictor" OR "Predictors" OR "Predictive factor" OR "Predictive factors":ti,ab,kw | | 303,650 |  |
| #9 | | #7 OR #8 | | 306,261 |  |
| #10 | | #3 AND #6 AND #9 | | **36** |  |
| **Web of Science** | | | | |  |
| #1 | | "Surgical Wound Infection" OR "Surgical Wound Infections" OR "Infection, Surgical Wound" OR "Infections, Surgical Wound" OR "Wound Infection, Surgical" OR "Wound Infections, Surgical" OR "Surgical Site Infection" OR "Surgical Site Infections" OR "Infection, Surgical Site" OR "Infections, Surgical Site" OR "Wound Infection, Postoperative" OR "Wound Infections, Postoperative" OR "Infection, Postoperative Wound" OR "Infections, Postoperative Wound" OR "Postoperative Wound Infections" OR "Postoperative Wound Infection" OR "Post-operative Wound Infection" OR "Post-operative Wound Infections" OR "Surgical Infection" OR "Surgical Infections" OR "Infection, Surgical" OR "Infections, Surgical". ti,ab | 24,016 | |  |
| #2 | | "Pancreaticoduodenectomy" OR "Pancreaticoduodenectomies" OR "Pancreatoduodenectomy" OR "Pancreatoduodenectomies" OR "Duodenopancreatectomy" OR "Duodenopancreatectomies" OR "Brunschwig Operation" OR "Pancreatico Duodenectomy" OR "Pancreato Duodenal Resection" OR "Pancreato Duodenectomy" OR "Pancreatoduodenal Resection" OR "Total Pancreatic Duodenectomy" OR "Whipple Operation" OR "Whipple Procedure" OR "Whipple Resection". ti,ab | 16,272 | |  |
| #3 | | "Risk" OR "Risks" OR "Predictor" OR "Predictors" OR "Predictive factor" OR "Predictive factors". ti,ab | 5,034,952 | |  |
| #4 | | #1 AND #2 AND #3 | **117** | |  |
| **Science Direct** | | | | |  |
| #1 | | ("Wound Infection" OR "Surgical Site Infection" OR "Surgical Infection") AND ("Pancreaticoduodenectomy" OR "Pancreatoduodenectomy" OR "Duodenopancreatectomy" OR "Whipple") AND ("Risk" OR "Predictor" ) | | **85** |  |

# Quality assessment of included studies using Newcastle Ottawa scale (NOS)

**Table 2.** Quality assessment of included studied using NOS

| **Cohort study** | | | | | | | | | | | | | | | | | |
| --- | --- | --- | --- | --- | --- | --- | --- | --- | --- | --- | --- | --- | --- | --- | --- | --- | --- |
| **Study** | | **SELECTION** | | | | | | | **COMPARABILITY** | | **OUTCOME** | | | | | | **Quality Score** |
|  |  | Representativeness of the exposed cohort | | Selection of the non exposed cohort | Ascertainment of exposure | | Demonstration that outcome of interest was not present at start of study | | Comparability of cohorts on the basis of the design or analysis | | Assessment of outcome | | Was follow-up long enough for outcomes to occur | | Adequacy of follow up of cohorts | |  |
| **Addison-2019** | | ☆ | | ☆ | ☆ | | ☆ | | ☆☆ | | ☆ | | ☆ | | - | | **8** |
| **Akerberg-2019** | | ☆ | | ☆ | ☆ | | ☆ | | ☆☆ | | ☆ | | - | | ☆ | | **8** |
| **Beane-2017** | | ☆ | | ☆ | ☆ | | ☆ | | ☆☆ | | ☆ | | ☆ | | - | | **8** |
| **Bergquist-2016** | | ☆ | | ☆ | ☆ | | ☆ | | ☆☆ | | ☆ | | ☆ | | - | | **8** |
| **Bilgic-2020** | | ☆ | | ☆ | ☆ | | ☆ | | ☆☆ | | ☆ | | - | | - | | **7** |
| **Chang-2020** | | - | | ☆ | ☆ | | ☆ | | ☆☆ | | ☆ | | ☆ | | - | | **7** |
| **Chen-2021** | | ☆ | | ☆ | ☆ | | ☆ | | ☆☆ | | ☆ | | ☆ | | - | | **8** |
| **Donald-2013** | | - | | ☆ | ☆ | | ☆ | | ☆☆ | | ☆ | | ☆ | | - | | **7** |
| **Dosch-2019** | | ☆ | | ☆ | ☆ | | ☆ | | ☆☆ | | ☆ | | ☆ | | - | | **8** |
| **Flick-2020** | | ☆ | | ☆ | ☆ | | ☆ | | ☆☆ | | ☆ | | ☆ | | ☆ | | **9** |
| **Fromentin-2022** | | ☆ | | ☆ | ☆ | | ☆ | | ☆☆ | | ☆ | | ☆ | | ☆ | | **9** |
| **Gavazzi-2016** | | - | | ☆ | ☆ | | ☆ | | ☆☆ | | ☆ | | ☆ | | ☆ | | **8** |
| **Girgis-2017** | | - | | ☆ | ☆ | | ☆ | | ☆☆ | | ☆ | | ☆ | | ☆ | | **8** |
| **Hall-2021** | | ☆ | | ☆ | ☆ | | ☆ | | ☆☆ | | ☆ | | ☆ | | ☆ | | **9** |
| **Hamidi-2021** | | ☆ | | ☆ | ☆ | | ☆ | | ☆☆ | | ☆ | | ☆ | | ☆ | | **9** |
| **Kato-2018** | | - | | ☆ | ☆ | | ☆ | | ☆☆ | | ☆ | | ☆ | | ☆ | | **8** |
| **Kondo-2013** | | - | | ☆ | ☆ | | ☆ | | ☆☆ | | ☆ | | ☆ | | - | | **7** |
| **Kone-2022** | | ☆ | | ☆ | ☆ | | ☆ | | ☆☆ | | ☆ | | ☆ | | - | | **8** |
| **Kumagai-2019** | | - | | ☆ | ☆ | | ☆ | | ☆ | | ☆ | | ☆ | | - | | **6** |
| **Lawrence-2019** | | ☆ | | ☆ | ☆ | | ☆ | | ☆☆ | | ☆ | | ☆ | | ☆ | | **9** |
| **Lemke-2021** | | ☆ | | - | ☆ | | ☆ | | ☆☆ | | ☆ | | ☆ | | ☆ | | **8** |
| **Mangieri-2021** | | ☆ | | - | ☆ | | ☆ | | ☆☆ | | ☆ | | - | | - | | **6** |
| **Mintziras-2020** | | ☆ | | ☆ | ☆ | | ☆ | | ☆☆ | | ☆ | | ☆ | | ☆ | | **9** |
| **Nassour-2018** | | ☆ | | - | ☆ | | ☆ | | ☆☆ | | ☆ | | ☆ | | - | | **7** |
| **Pisters-2001** | | ☆ | | ☆ | ☆ | | ☆ | | ☆☆ | | ☆ | | ☆ | | ☆ | | **9** |
| **Poruk-2016** | | ☆ | | ☆ | ☆ | | ☆ | | ☆☆ | | ☆ | | ☆ | | ☆ | | **9** |
| **Shinkawa-2013** | | ☆ | | ☆ | ☆ | | ☆ | | ☆☆ | | ☆ | | ☆ | | - | | **8** |
| **Suenaga-2021** | | ☆ | | ☆ | ☆ | | ☆ | | ☆☆ | | ☆ | | - | | ☆ | | **8** |
| **Sugimachi-2019** | | ☆ | | ☆ | ☆ | | ☆ | | ☆ | | ☆ | | - | | - | | **6** |
| **Sugiura-2012** | | ☆ | | ☆ | ☆ | | ☆ | | ☆ | | ☆ | | - | | - | | **6** |
| **Sutton-2022** | | ☆ | | - | ☆ | | ☆ | | ☆☆ | | ☆ | | - | | - | | **6** |
| **Tee-2021** | | ☆ | | - | ☆ | | ☆ | | ☆☆ | | ☆ | | ☆ | | ☆ | | **8** |
| **Yamamoto-2020** | | ☆ | | ☆ | ☆ | | ☆ | | ☆☆ | | ☆ | | ☆ | | - | | **8** |
| **Yang-2019** | | ☆ | | ☆ | ☆ | | ☆ | | ☆☆ | | ☆ | | ☆ | | ☆ | | **9** |
| **Yang-2021** | | ☆ | | ☆ | ☆ | | ☆ | | ☆☆ | | ☆ | | - | | - | | **7** |
| **Yun-2021** | | ☆ | | ☆ | ☆ | | ☆ | | ☆ | | ☆ | | - | | - | | **6** |
| **Zorbas-2021** | | ☆ | | - | ☆ | | ☆ | | ☆ | | ☆ | | ☆ | | ☆ | | **7** |
| **Cohort study** | | | | | | | | | | | | | | | | | |
| **Study** | **SELECTION** | | | | | | | | | **COMPARABILITY** | | **EXPOSURE** | | | | | **Quality Score** |
|  | Is the case definition adequate? | | Representativeness of the cases | | | Selection of Controls | | Definition of Controls | | Comparability of cases and controls on the basis of the design or analysis | | Ascertainment of exposure | | Same method of ascertainment for cases and controls | | Non-Response rate |  |
| **Barreto-2015** | ☆ | | ☆ | | | ☆ | | - | | ☆☆ | | ☆ | | ☆ | | - | **7** |
| **Burkhart-2017** | ☆ | | - | | | ☆ | | ☆ | | ☆☆ | | ☆ | | ☆ | | - | **7** |
| **Funamizu-2018** | - | | ☆ | | | ☆ | | ☆ | | ☆☆ | | ☆ | | ☆ | | - | **7** |
| **Funamizu-2020** | - | | ☆ | | | - | | ☆ | | ☆☆ | | ☆ | | ☆ | | - | **6** |
| **Hu-2021** | ☆ | | ☆ | | | - | | ☆ | | ☆☆ | | ☆ | | ☆ | | - | **7** |
| **Liu-2019** | ☆ | | ☆ | | | ☆ | | - | | ☆☆ | | - | | ☆ | | - | **6** |
| **Morikane-2017** | - | | ☆ | | | - | | - | | ☆ | | ☆ | | ☆ | | - | **4** |
| **Suragul-2020** | ☆ | | ☆ | | | - | | ☆ | | ☆☆ | | ☆ | | ☆ | | - | **7** |

# PIRSMA Flowchart of the study selection

| 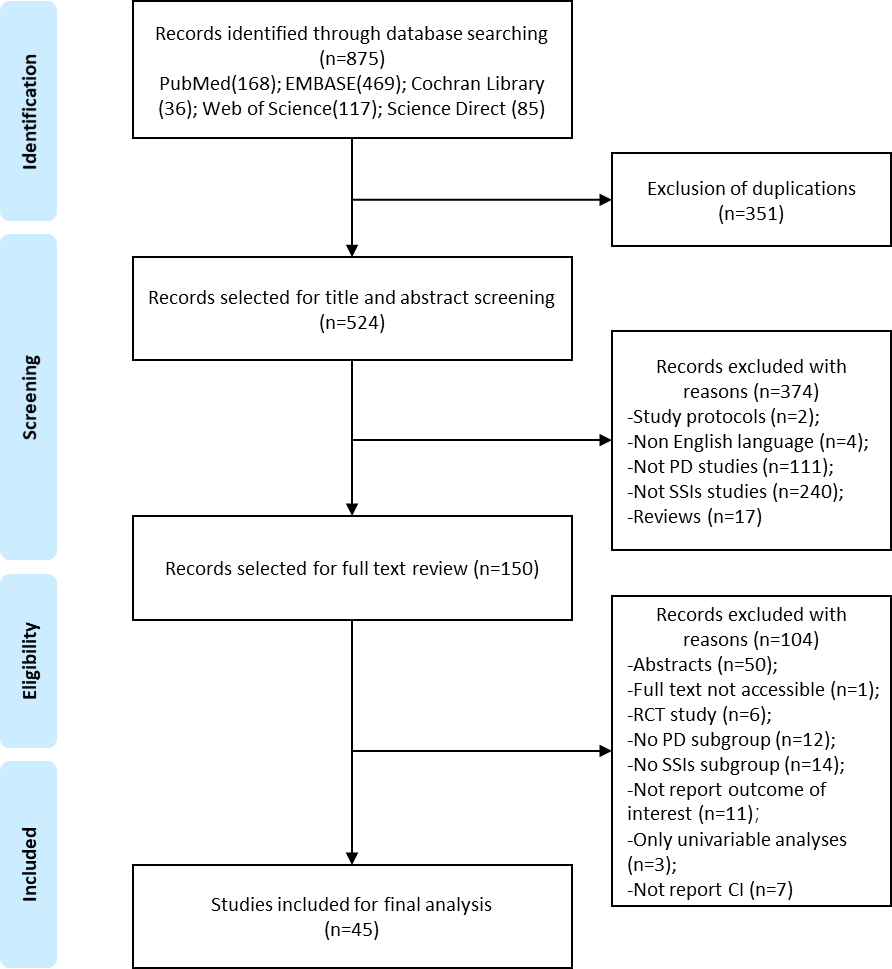 |
| --- |
| **Figure 1.** PIRSMA Flowchart of the study selection |
| **Abbreviation:** PD: Pancreaticoduodenectomy; SSIs: Surgical site infections; RCT: Randomized controlled trial; CI: Confidence interval |

# Results of prevalence of surgical site infections (SSIs)

### Subgroup analysis

#### Group variables by region

| **A. Total SSIs** |
| --- |
| 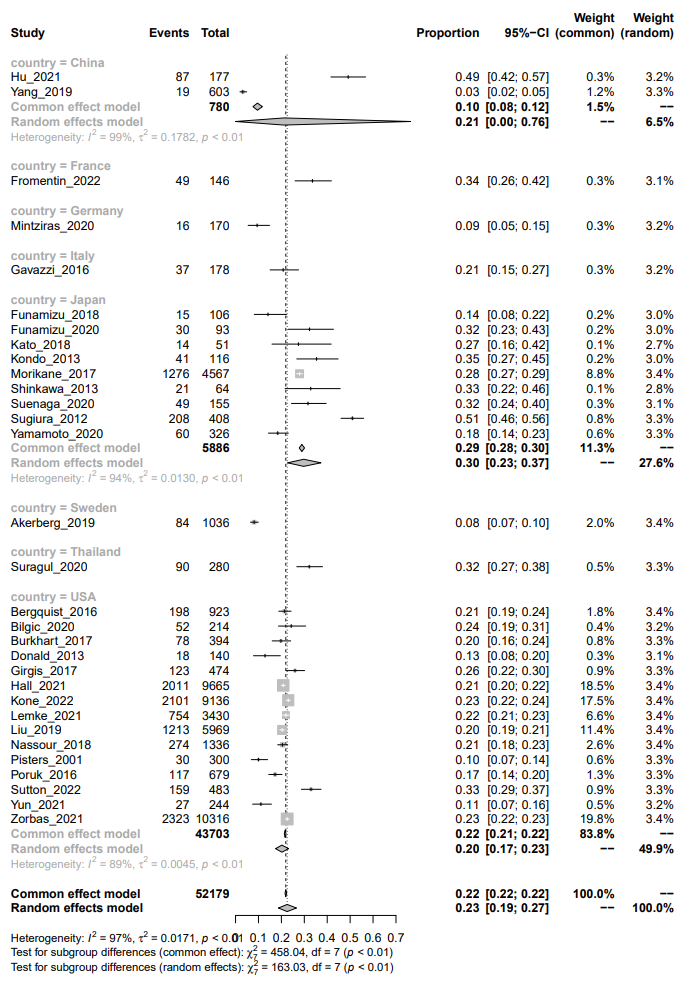 |
| **B. Superficial incisional SSIs** |
| 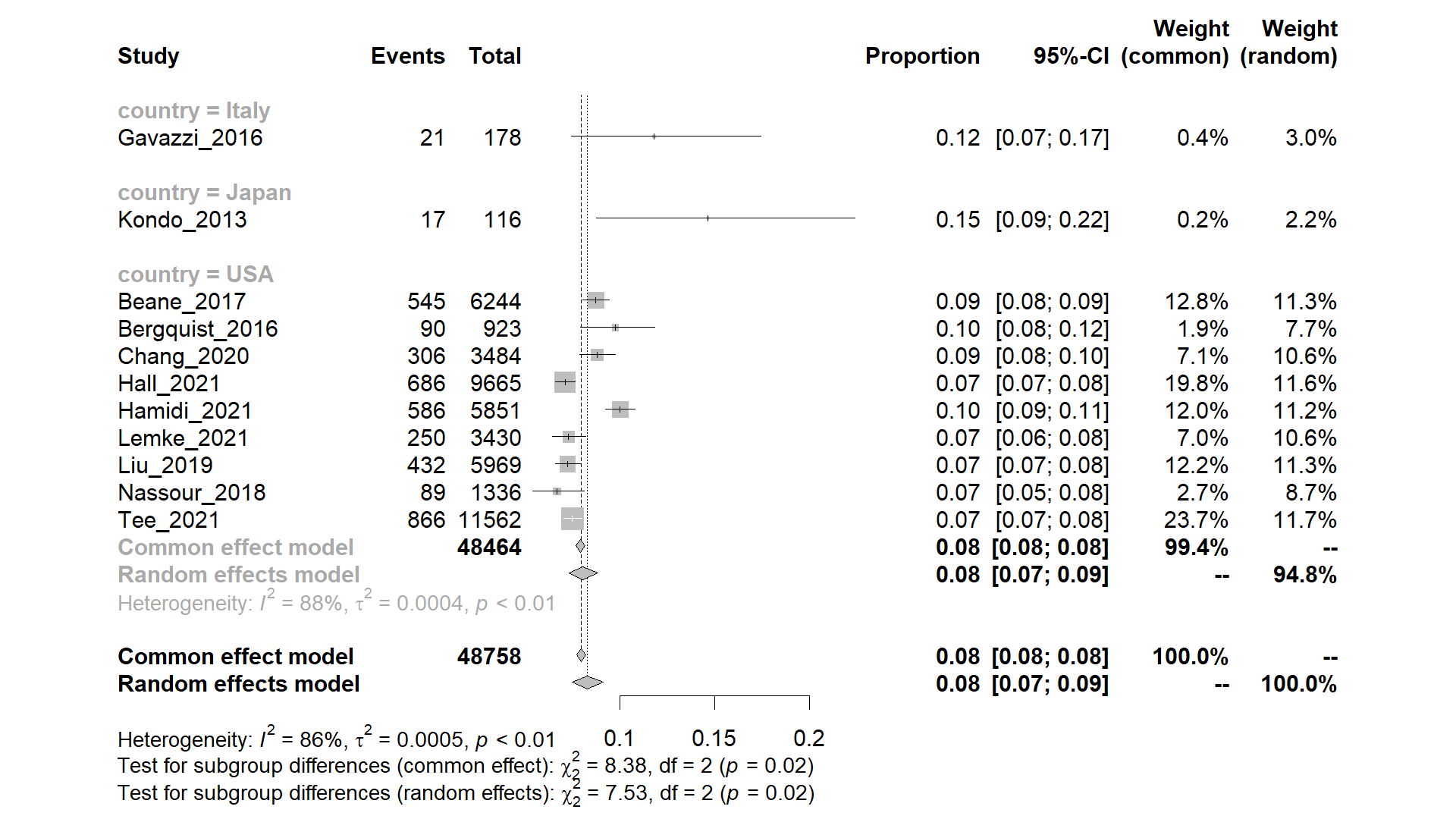 |
| **C. Deep incisional SSIs** |
| 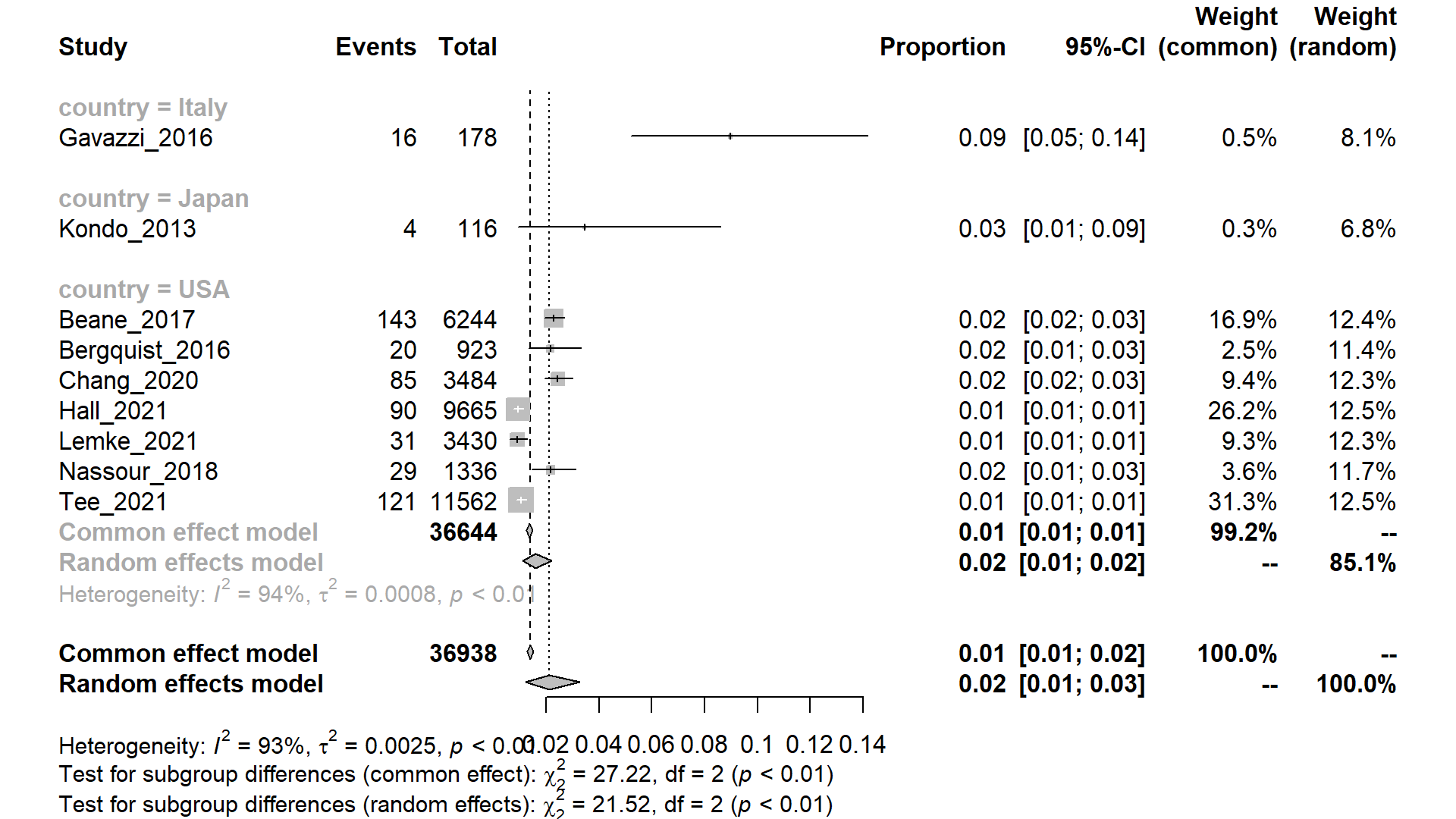 |
| **D. Organ/space SSIs** |
| 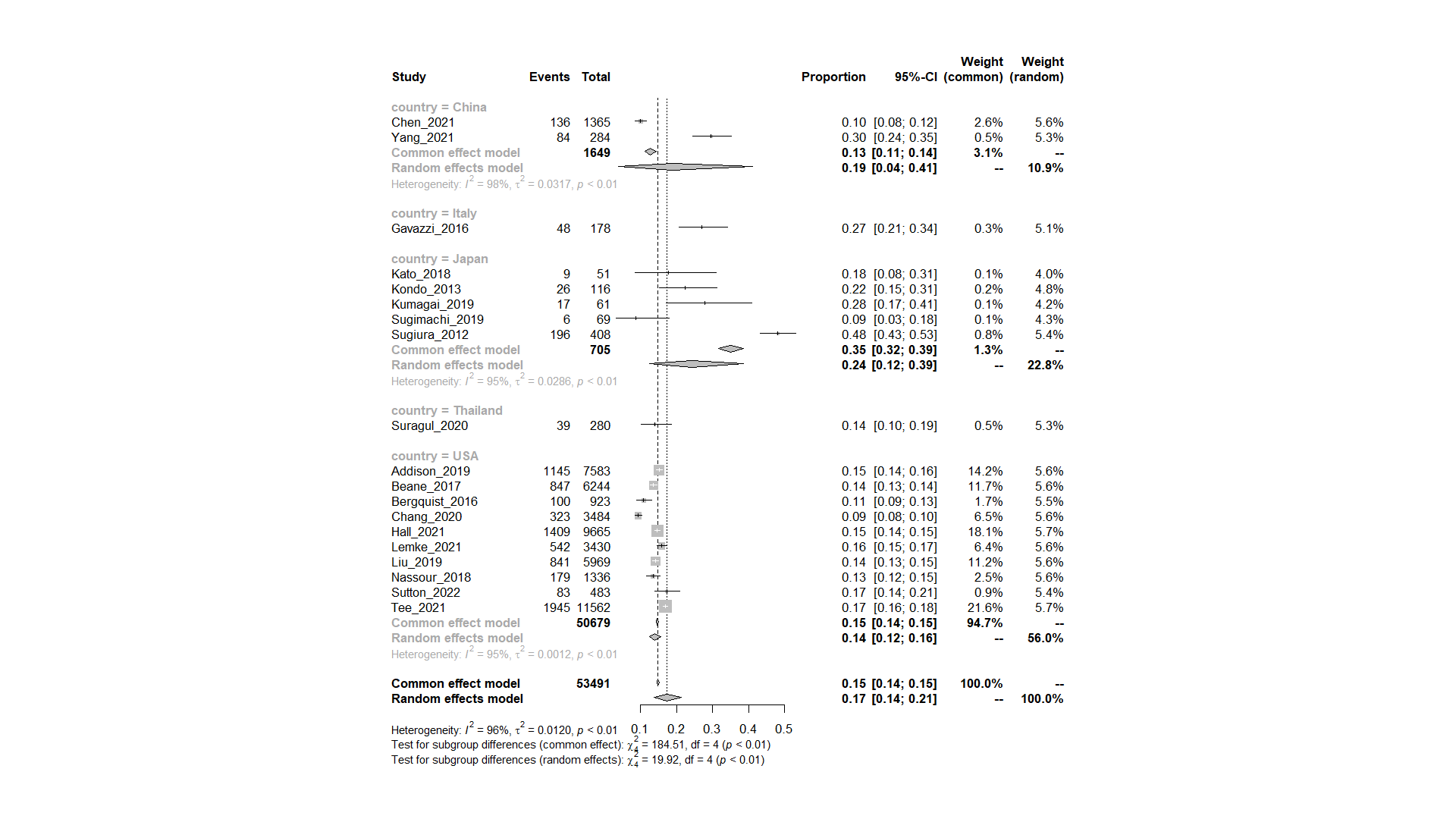 |
| **E. Superficial/Deep incisional SSIs** |
| 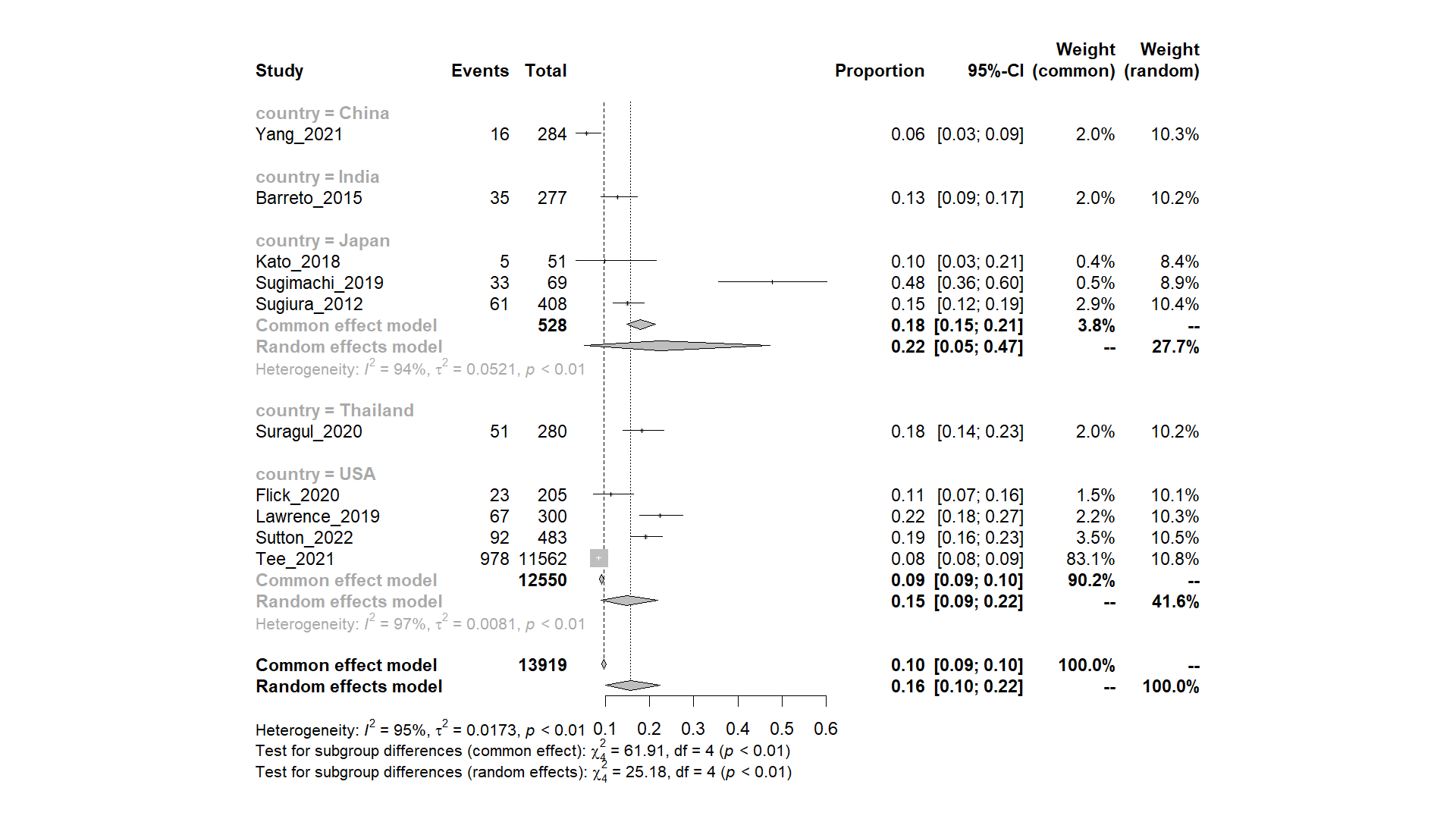 |
| **Figure 2.** Forest plots of prevalence of SSIs grouped by region |

#### Group variables by study design

| **A. Total SSIs** |
| --- |
| 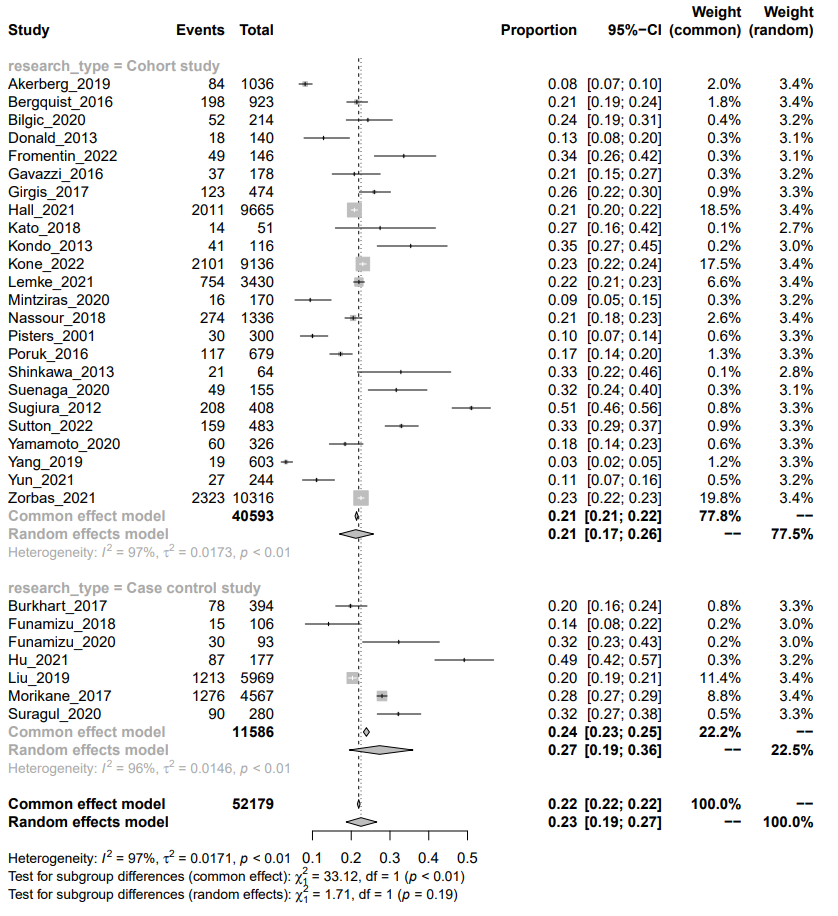 |
| **B. Superficial incisional SSIs** |
| 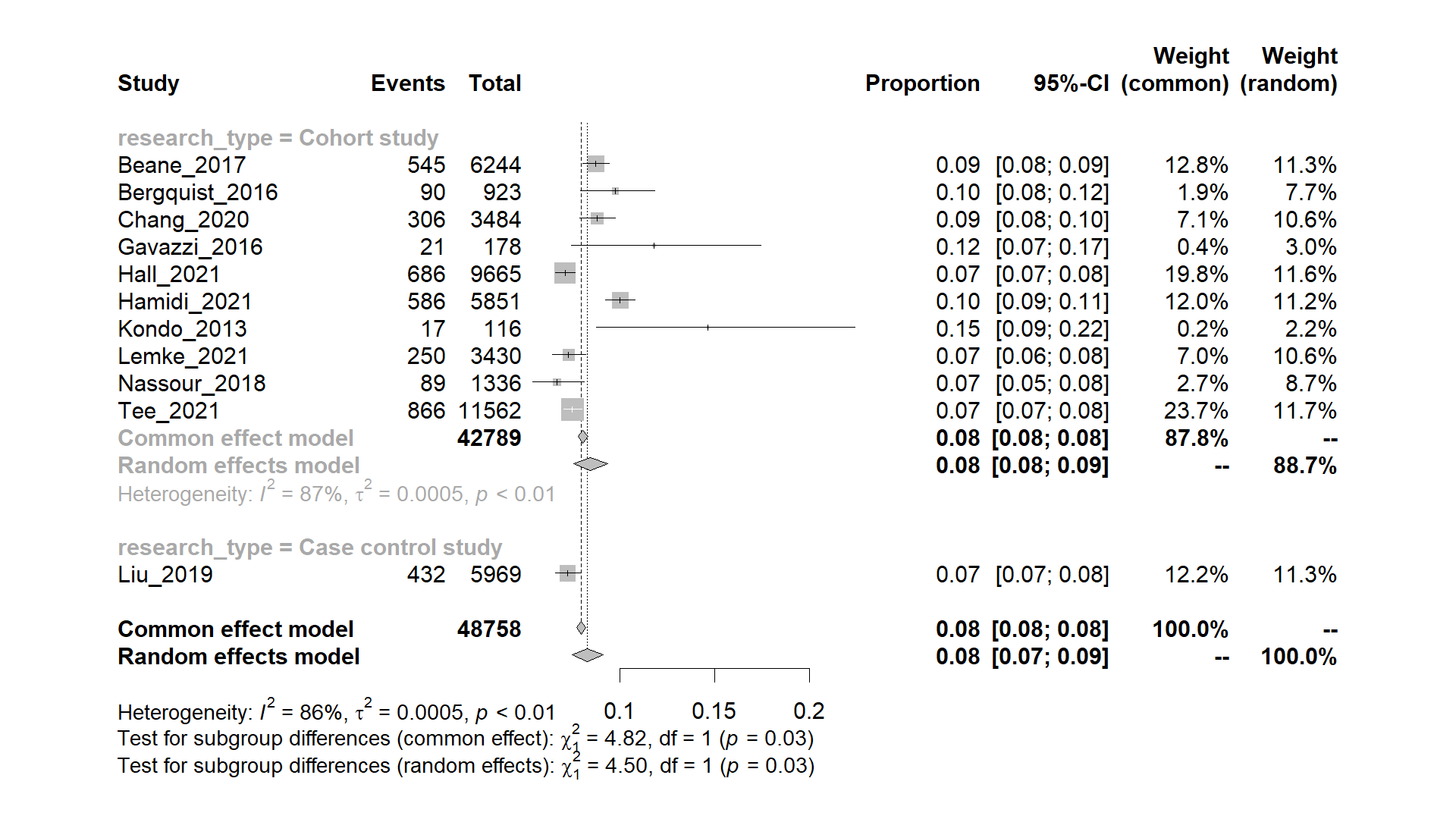 |
| **C. Organ/space SSIs** |
| 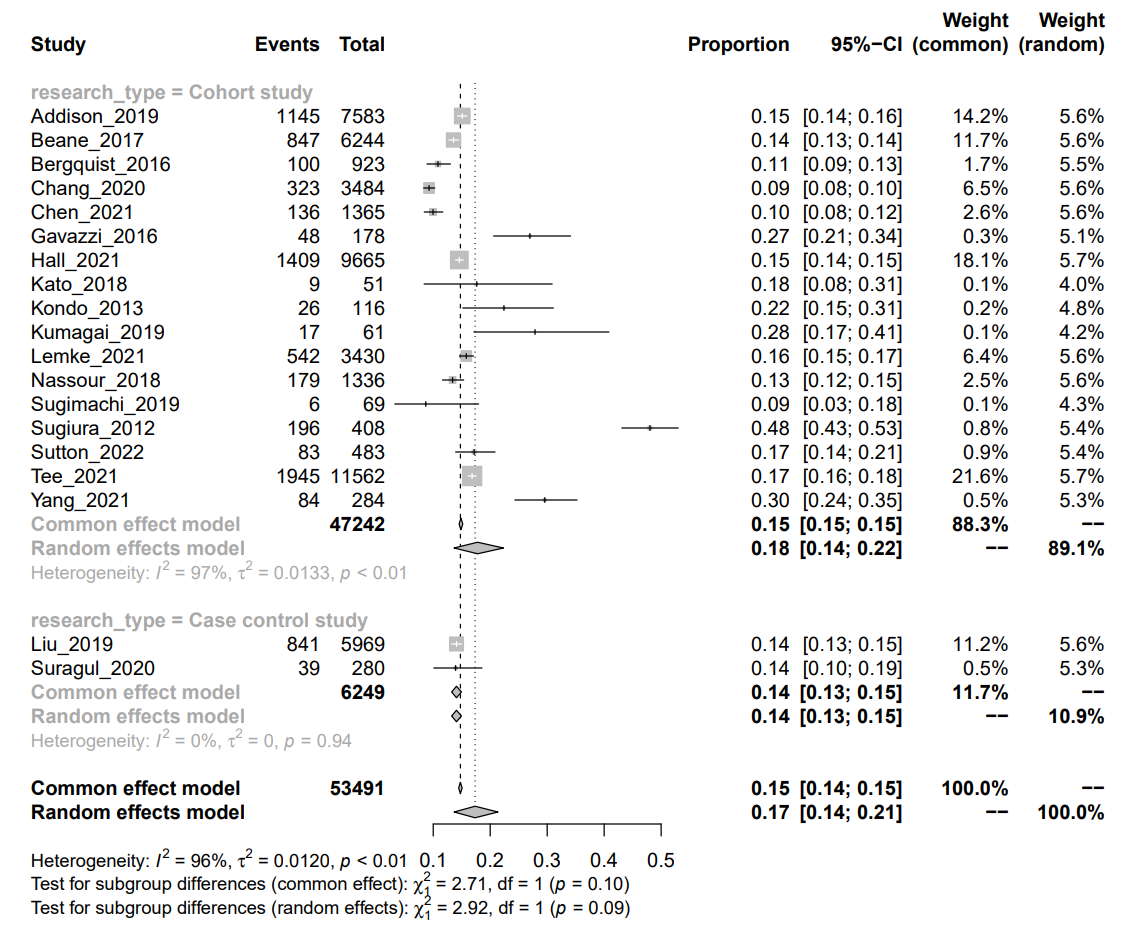 |
| **D. Superficial/Deep incisional SSIs** |
| 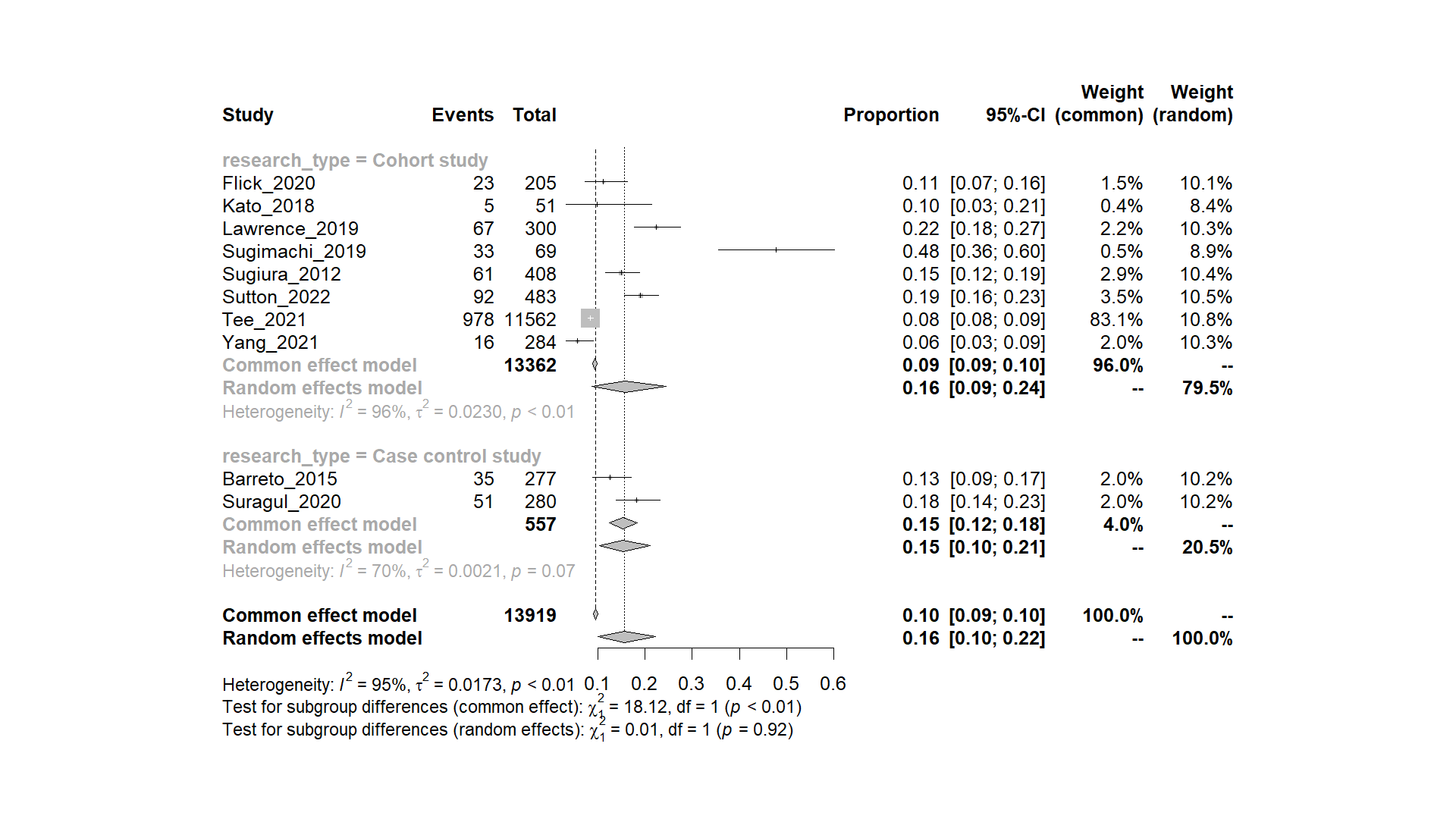 |
| **Figure 3.** Forest plots of prevalence of SSIs grouped by study design |
| *The studies on Deep incisional SSIs analysis are all cohort study. |

### Sensitivity analysis

| **Random-effect model** | **Common-effect model** |
| --- | --- |
| **A. Total SSIs** | |
| 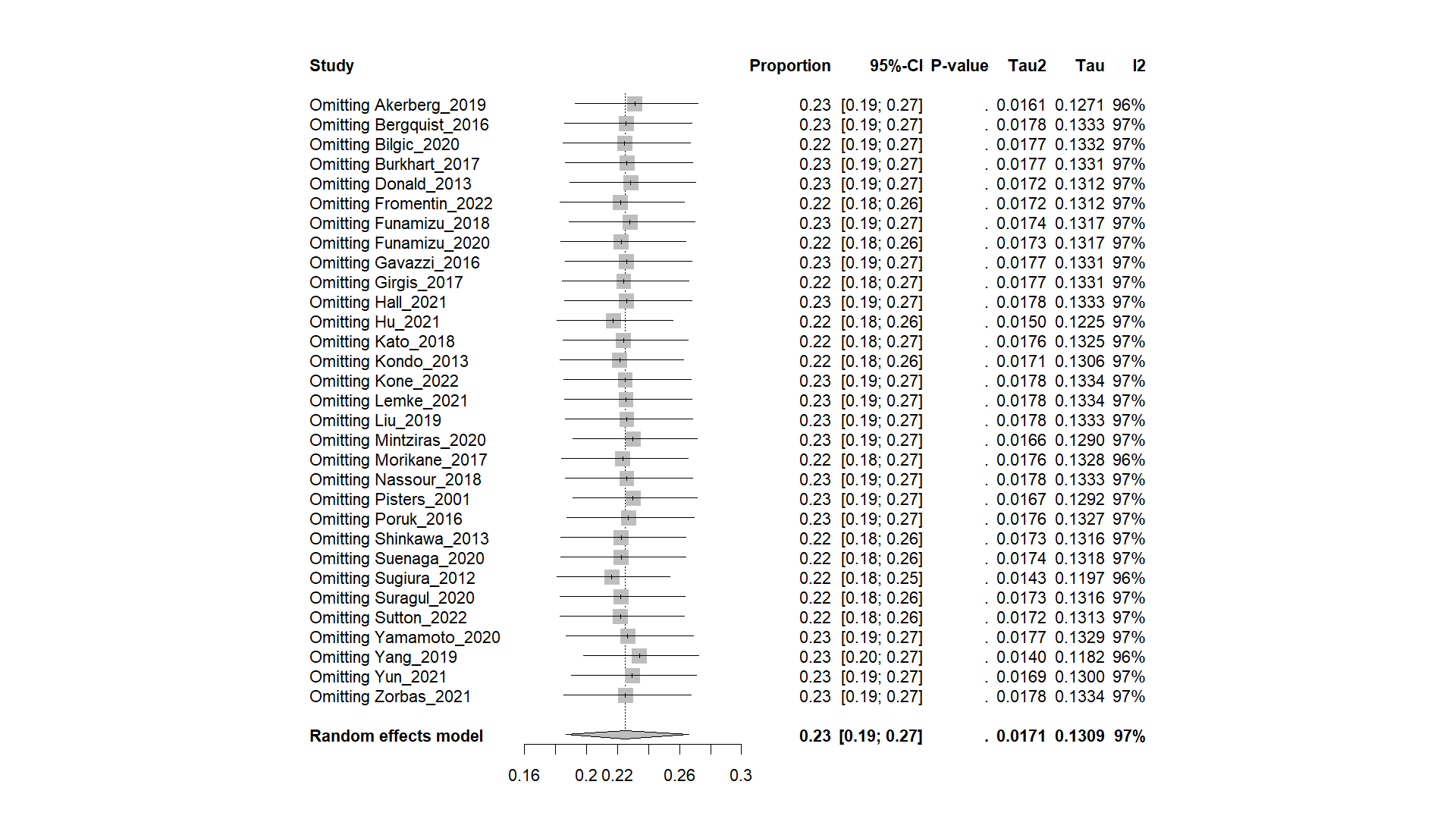 | 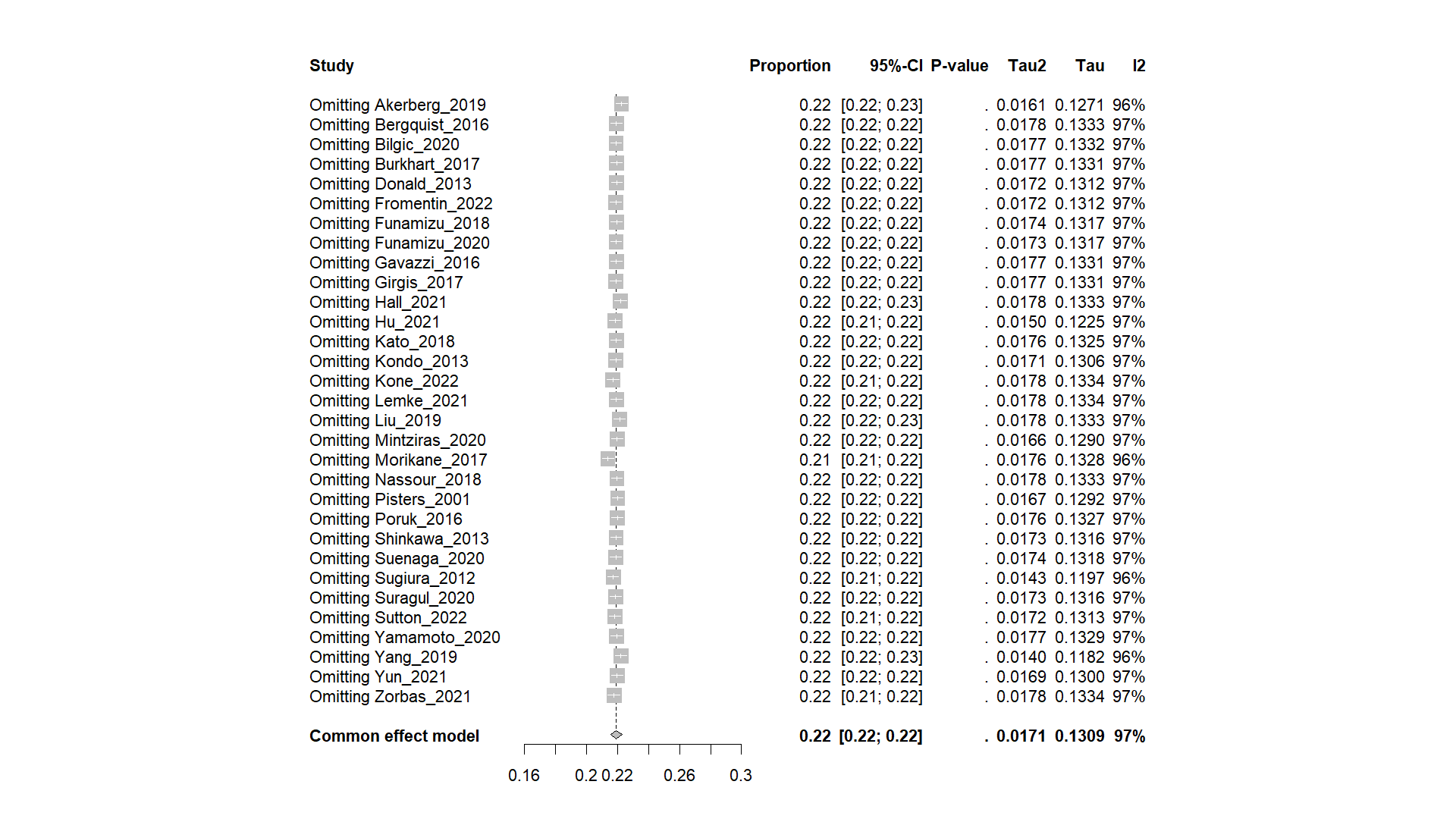 |
| **B. Superficial incisional SSIs** | |
| 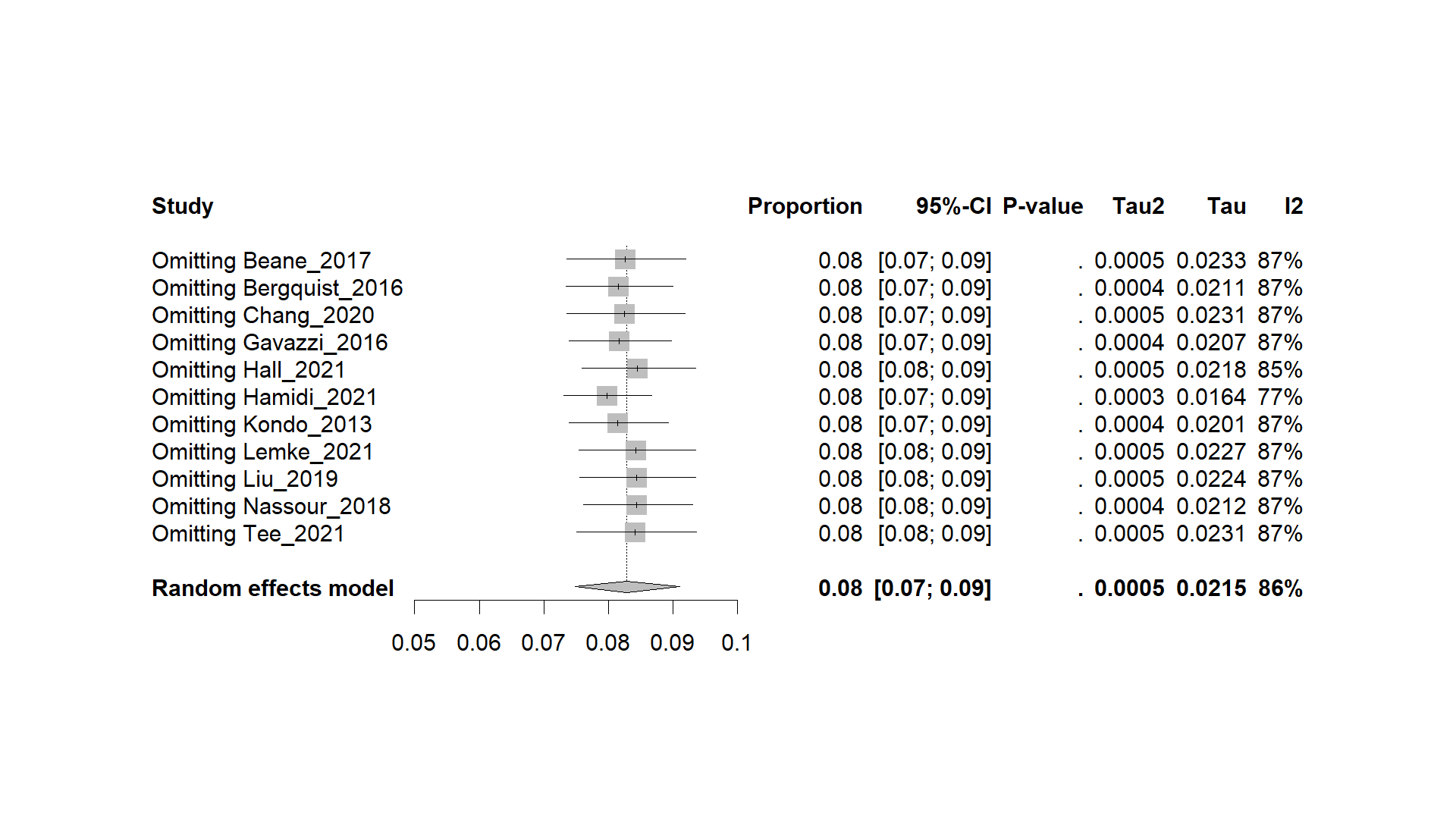 | 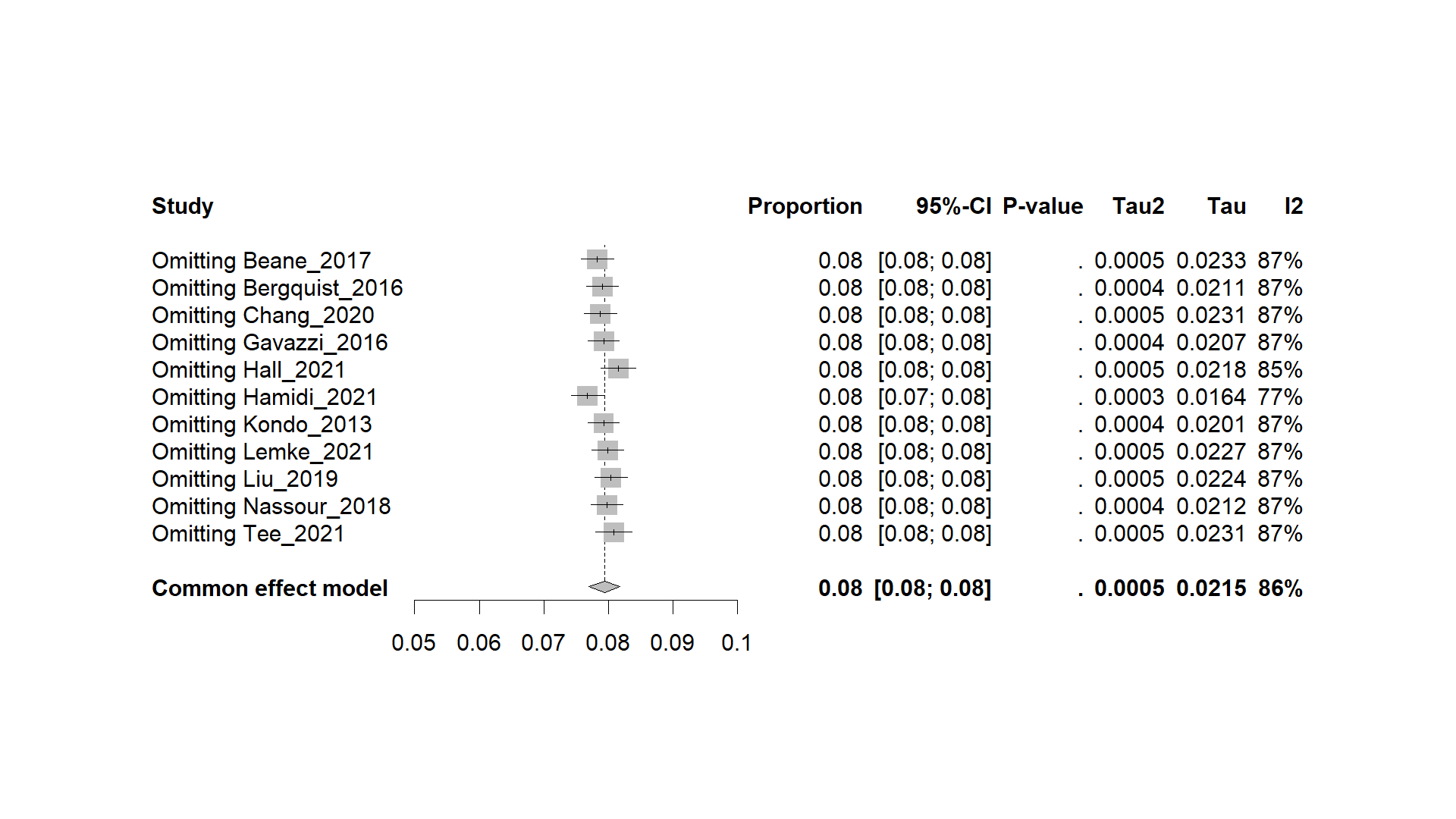 |
| **C. Deep incisional SSIs** | |
| 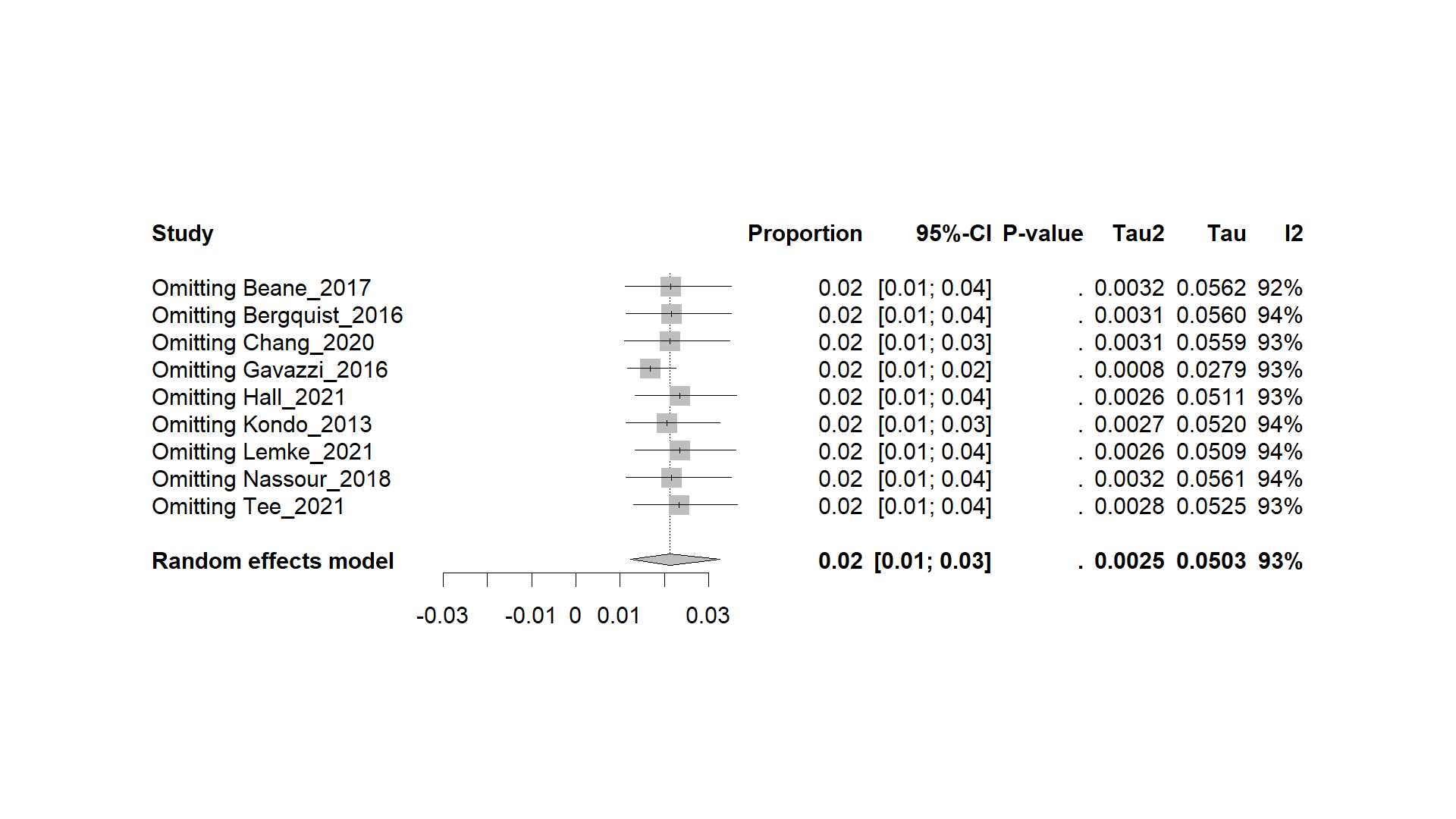 | 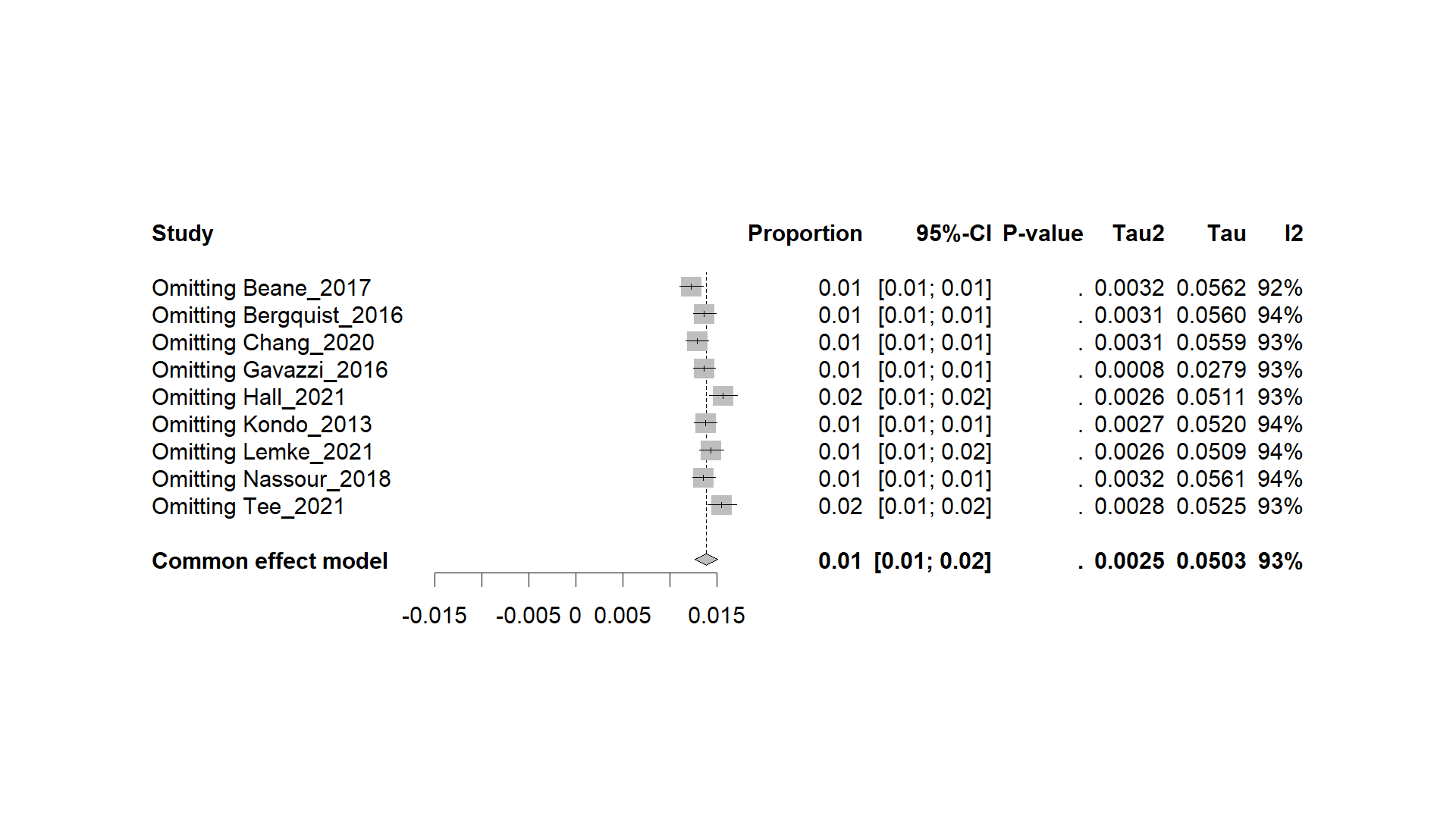 |
| **D. Organ/space SSIs** | |
| 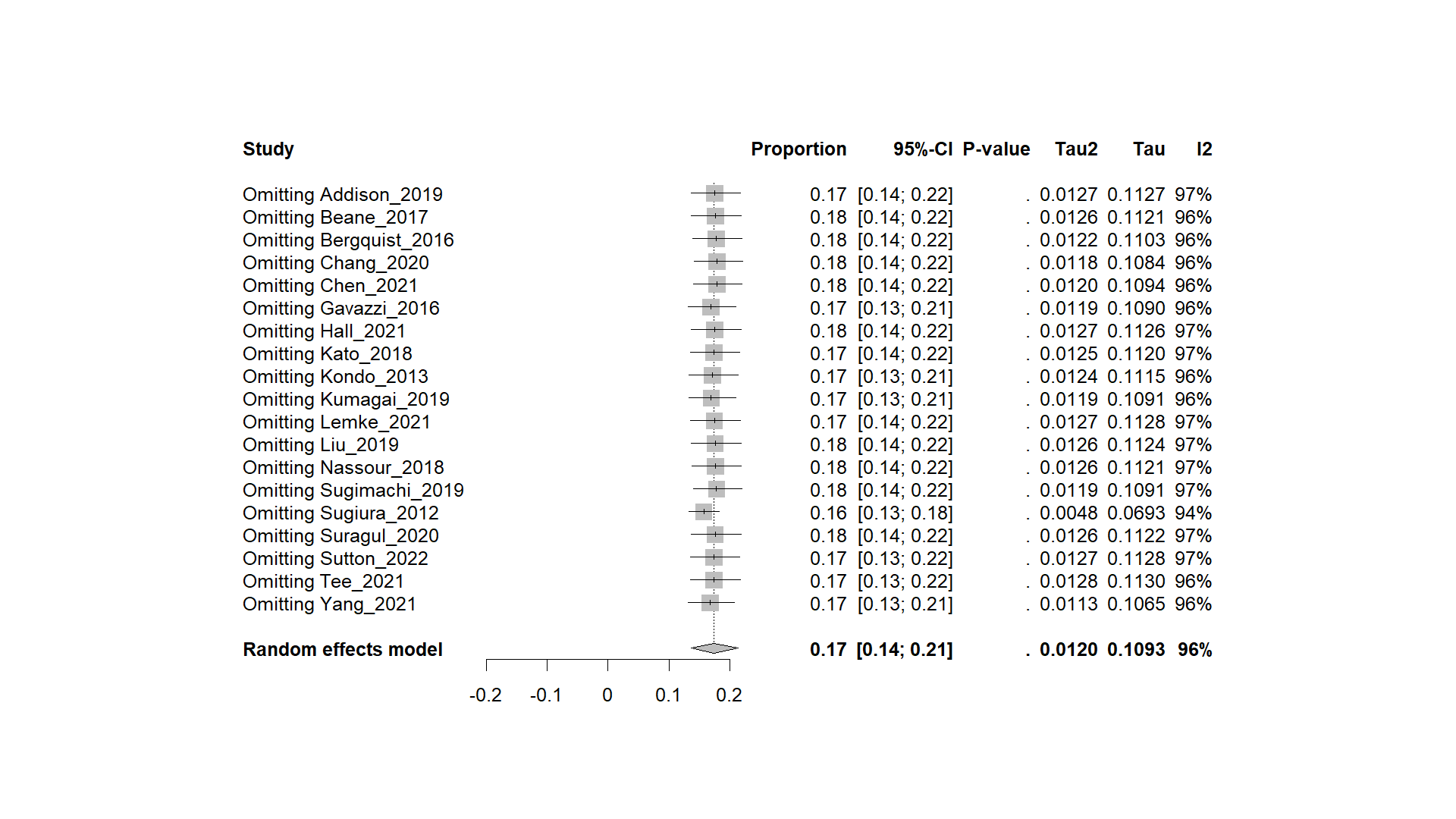 | 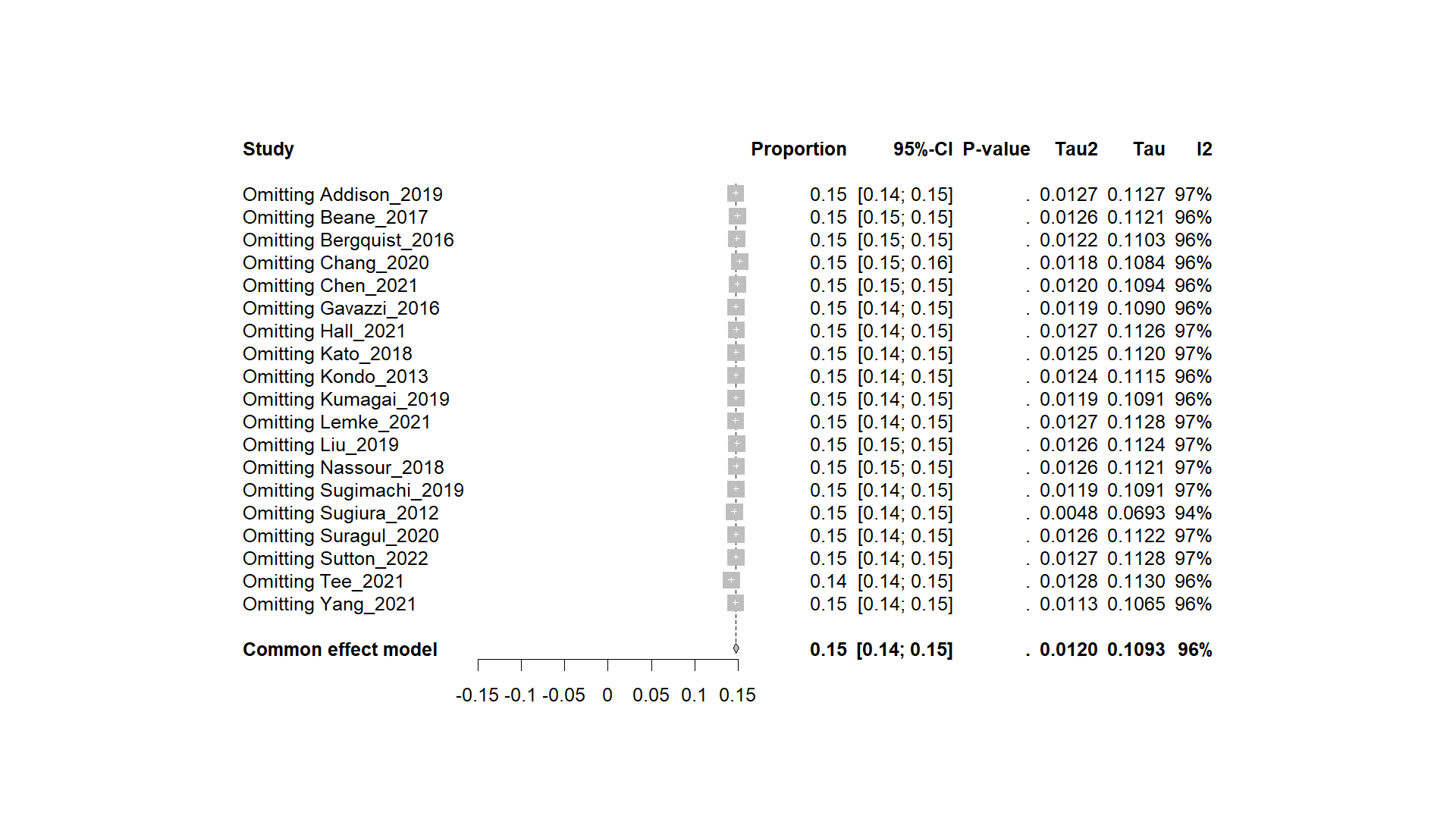 |
| **E. Superficial/Deep incisional SSIs** |  |
| 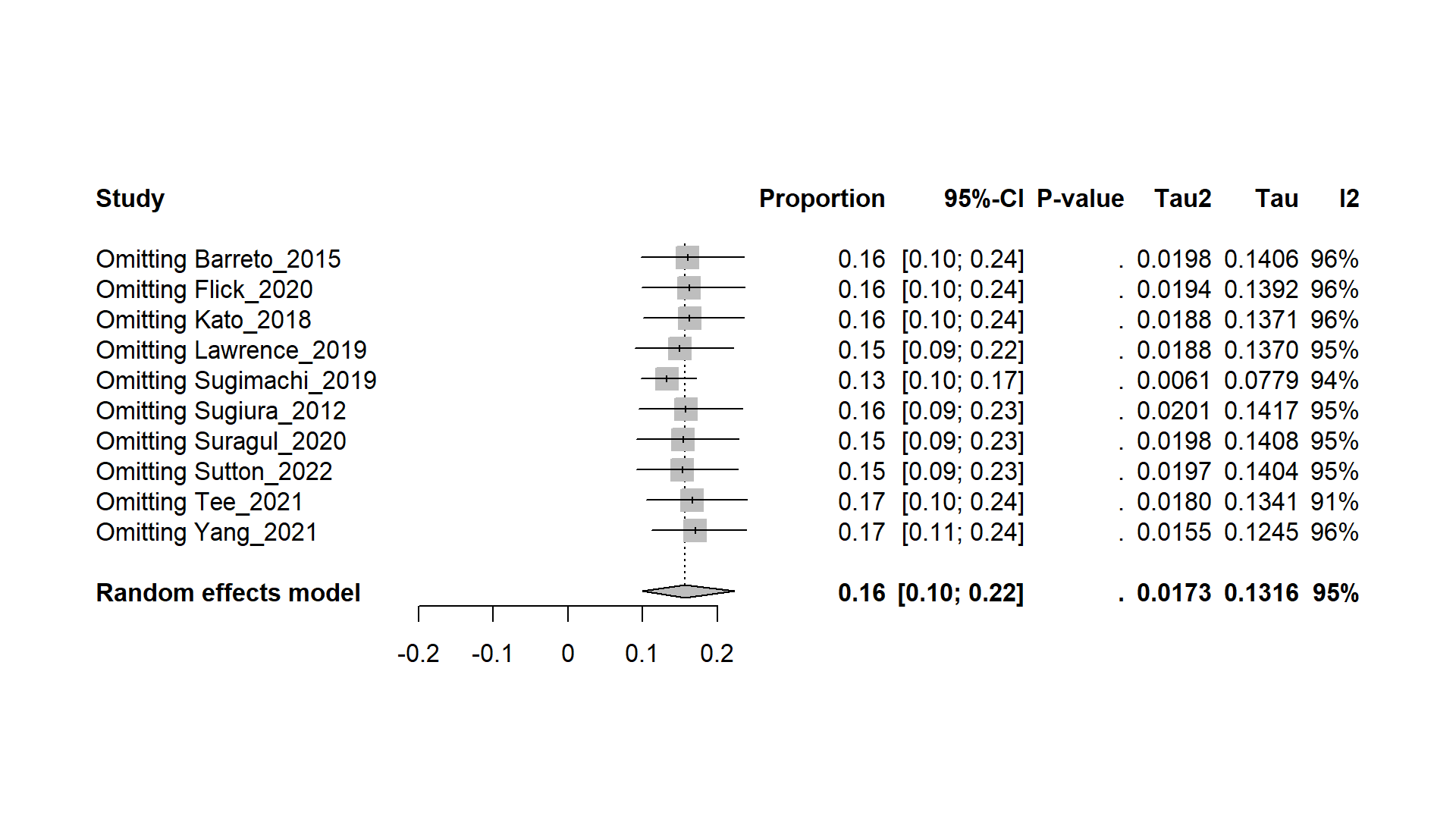 | 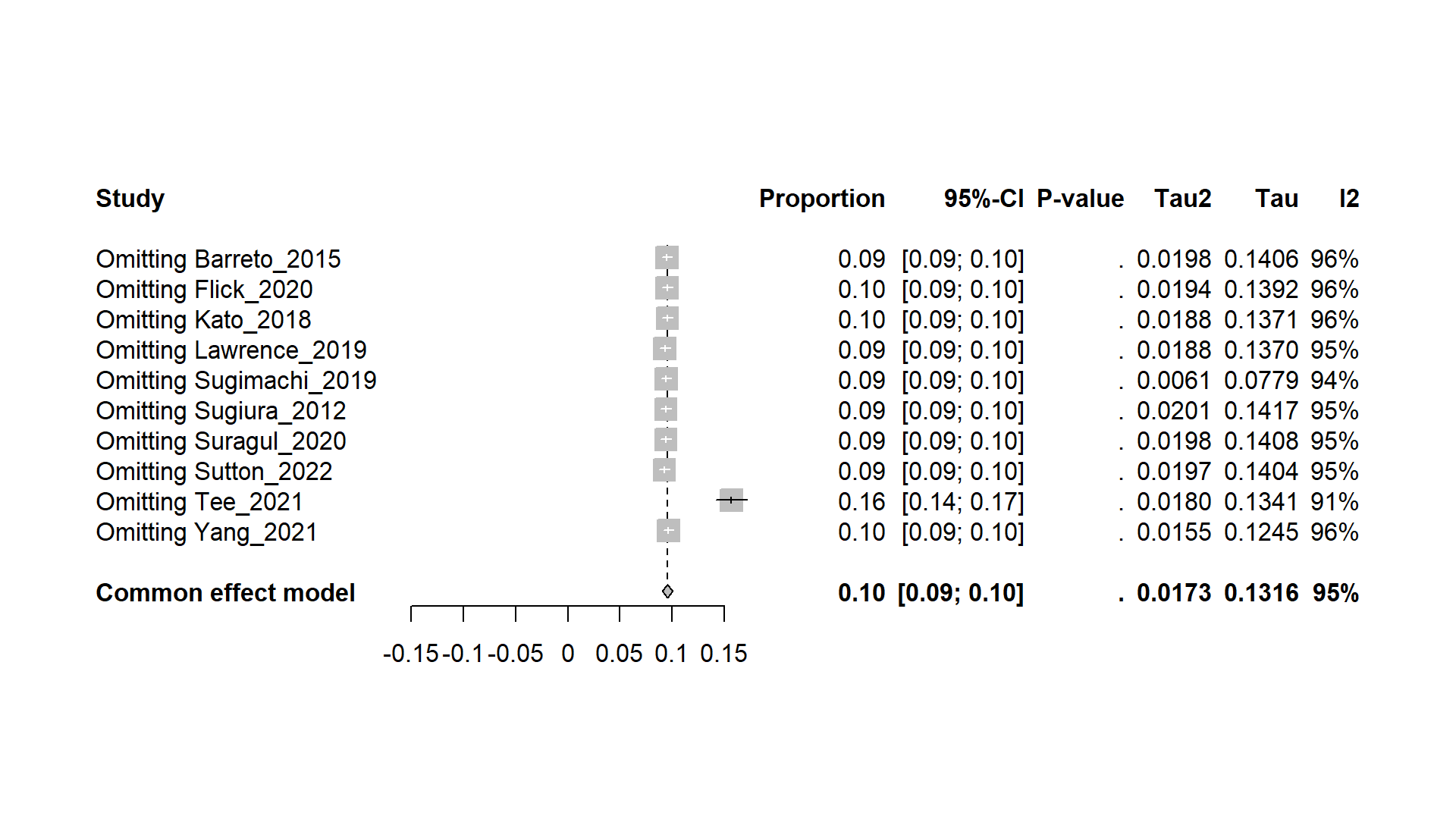 |
| **Figure 4.** Sensitivity analysis graphs of prevalence of SSIs | |

### Publication bias assessment

| **Funnel plot** | **Egger’s regression test graph** | **Trim and fill graph** |
| --- | --- | --- |
| **A. Total SSIs** | | |
| 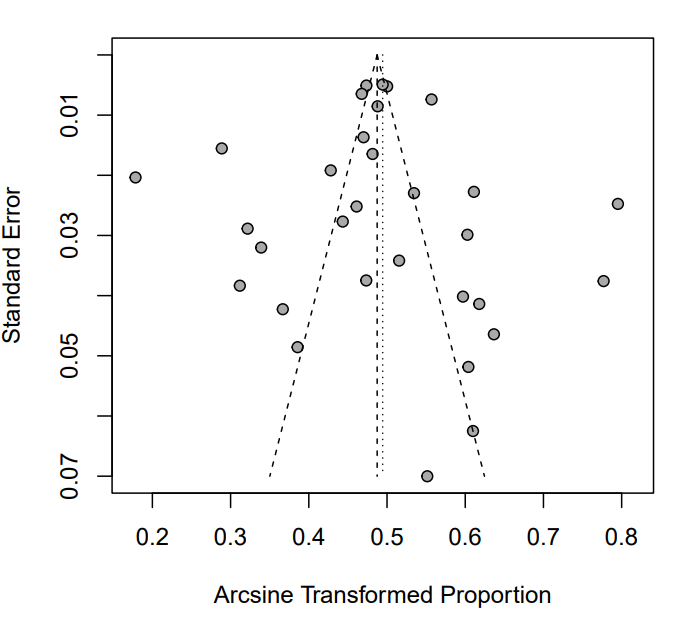 | 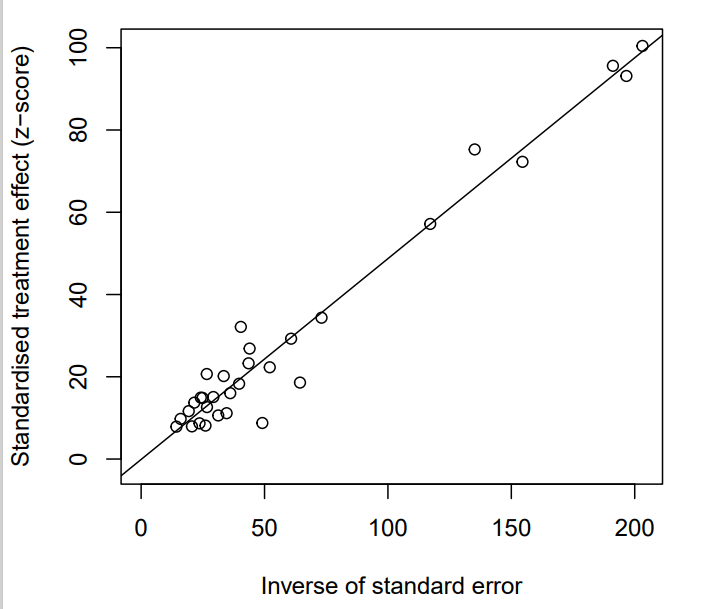 | 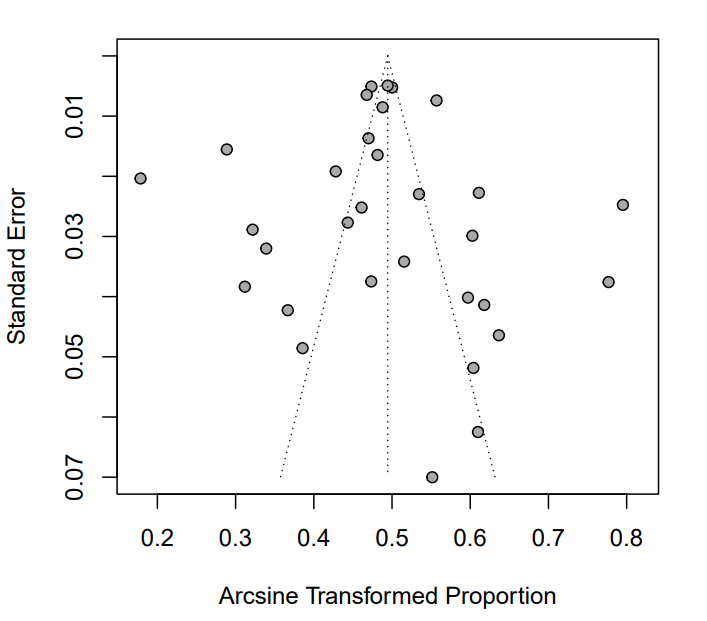 |
| **B. Superficial incisional SSIs** | | |
| 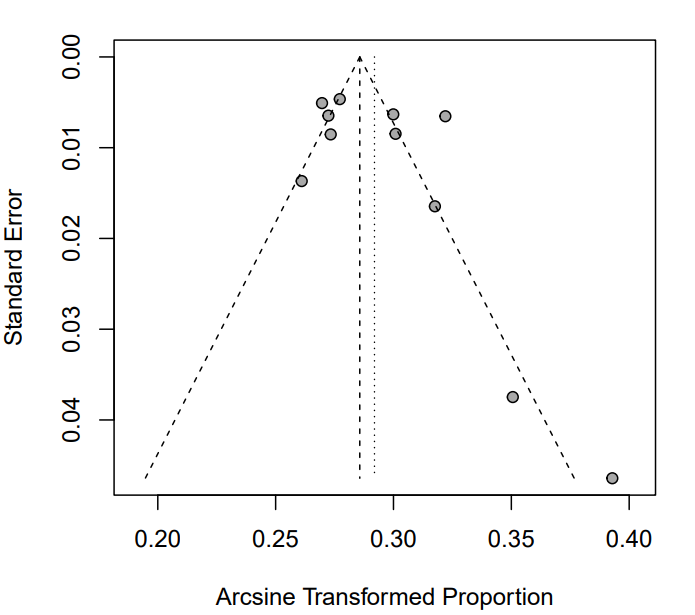 | 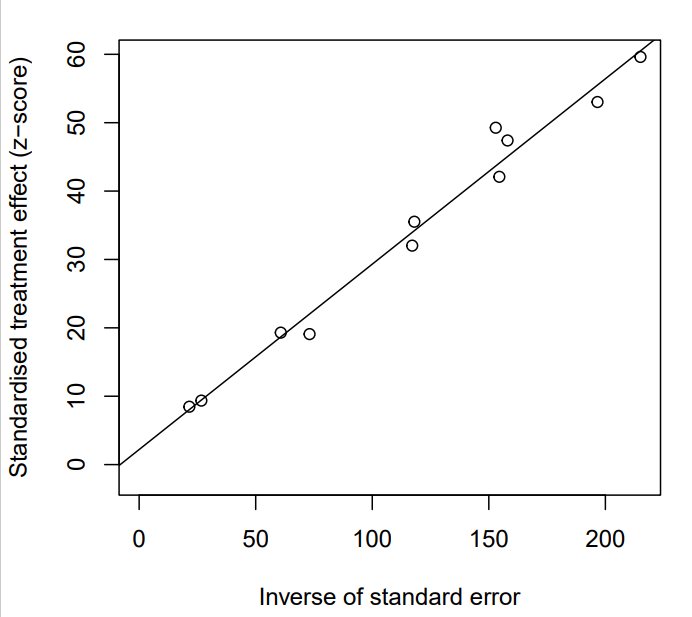 | 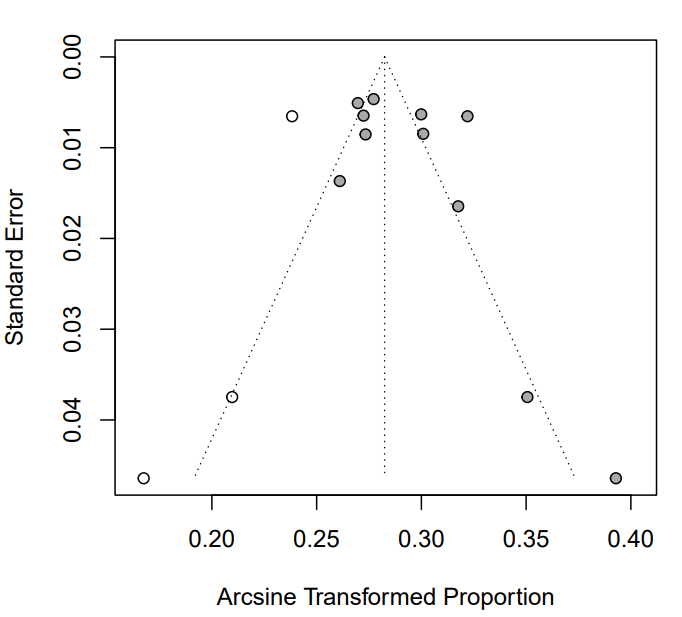 |
| **C. Deep incisional SSIs** | | |
| 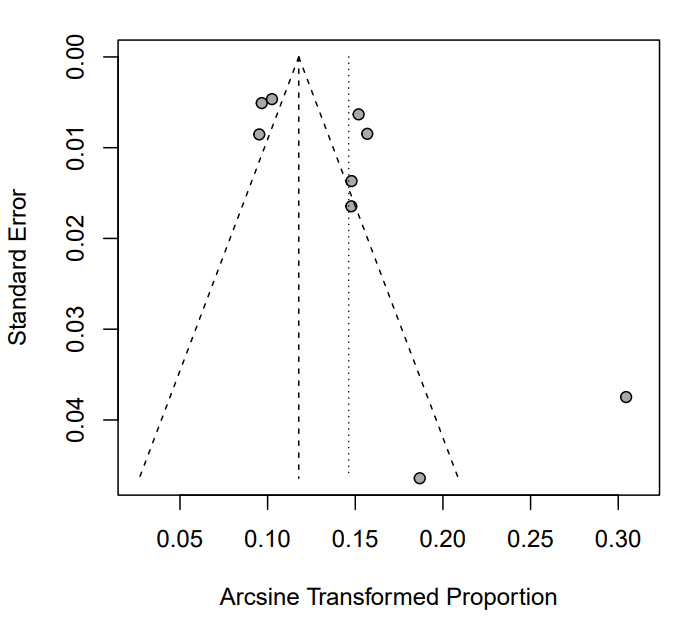 | 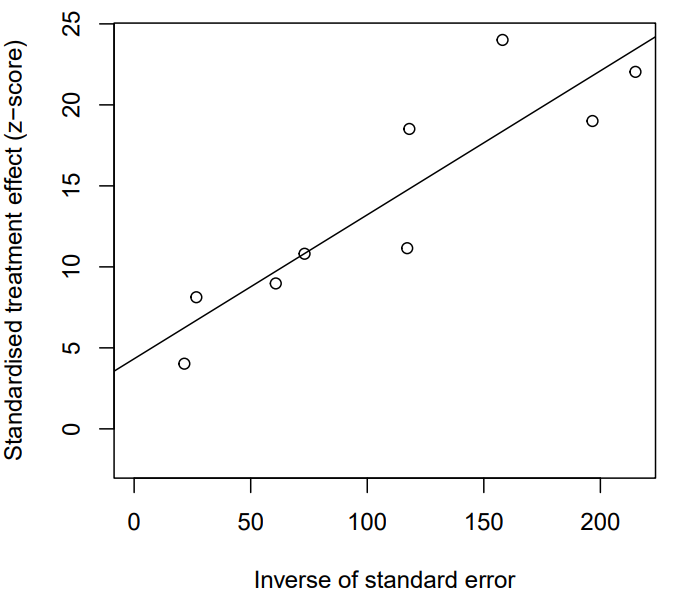 | 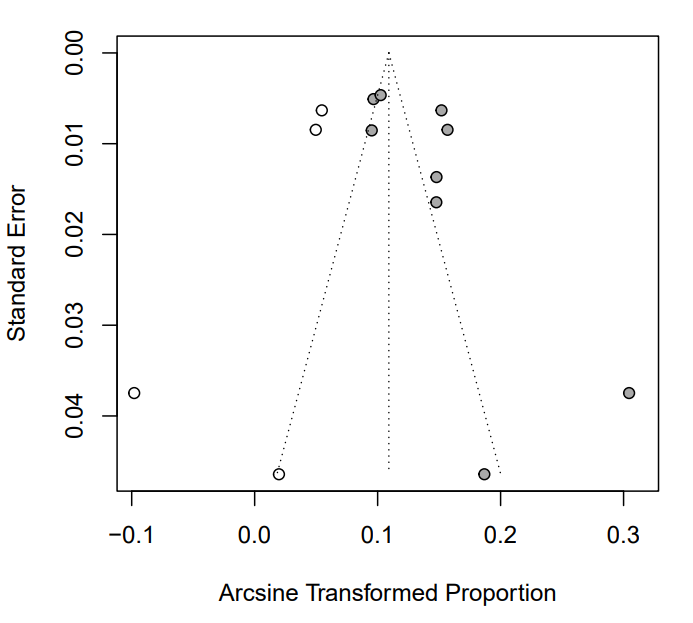 |
| **D. Organ/space SSIs** | | |
| 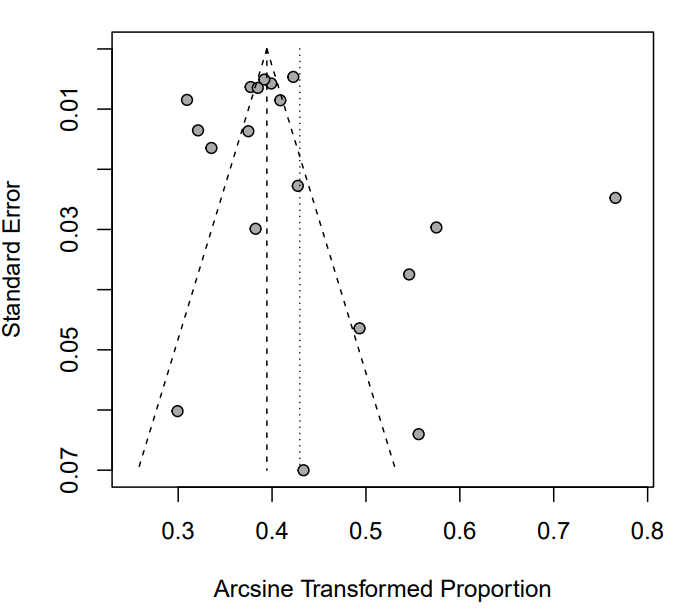 | 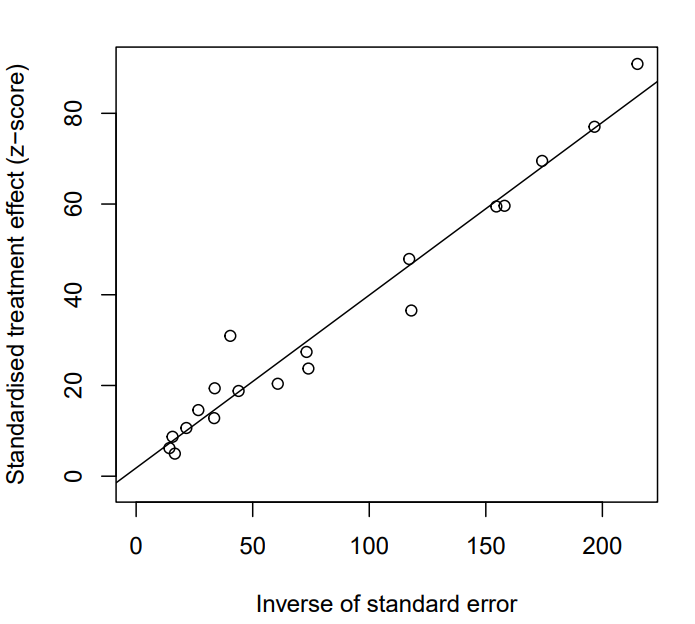 | 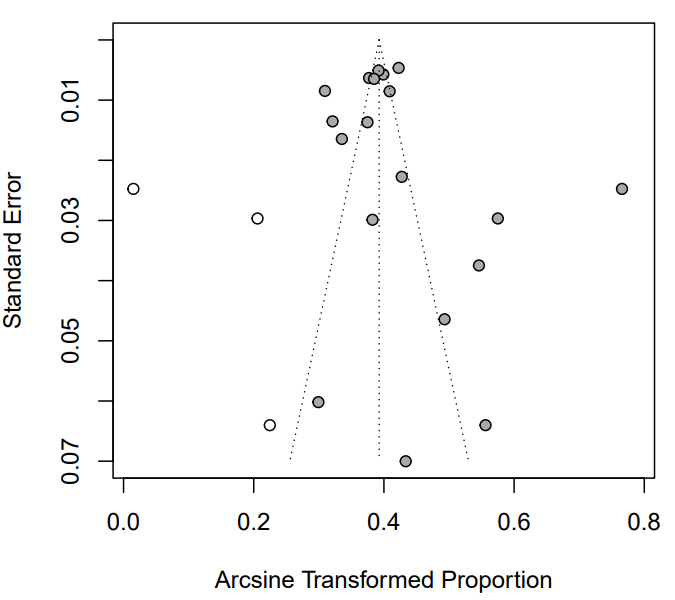 |
| **E. Superficial/Deep incisional SSIs** | | |
| 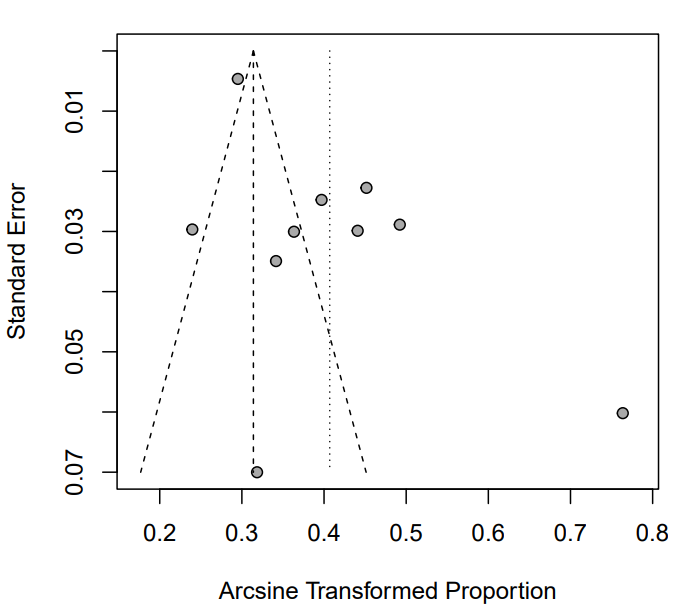 | 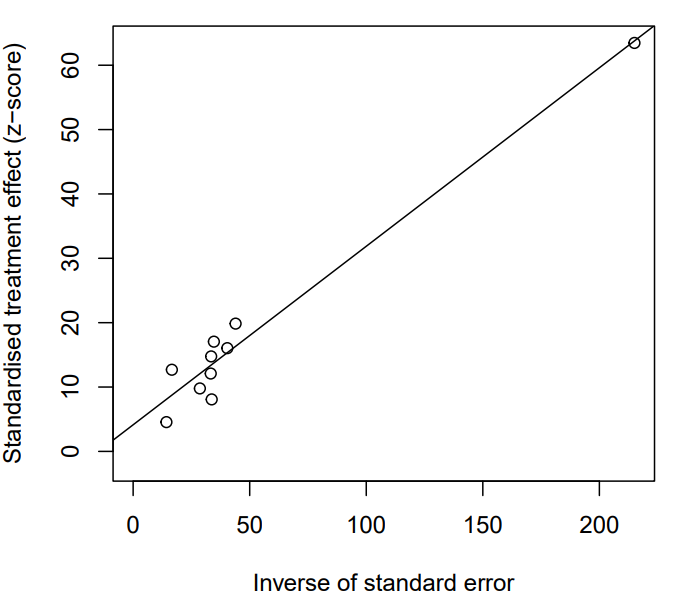 | 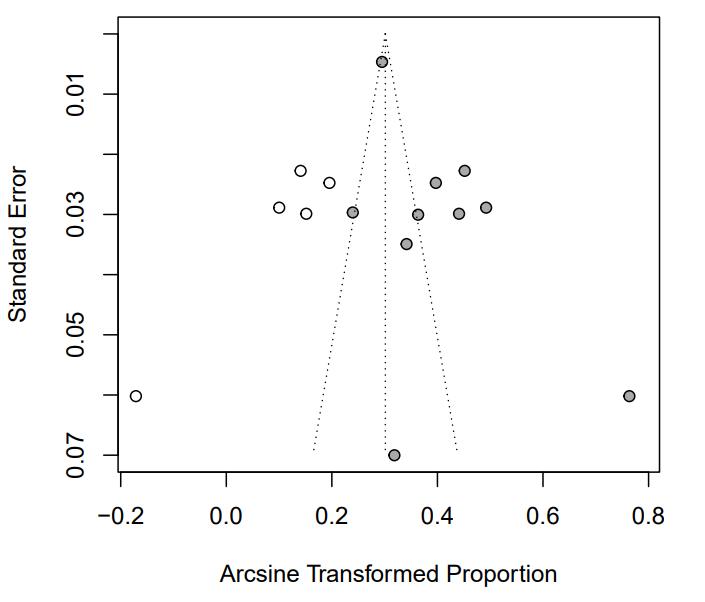 |
| **Figure 5.** Publication bias assessment graphs of prevalence of SSIs | | |

# Results of risk factors of SSIs

## Subgroup analysis

### Group variables by region

| **A. Preoperative biliary stenting** |
| --- |
| 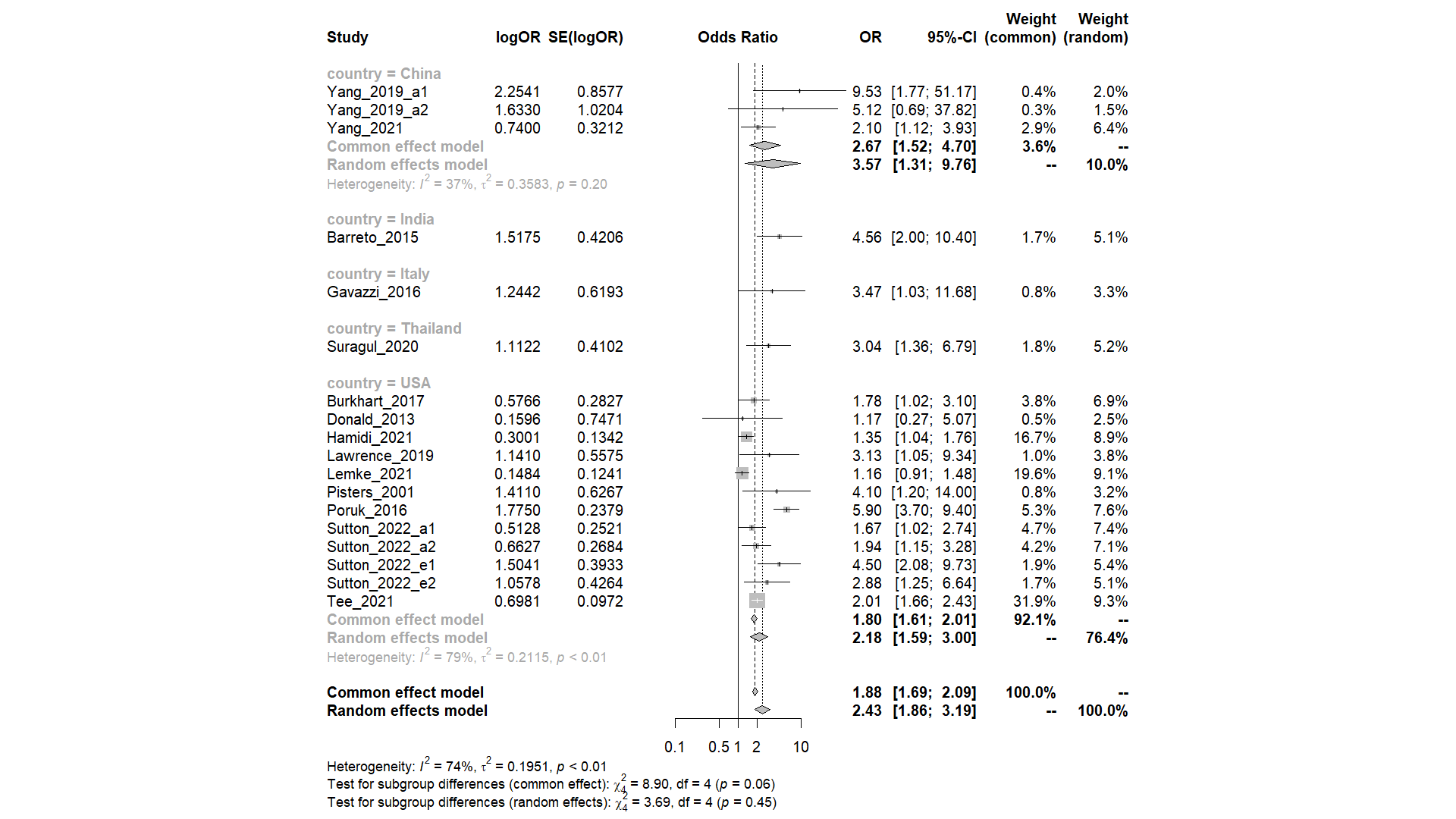 |
| **B. Body mass index (High vs Low)** |
| 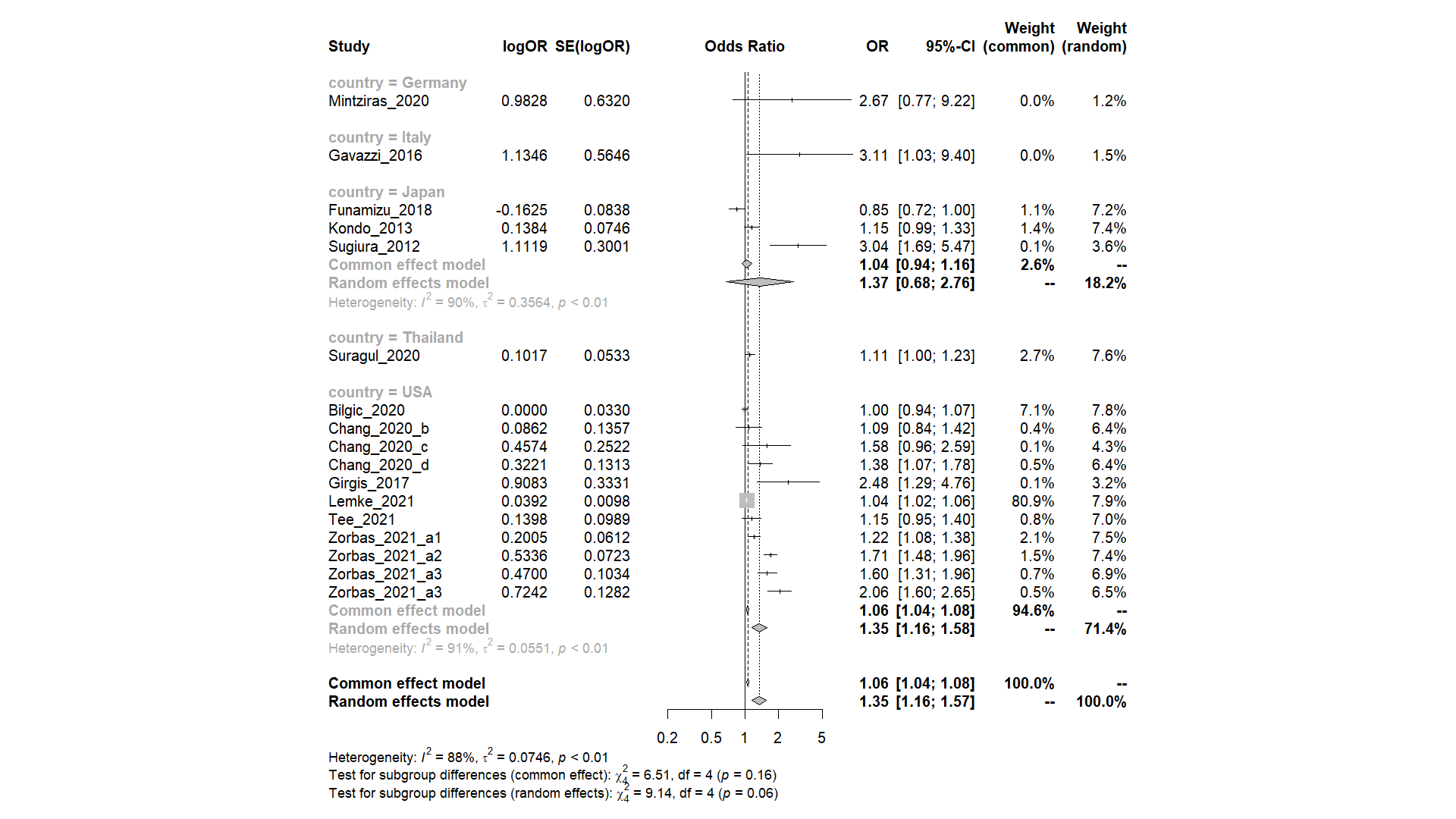 |
| **C. Operation time (Long vs Short)** |
| 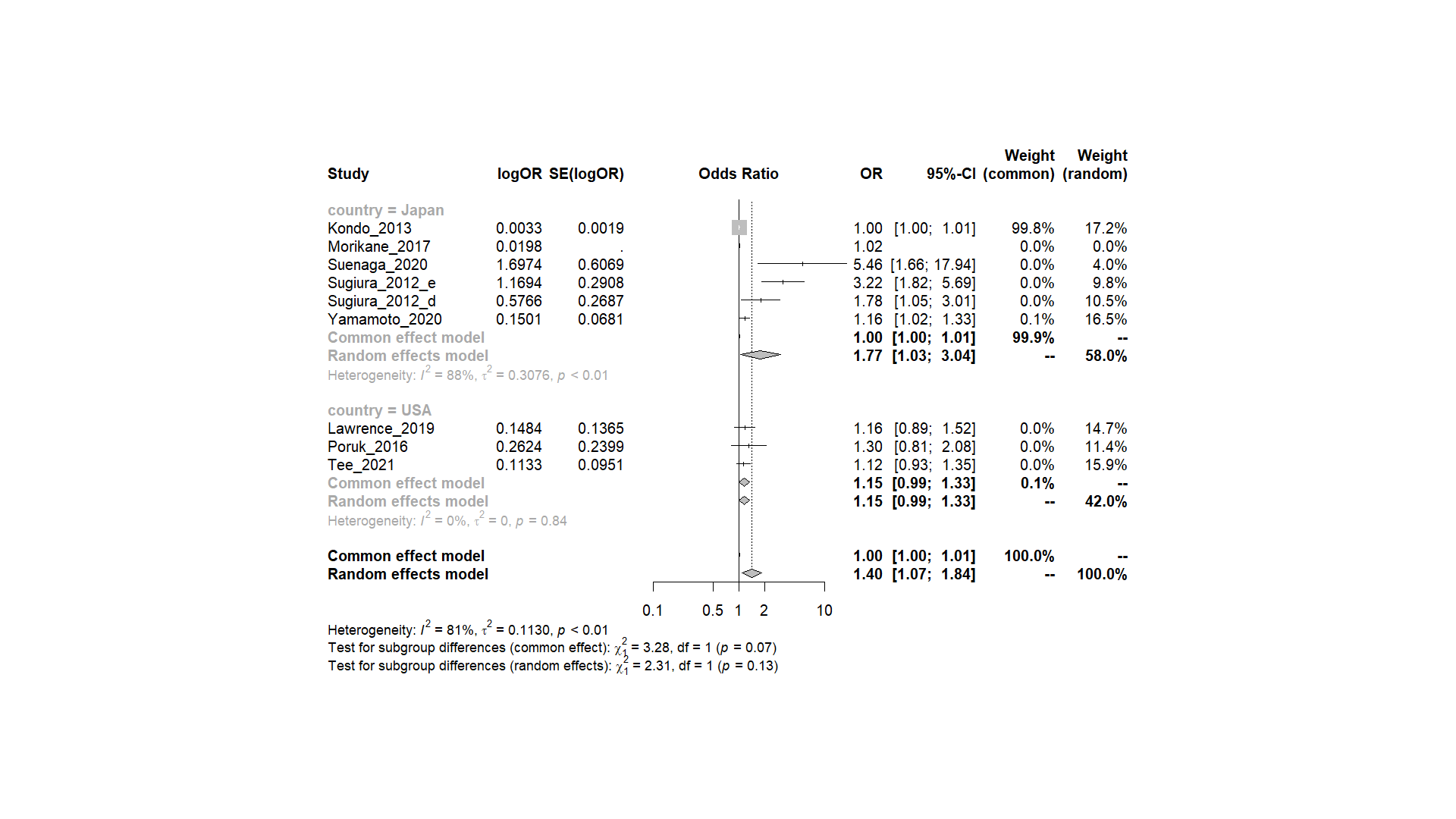 |
| **D. Sex (Male vs Female)** |
| 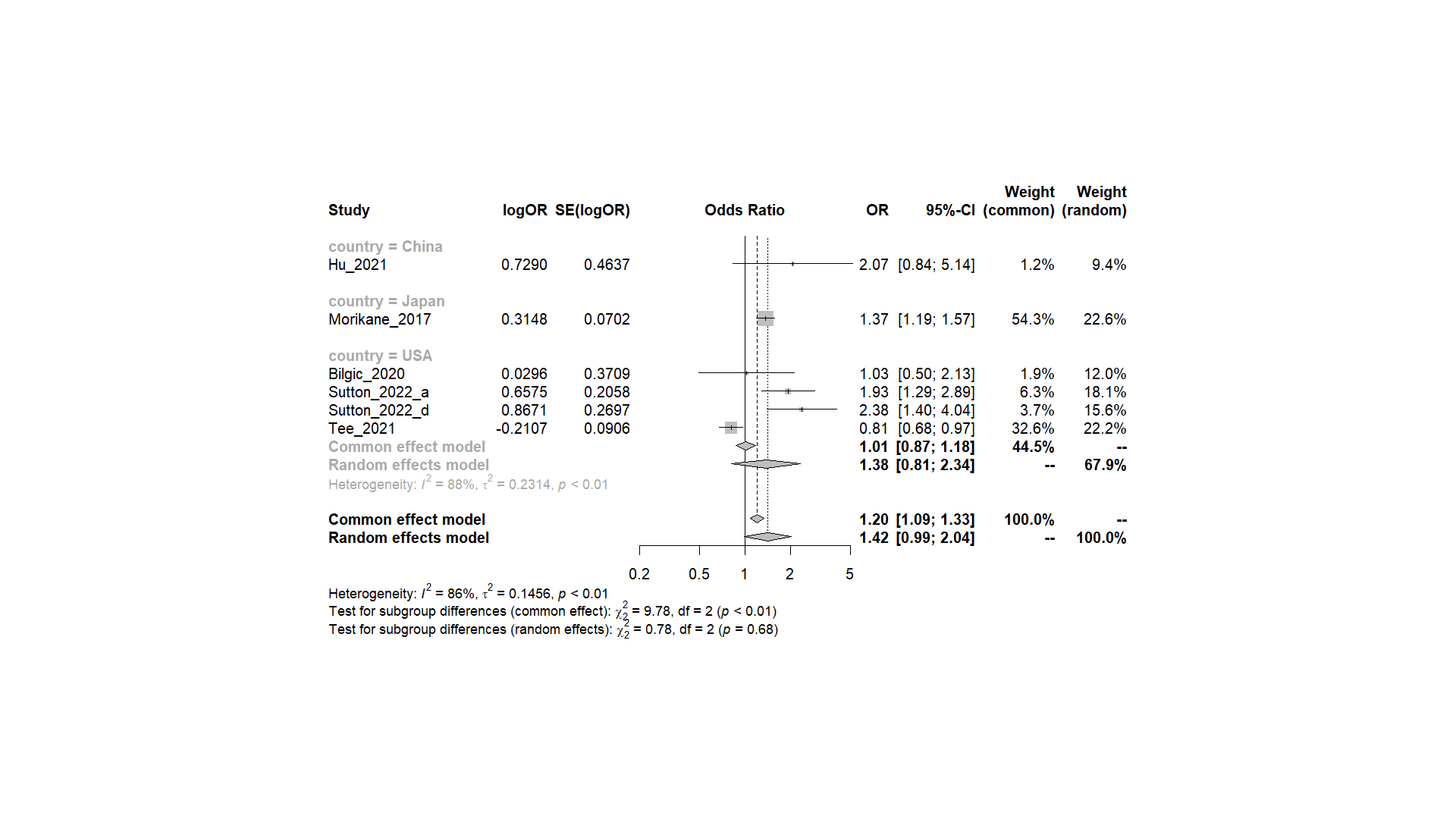 |
| **E. Postoperative pancreatic fistula** |
| 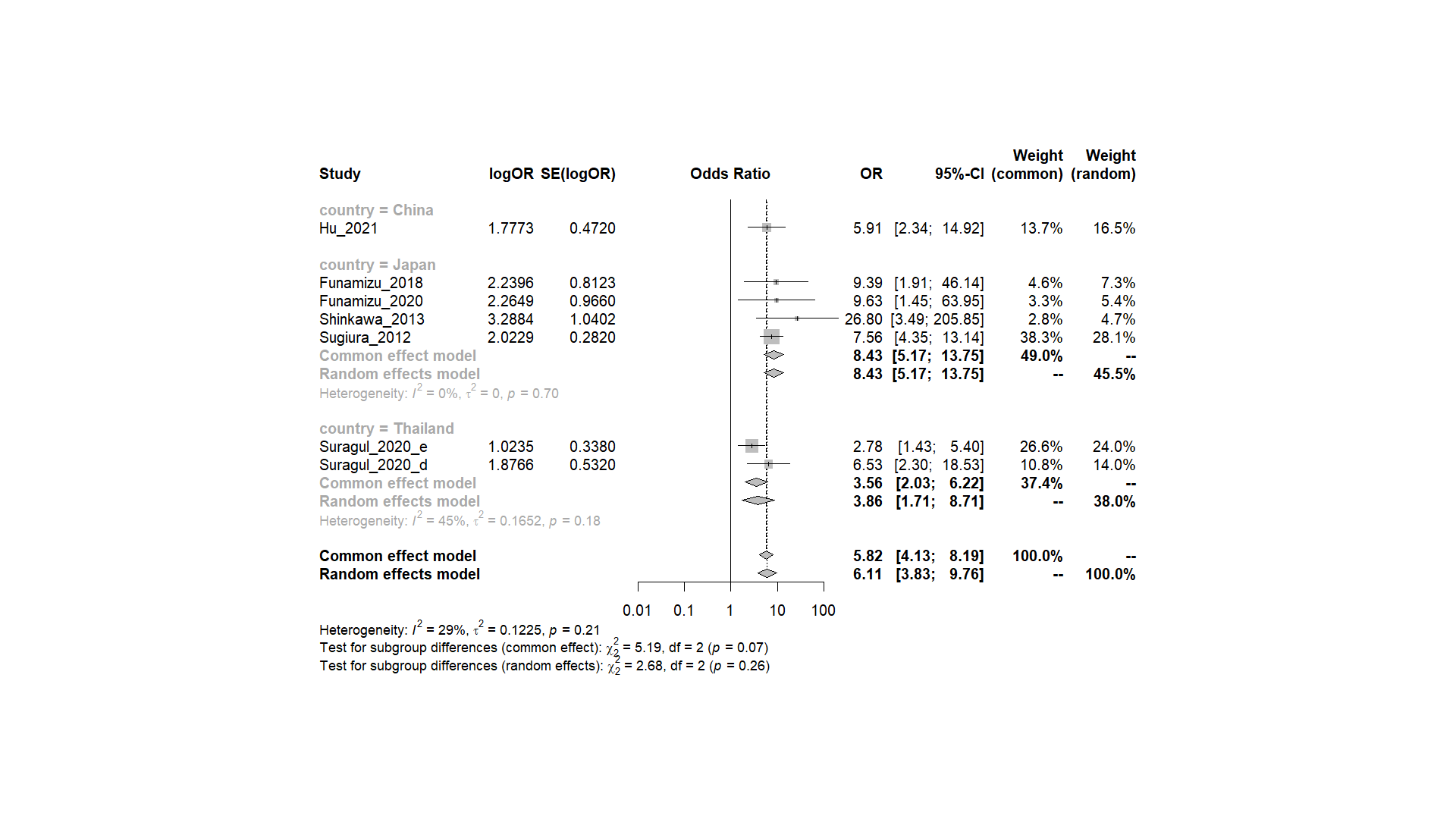 |
| **F. Pancreatic texture (Soft vs Hard)** |
| 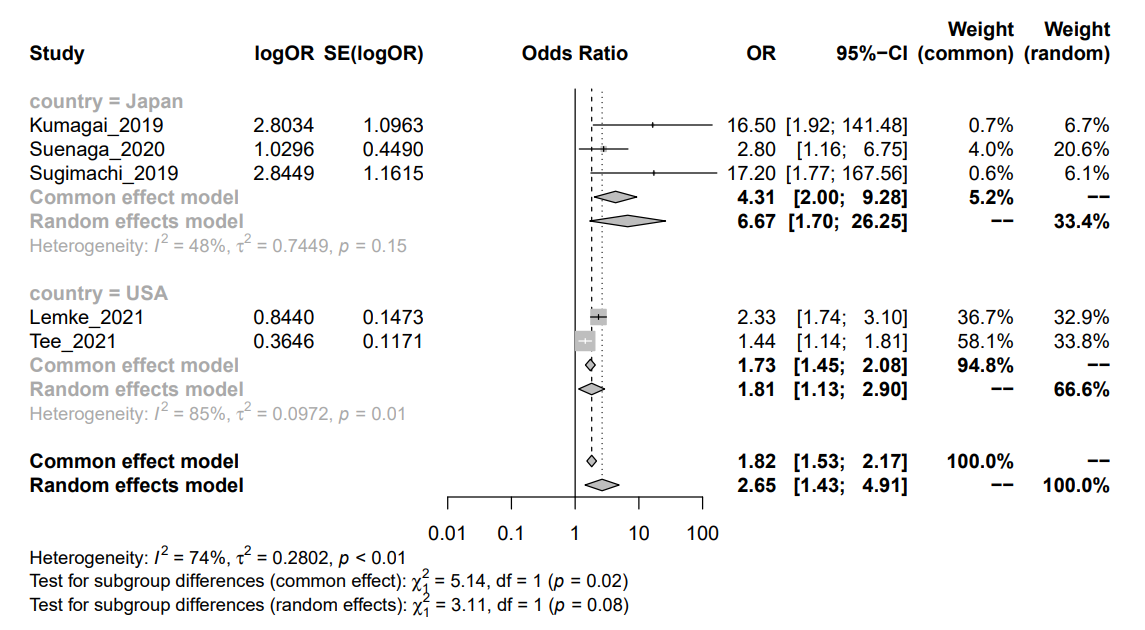 |
| **G. Preoperative albumin (Low level vs High)** |
| 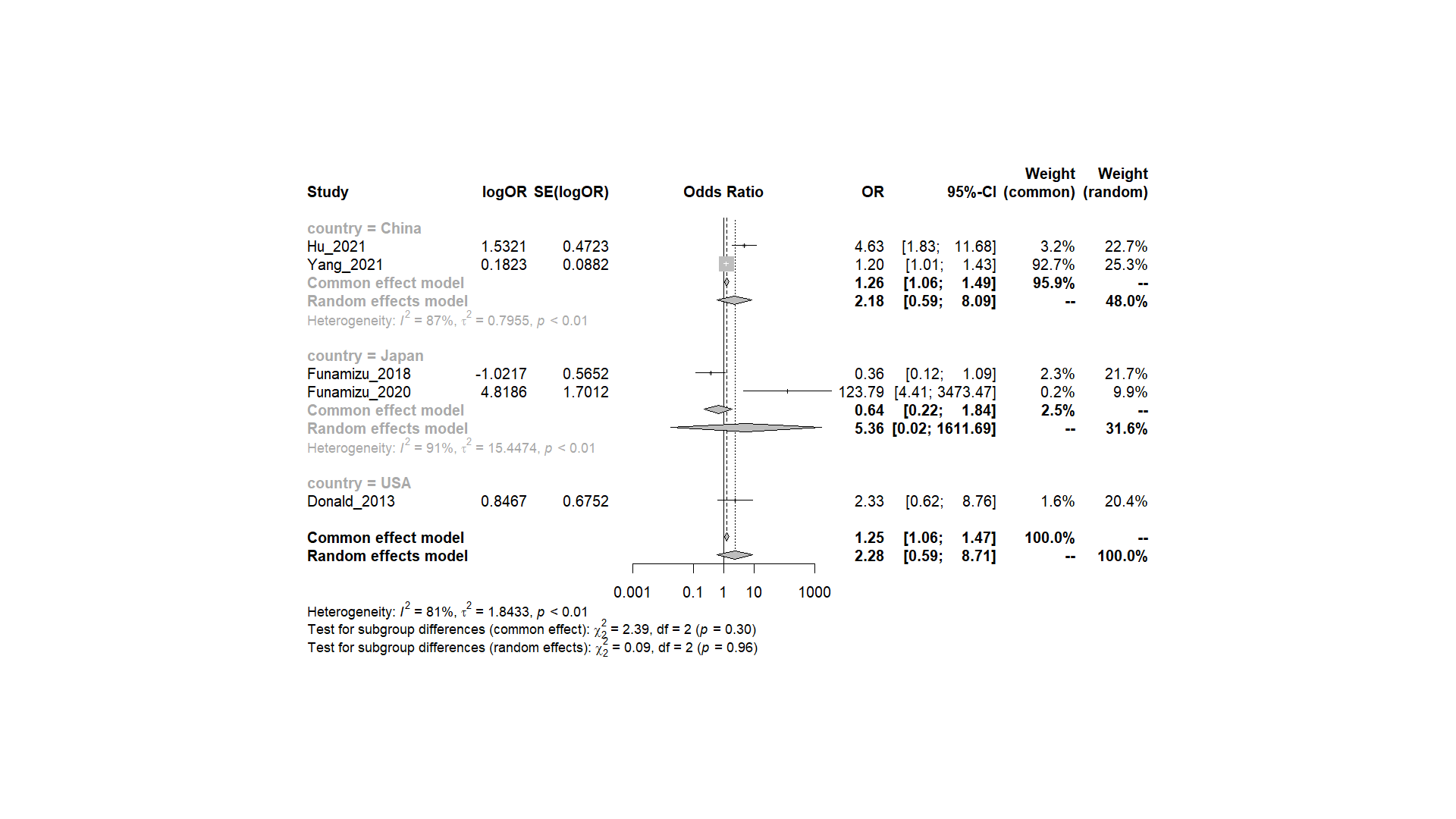 |
| **H. Diabetes mellitus** |
| 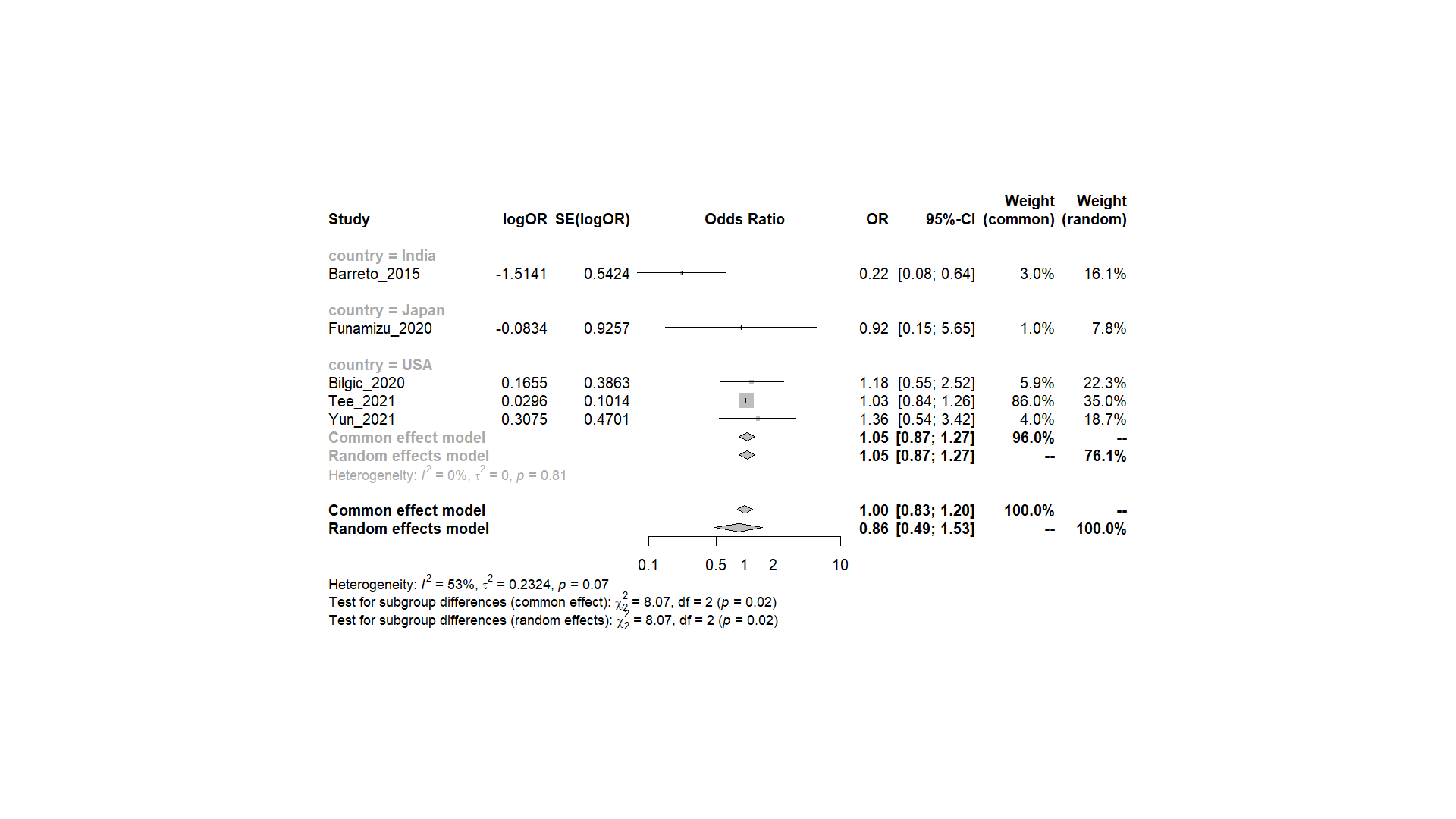 |
| **I. Age (Older vs Young)** |
| 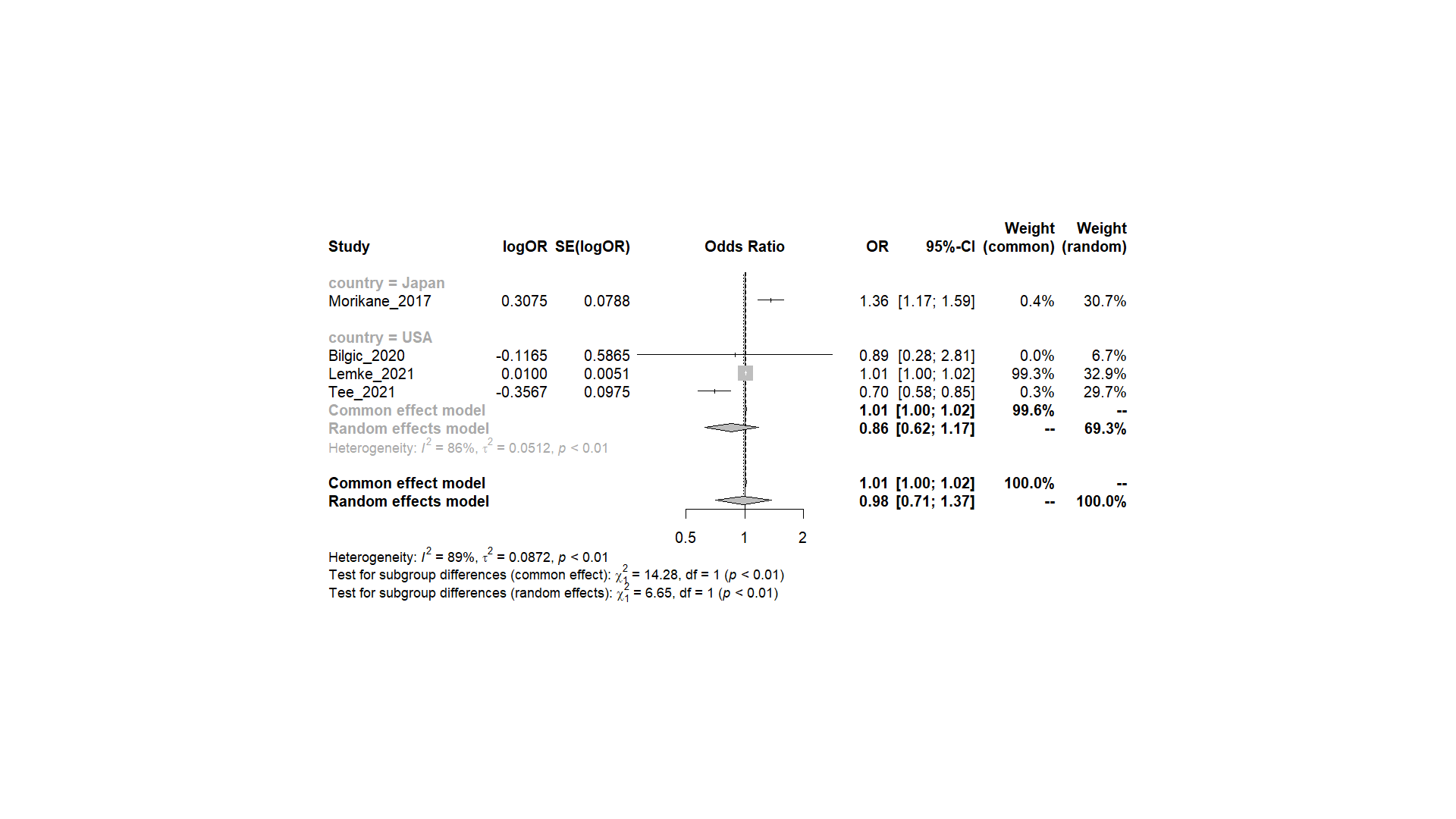 |
| **Figure 6.** Forest plots of risk factors of SSIs grouped by region |

### Group variables by study design

| **A. Preoperative biliary stenting** |
| --- |
| 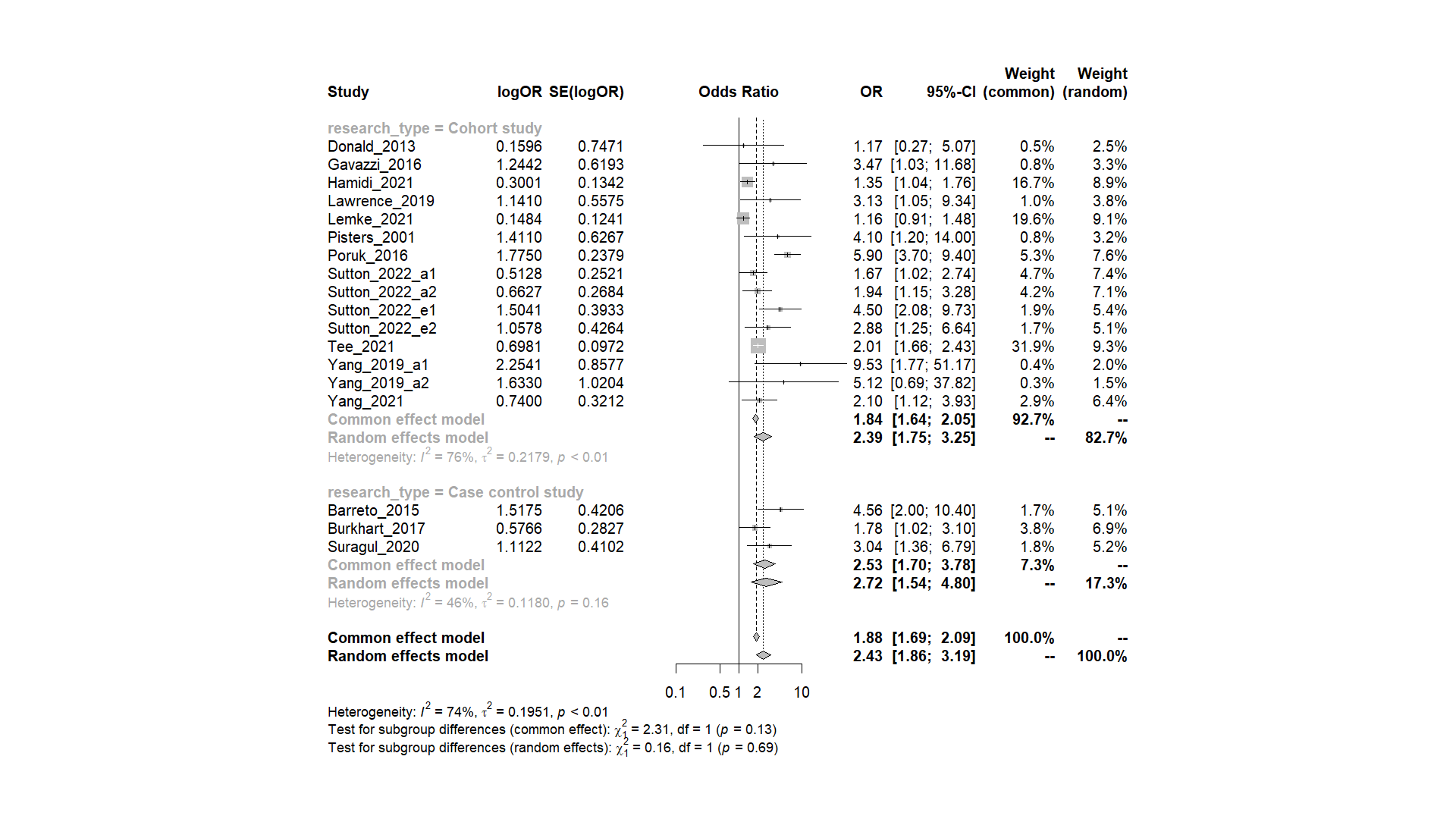 |
| **B. Body mass index (High vs Low)** |
| 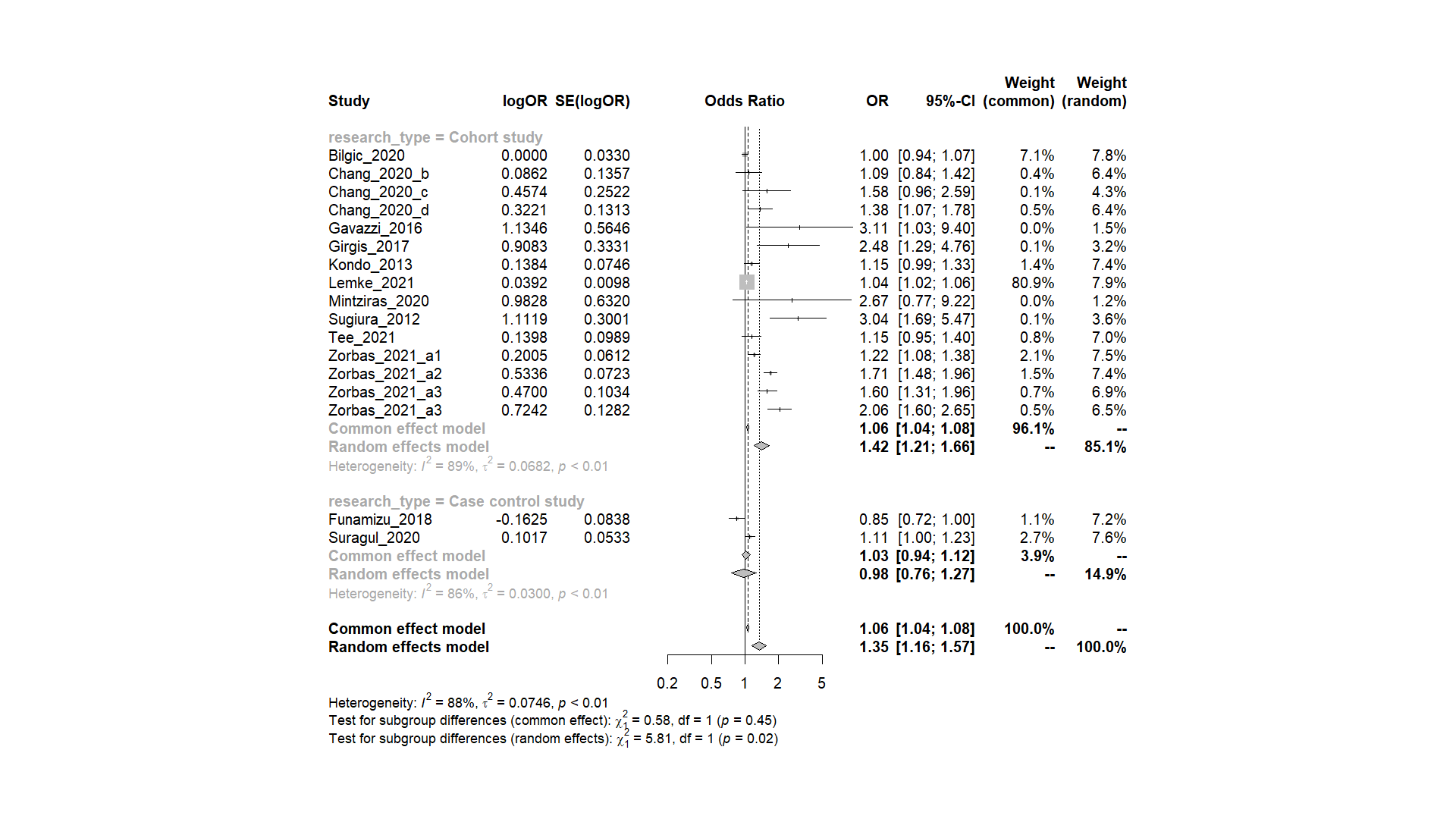 |
| **C. Operation time (Long vs Short)** |
| 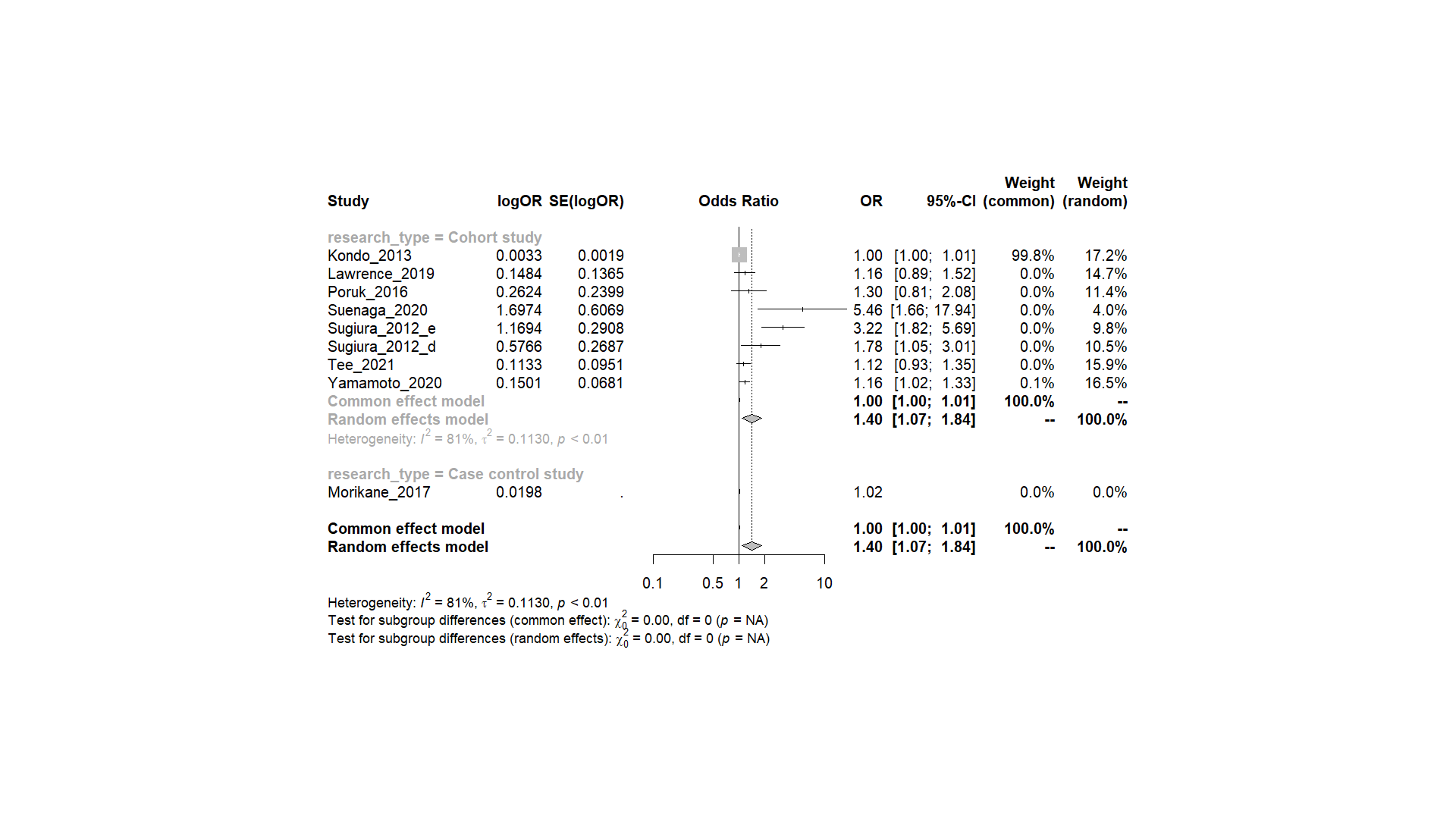 |
| **D Sex (Male vs Female)** |
| 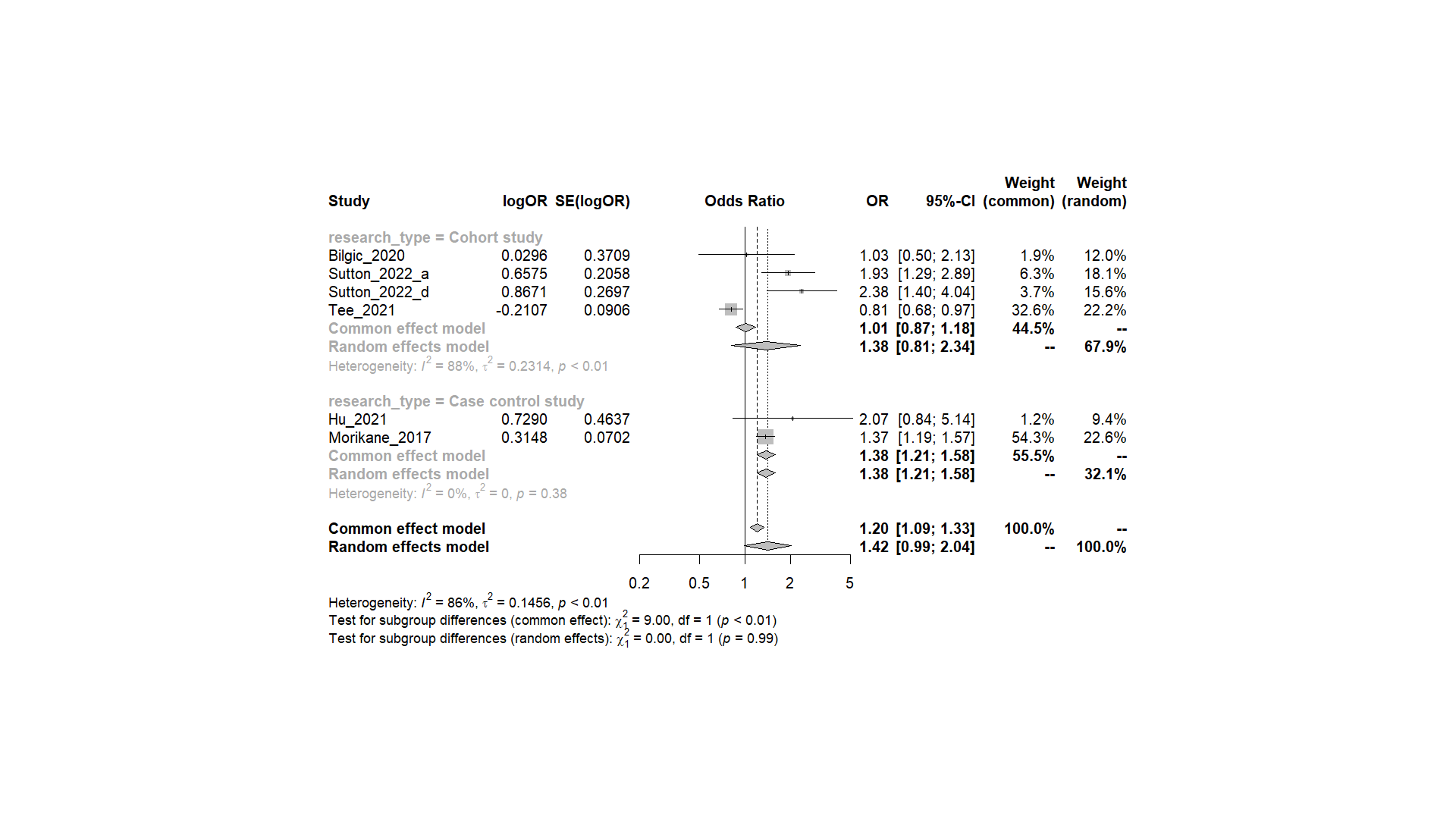 |
| **E. Postoperative pancreatic fistula** |
| 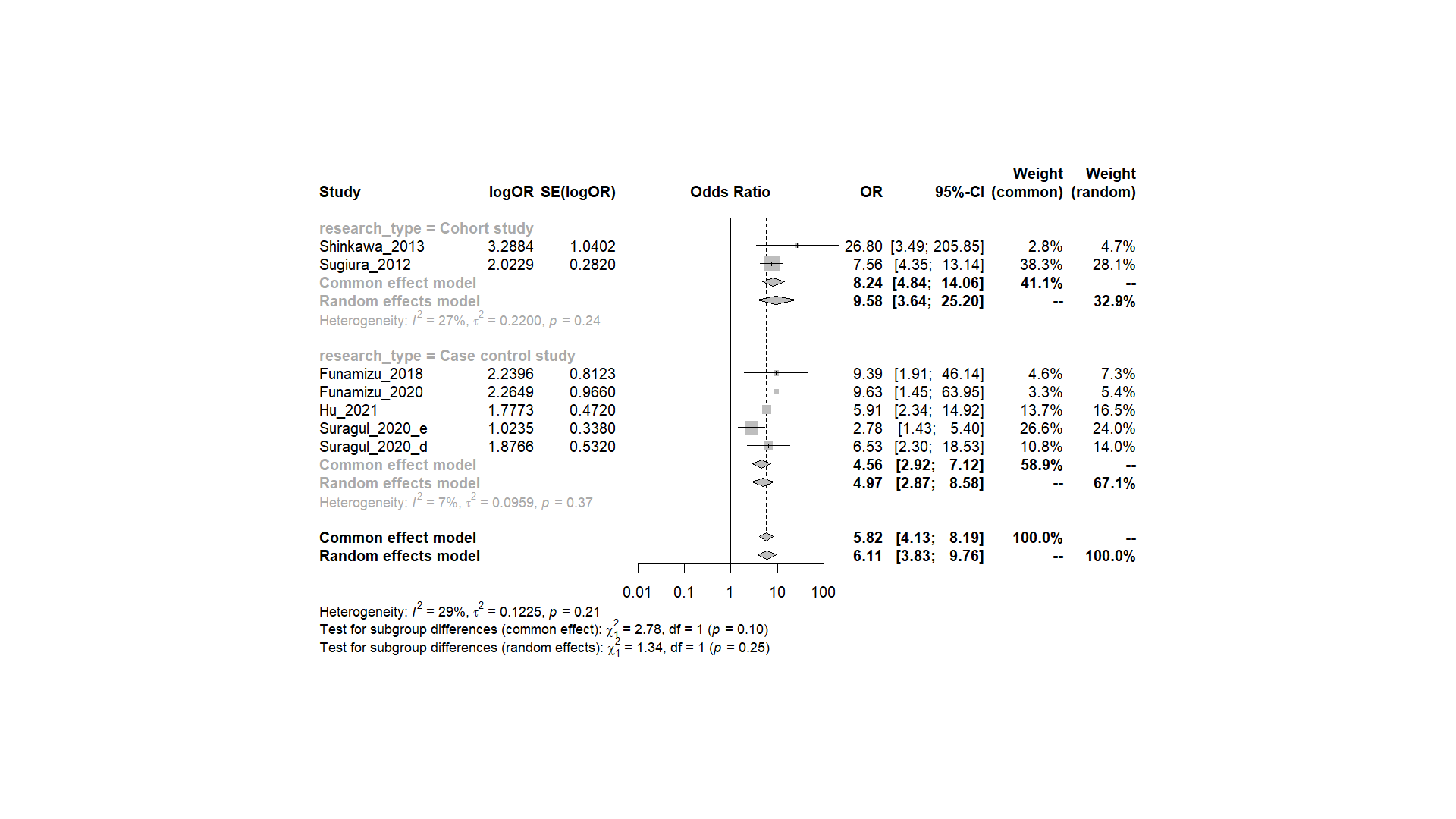 |
| **F. Preoperative albumin (Low level vs High)** |
| 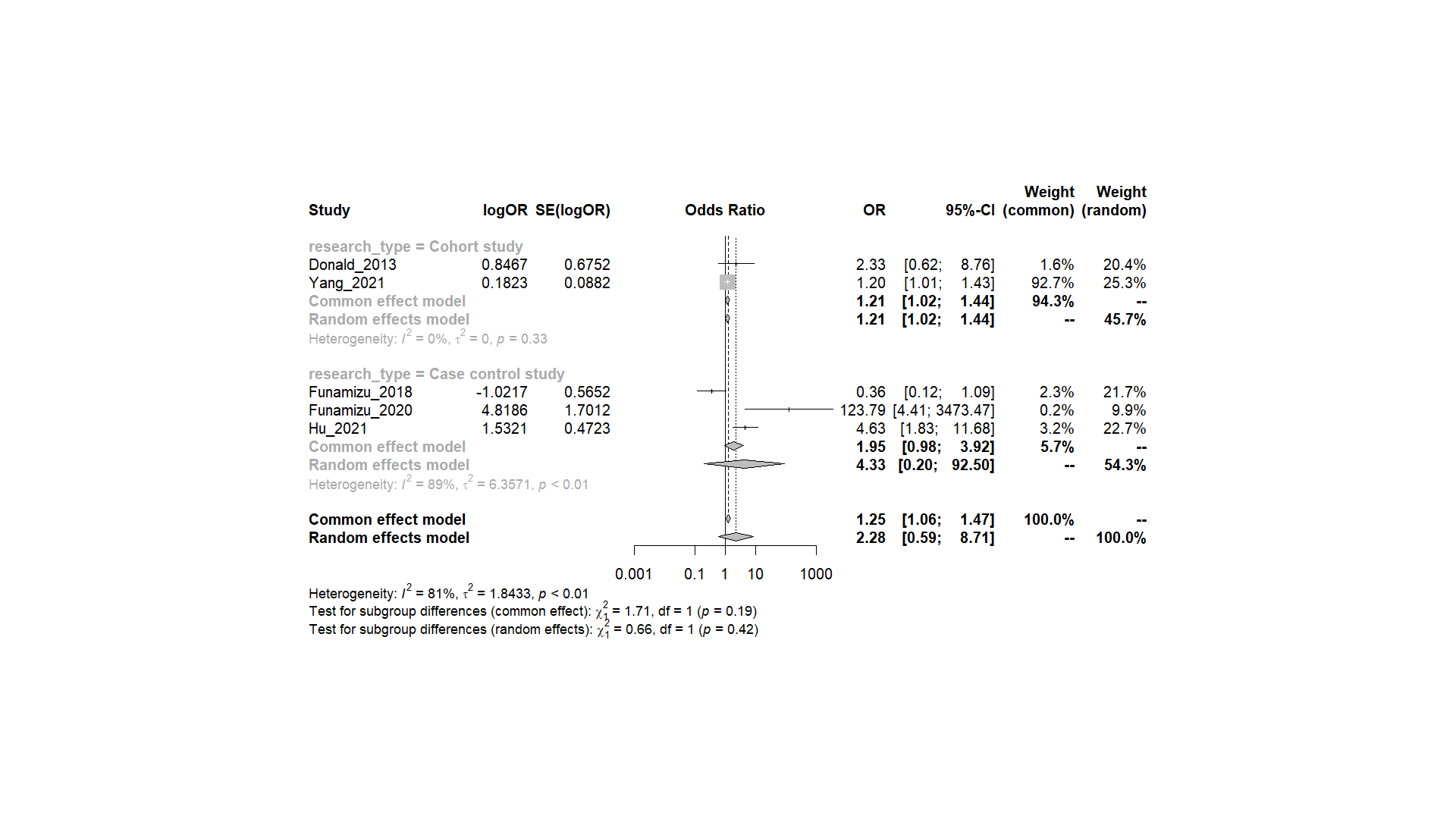 |
| **G. Diabetes mellitus** |
| 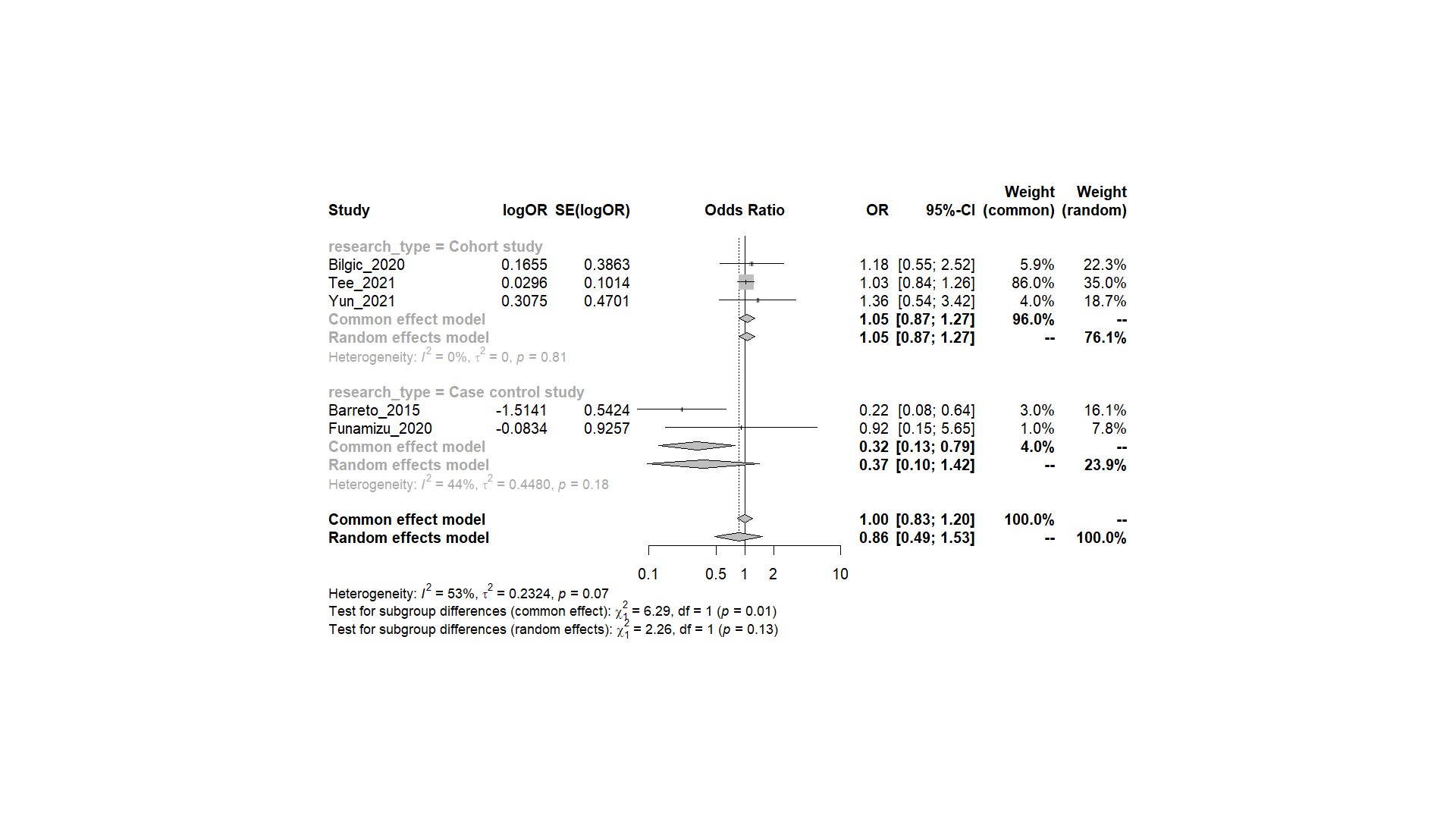 |
| **H. Age (Older vs Young)** |
| 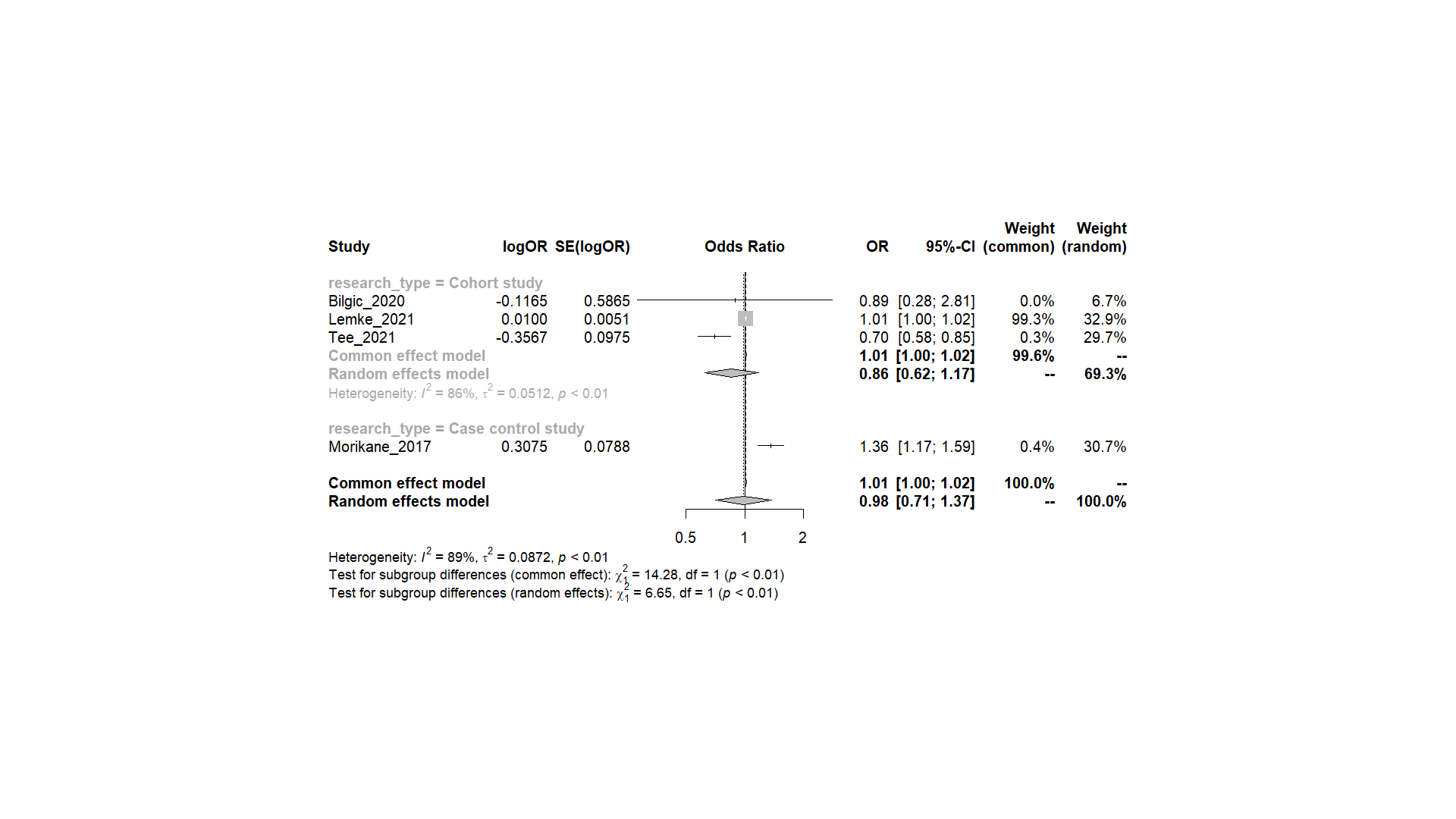 |
| **Figure 7.** Forest plots of risk factors of SSIs grouped by study design |
| *The studies on pancreatic texture analysis are all cohort study. |

### Group variables by type of SSIs

| **A. Preoperative biliary stenting** |
| --- |
| 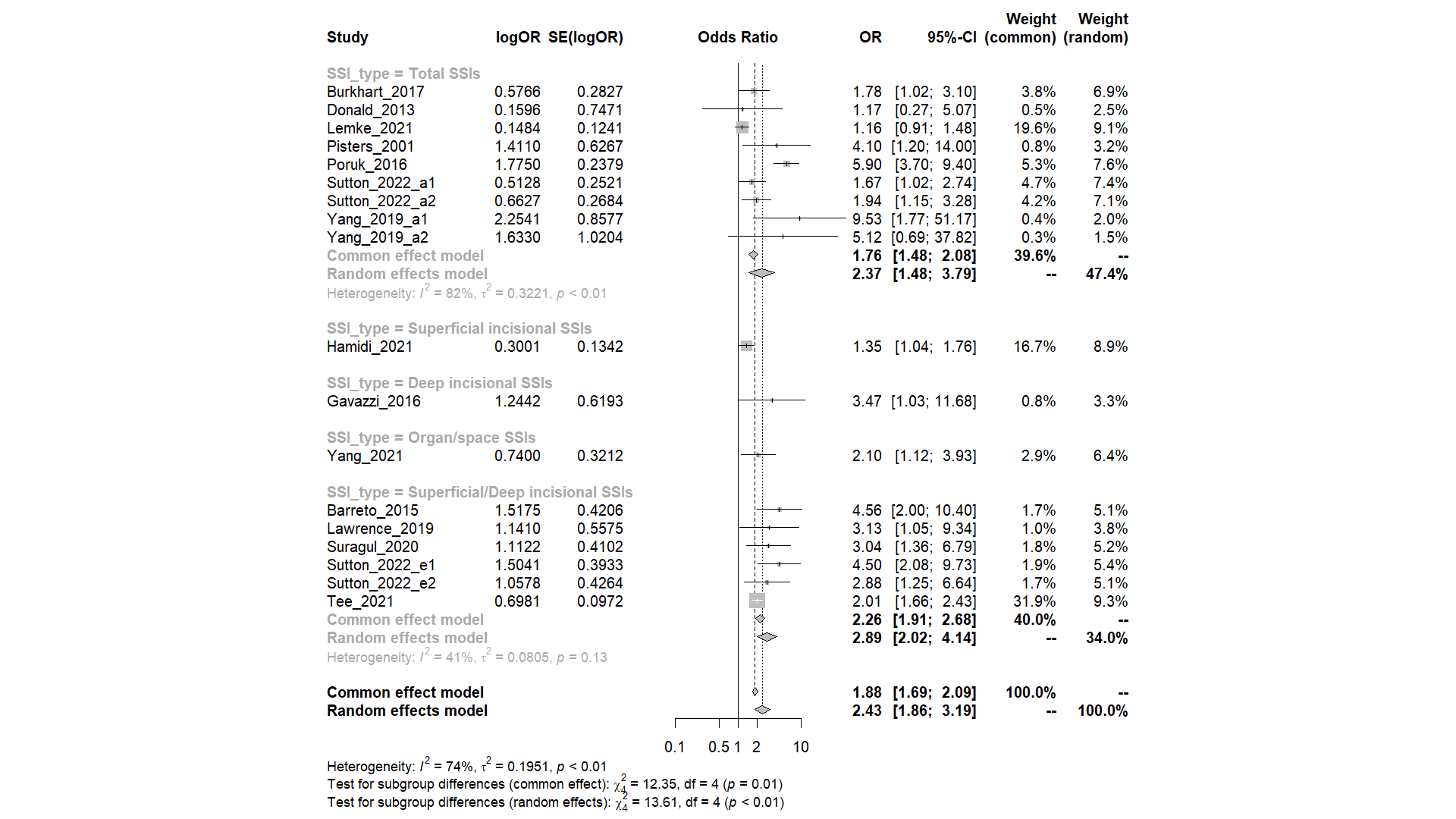 |
| **B. Body mass index (High vs Low)** |
| 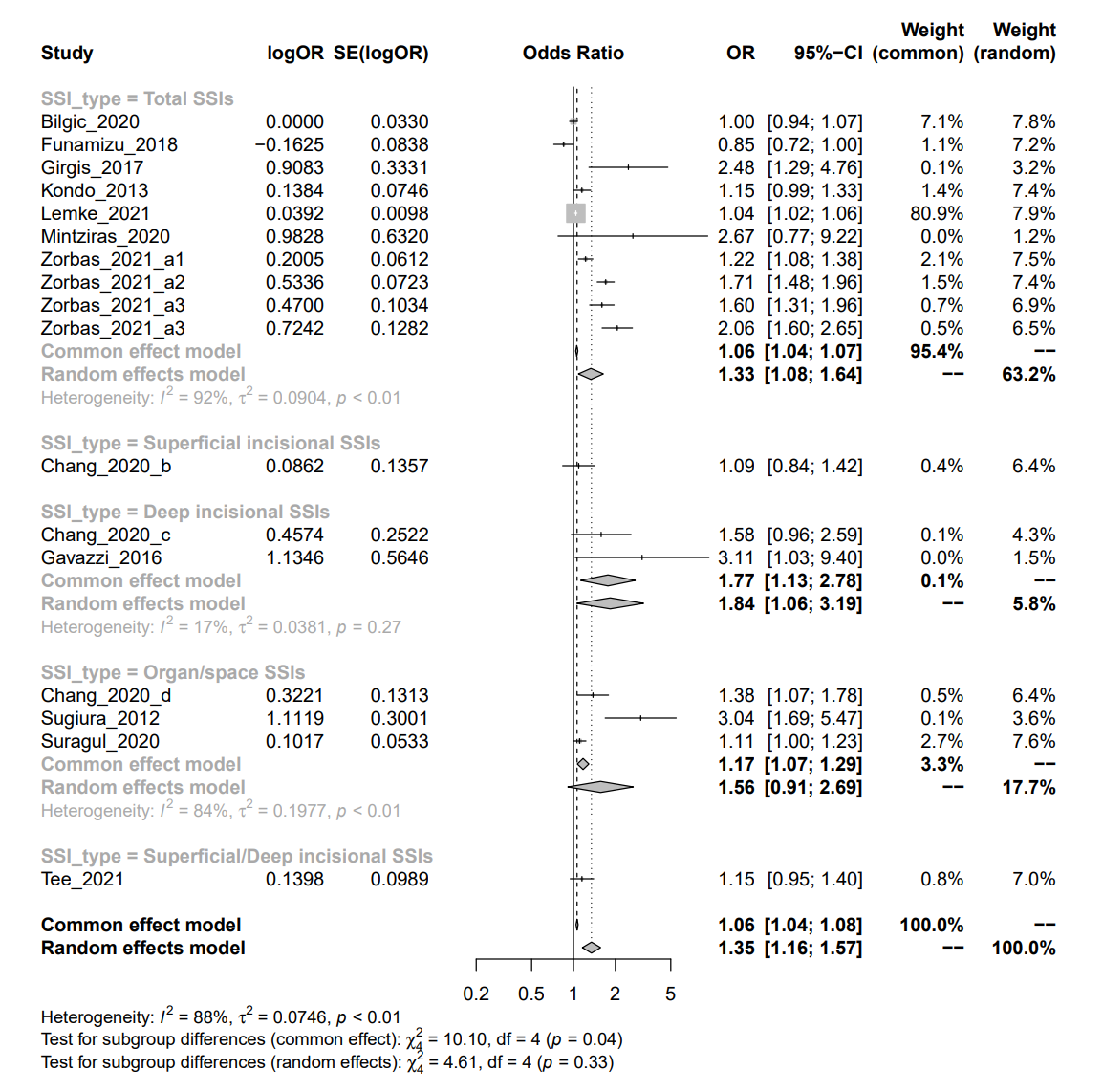 |
| **C. Operation time (Long vs Short)** |
| 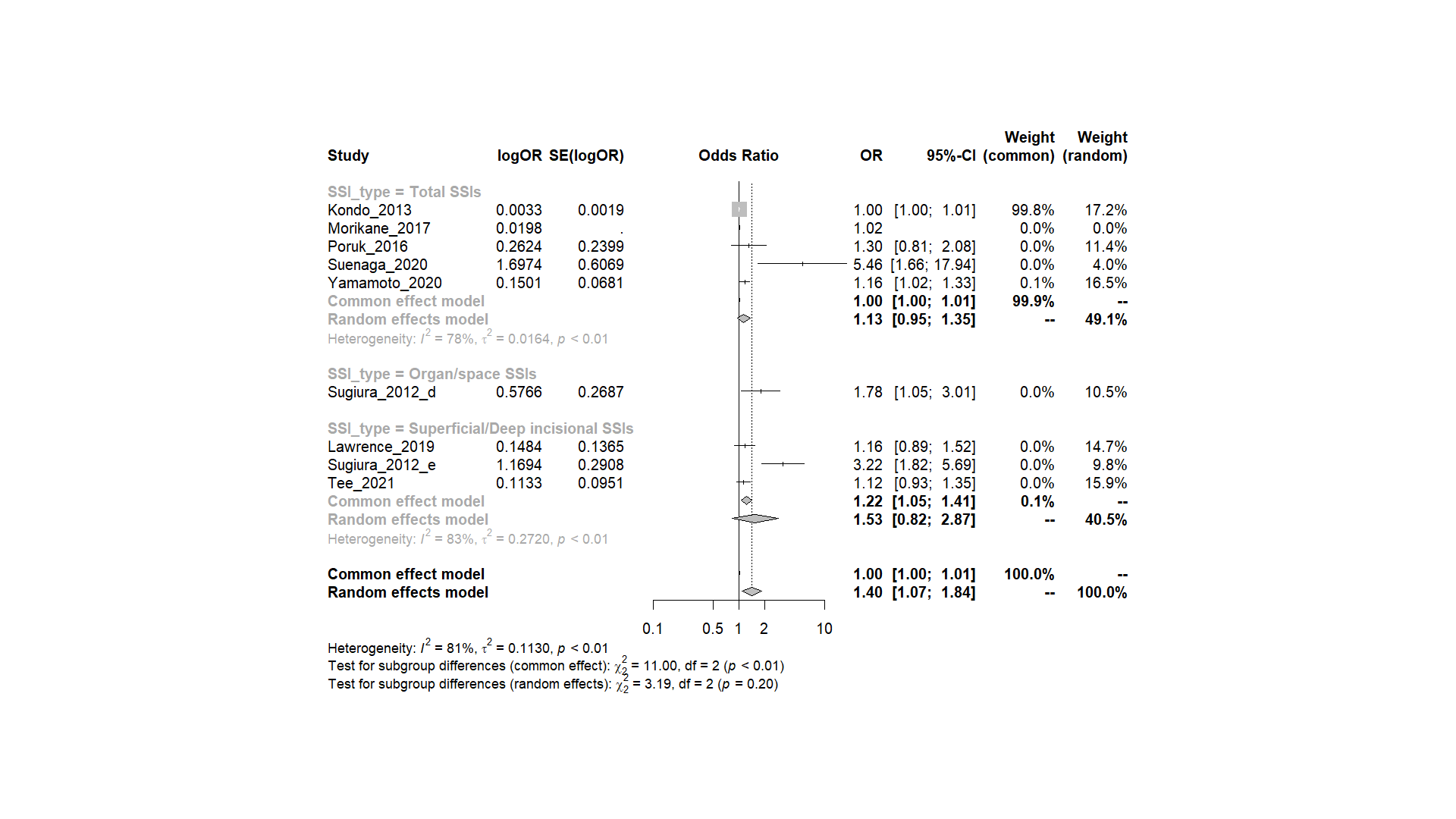 |
| **D. Sex (Male vs Female)** |
| 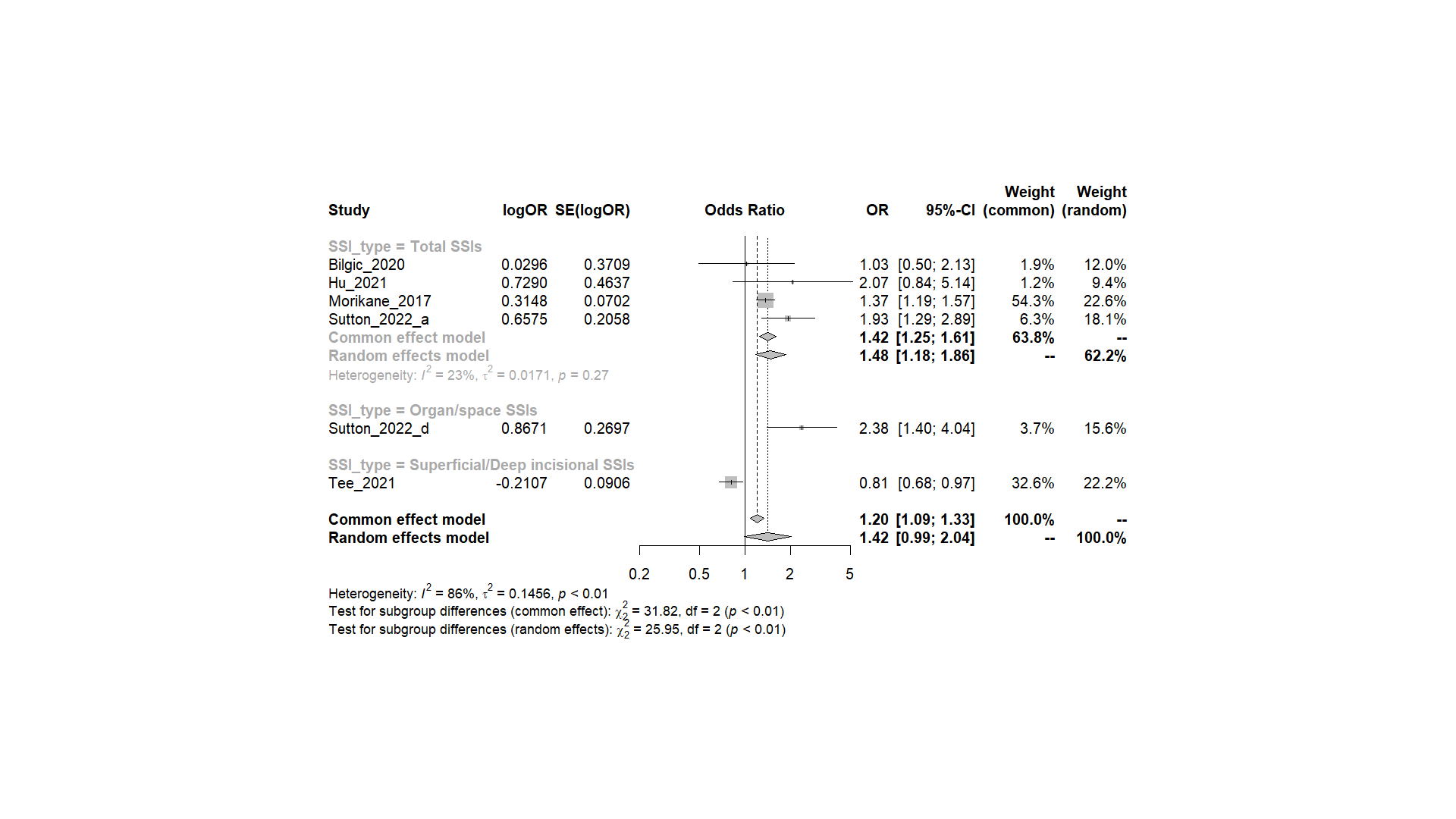 |
| **E. Postoperative pancreatic fistula** |
| 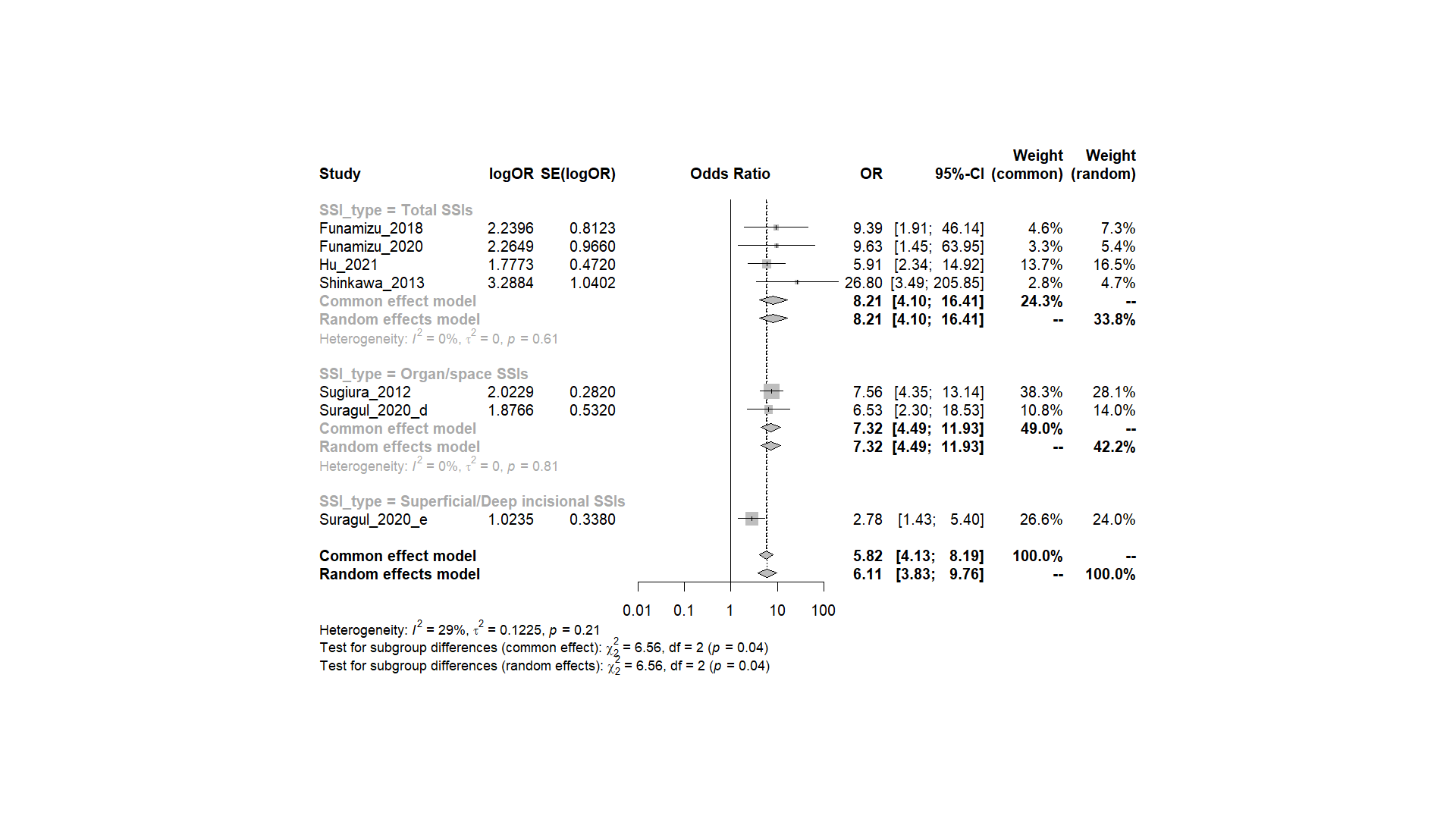 |
| **F. Pancreatic texture (Soft vs Hard)** |
| 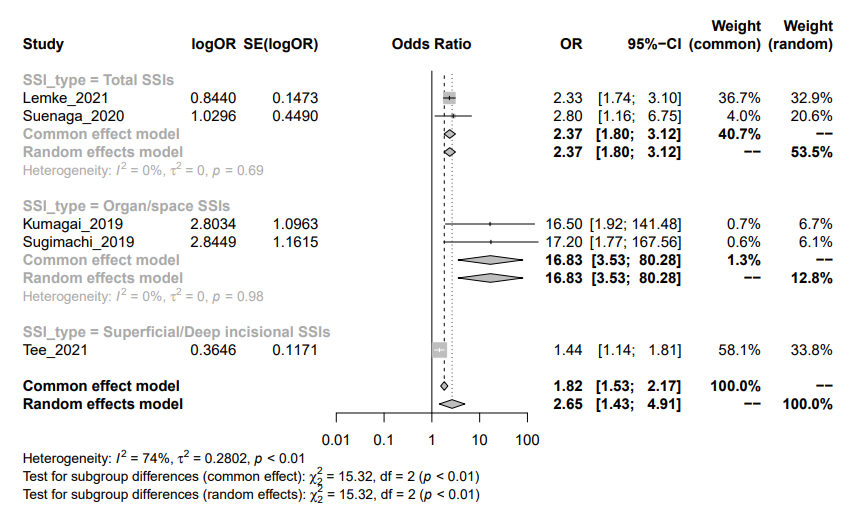 |
| **G. Preoperative albumin (Low level vs High)** |
| 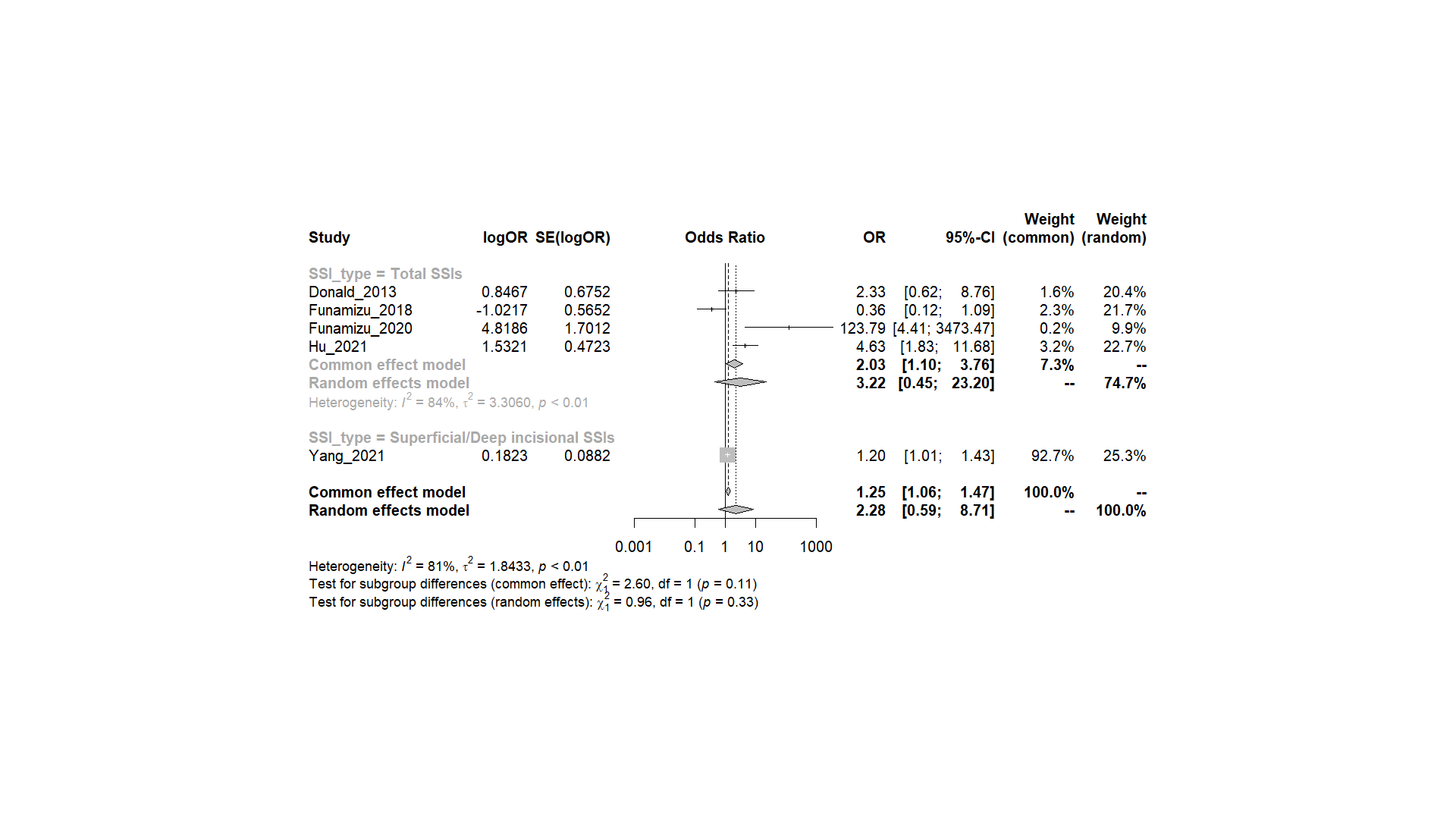 |
| **H. Diabetes mellitus** |
| 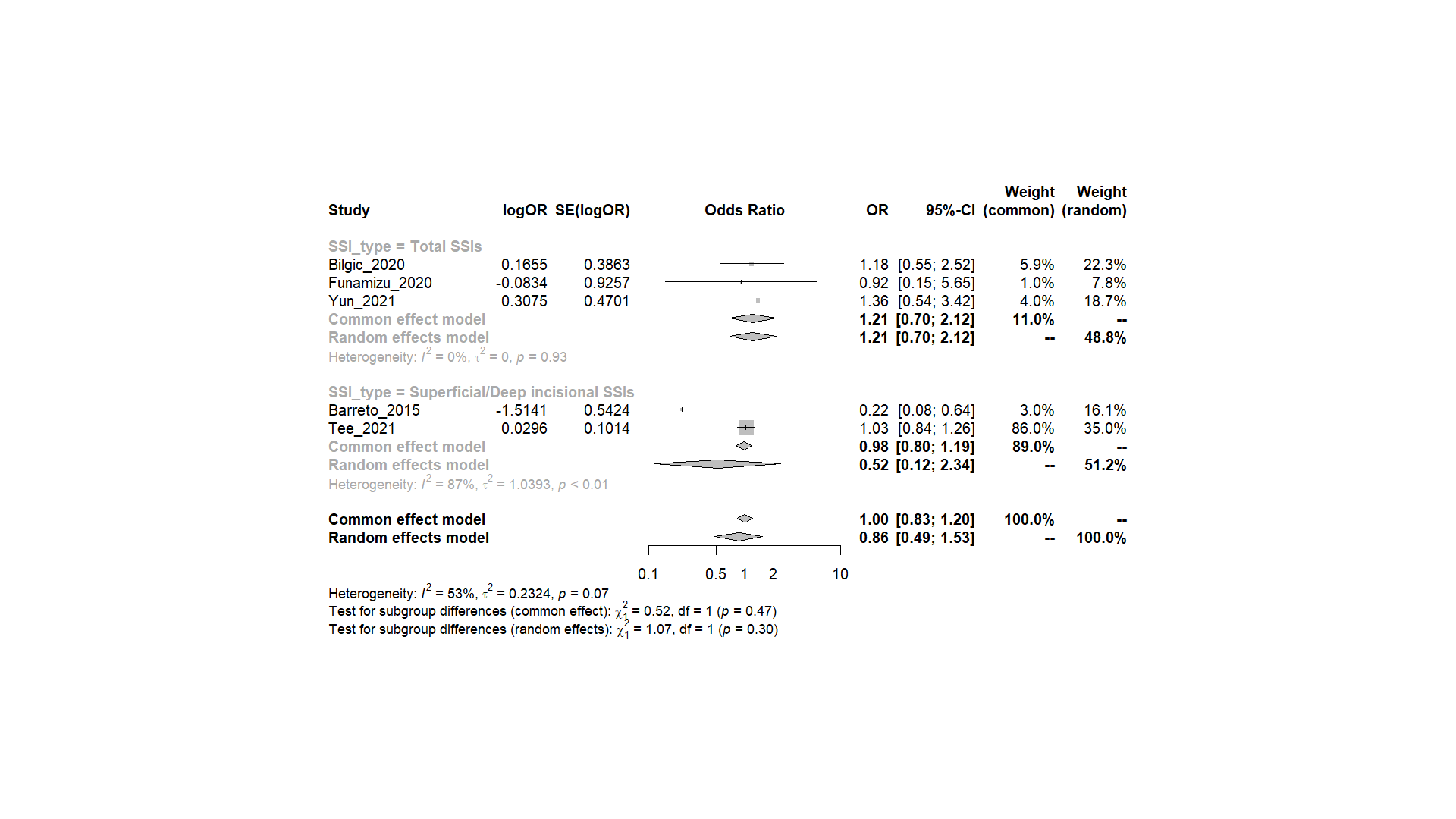 |
| **I. Age (Older vs Young)** |
| 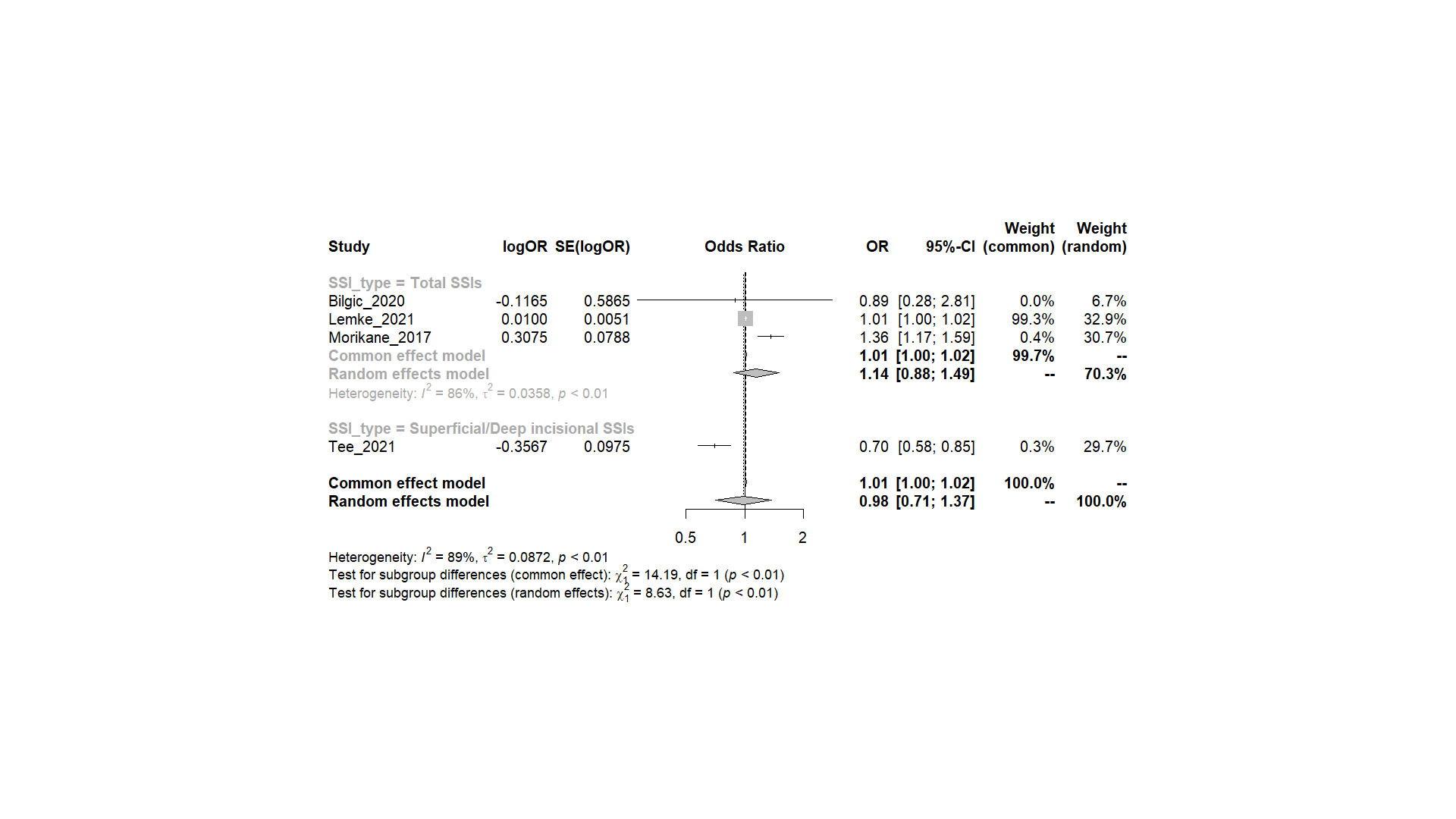 |
| **Figure 8.** Forest plots of risk factors of SSIs grouped by type of SSIs |

## Sensitivity analysis

| **Random-effect model** | **Common-effect model** |
| --- | --- |
| **A. Preoperative biliary stenting** | |
| 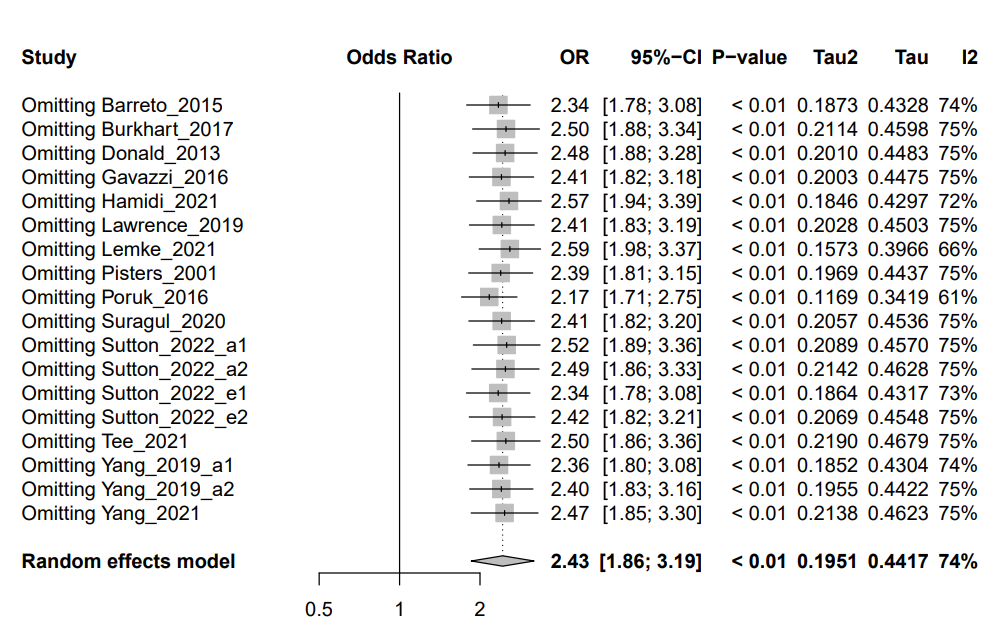 | 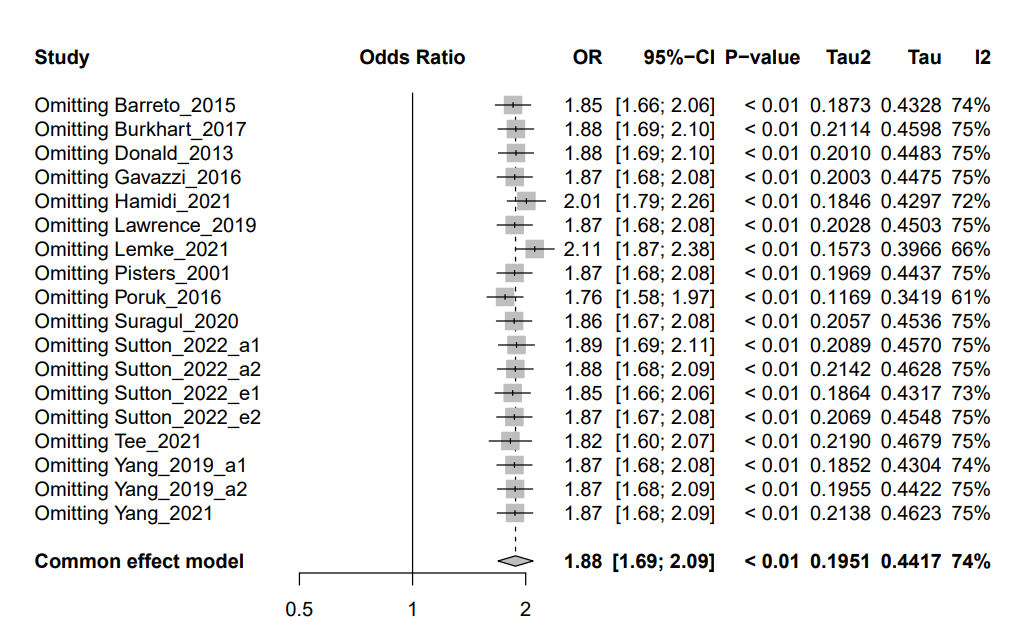 |
| **B. Body mass index (High vs Low)** | |
| 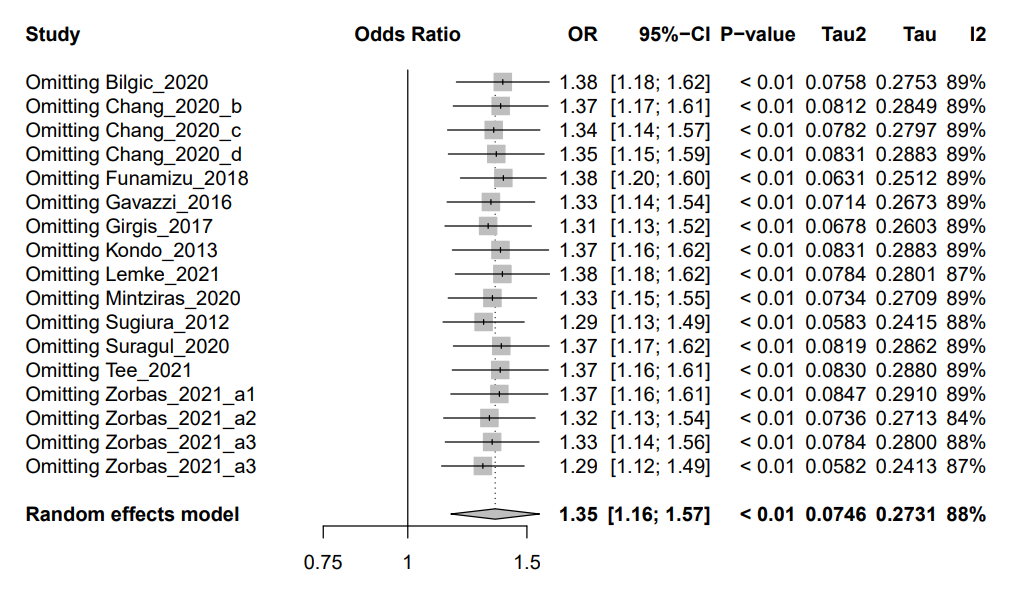 | 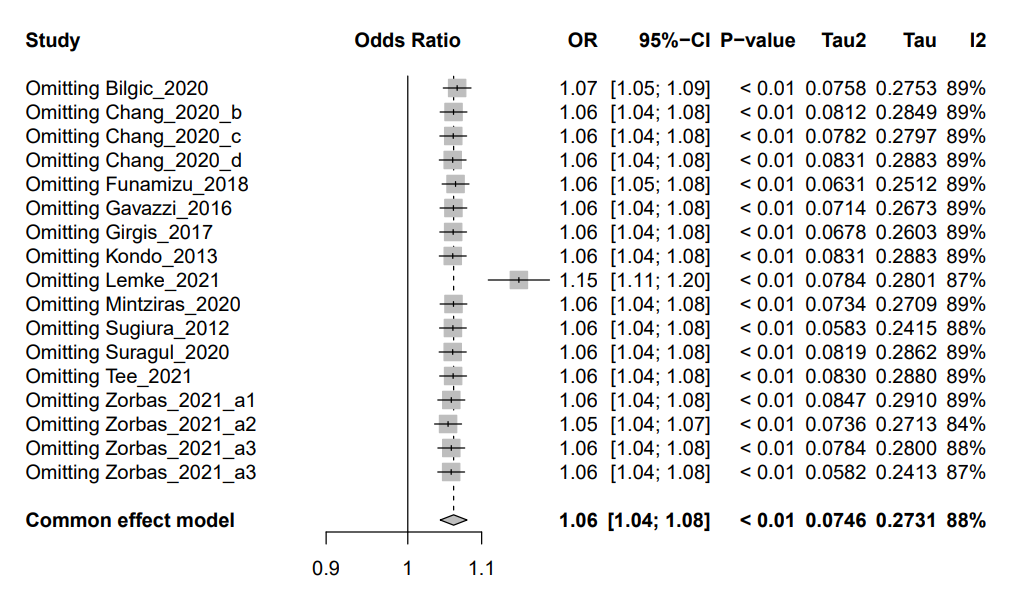 |
| **C1. Antibiotic prophylaxis (second- or third-generation cephalosporin vs First-generation)** | |
| 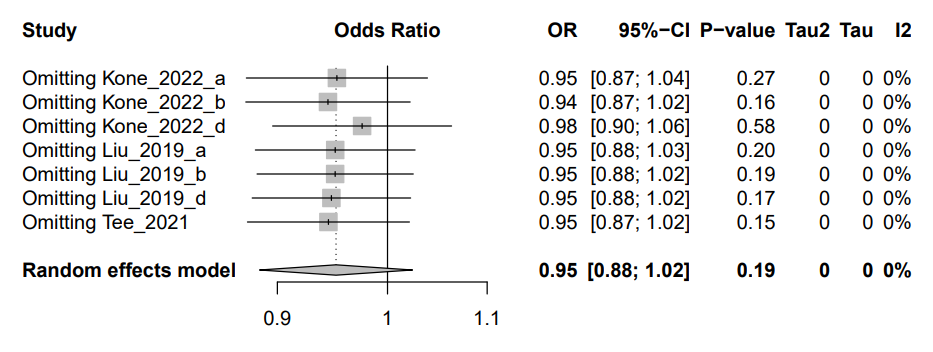 | 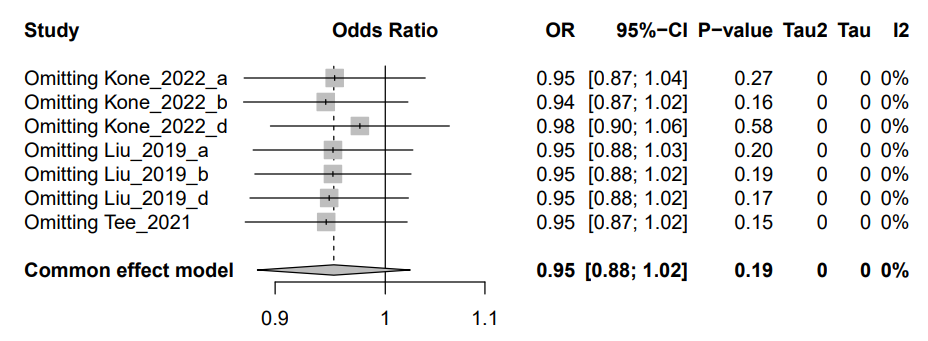 |
| **C2. Antibiotic prophylaxis (broad-spectrum antibiotics vs First-generation)** | |
| 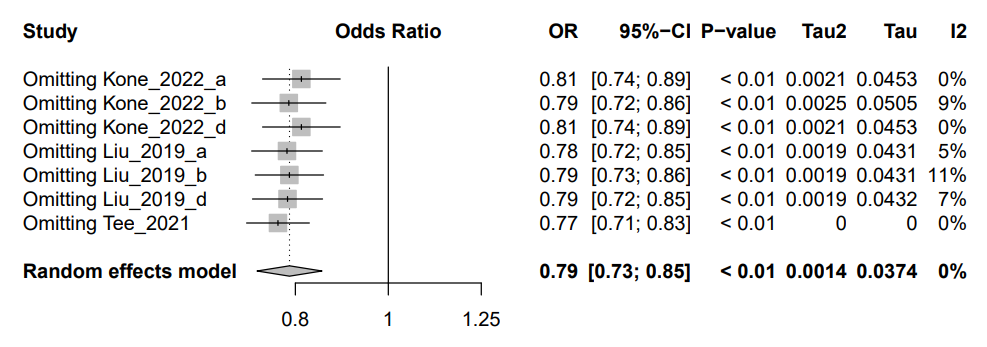 | 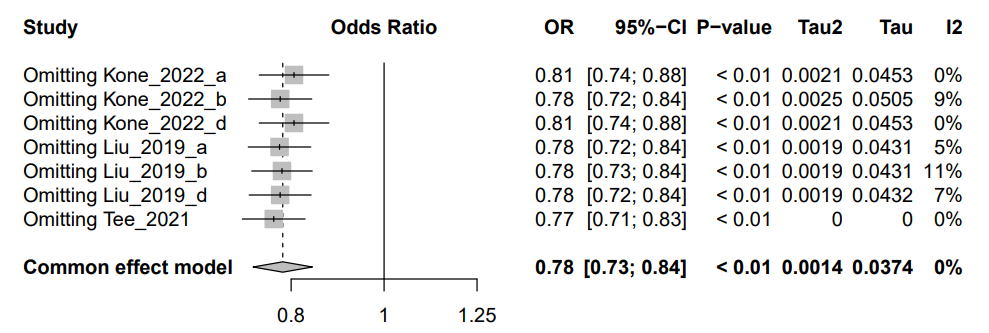 |
| **D. Operation time (Long vs Short)** |  |
| 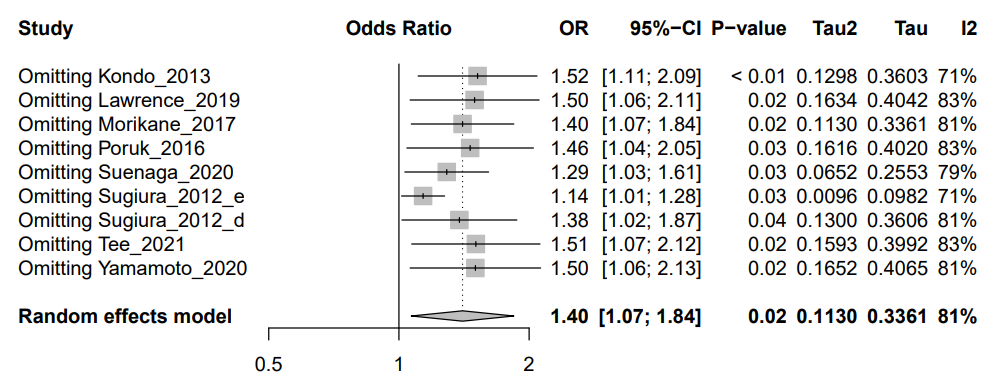 | 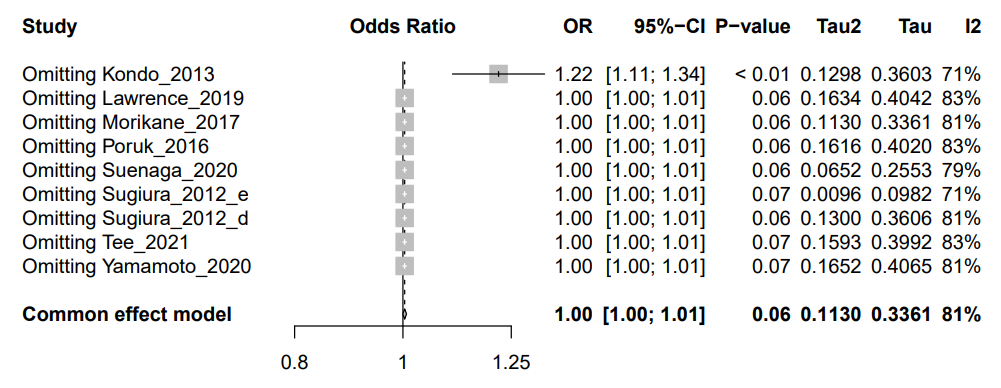 |
| **E. Perioperative blood transfusion** | |
| 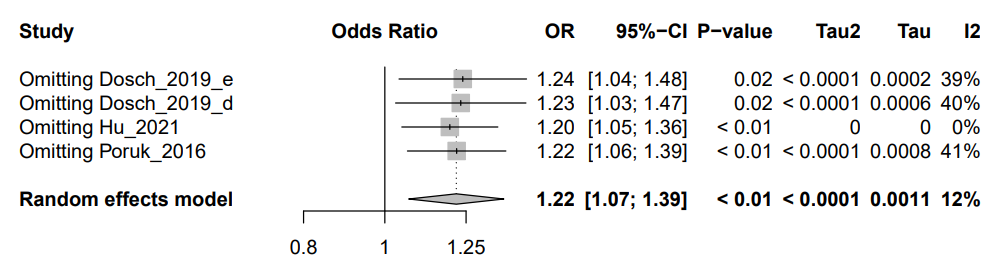 | 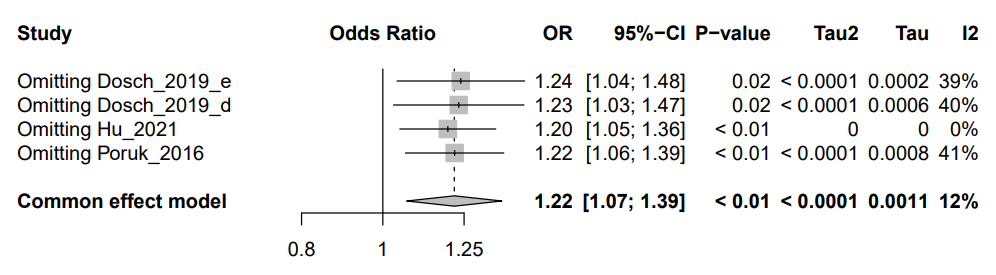 |
| **F. Sex (Male vs Female)** | |
| 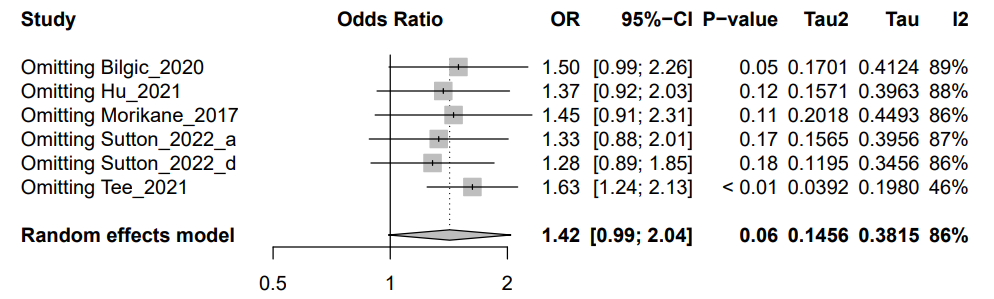 | 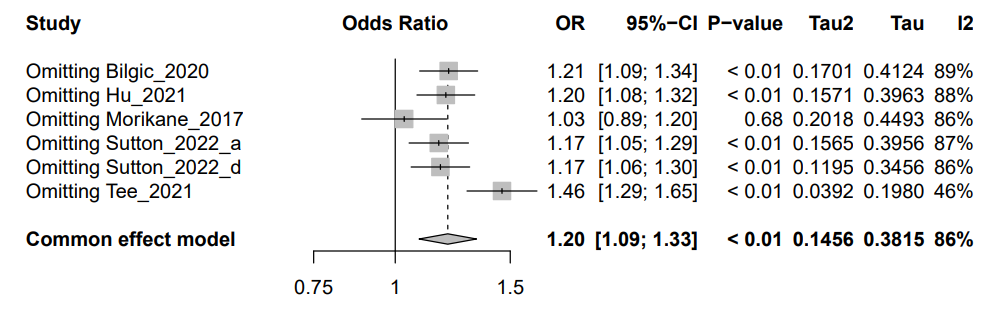 |
| **G. Postoperative pancreatic fistula** | |
| 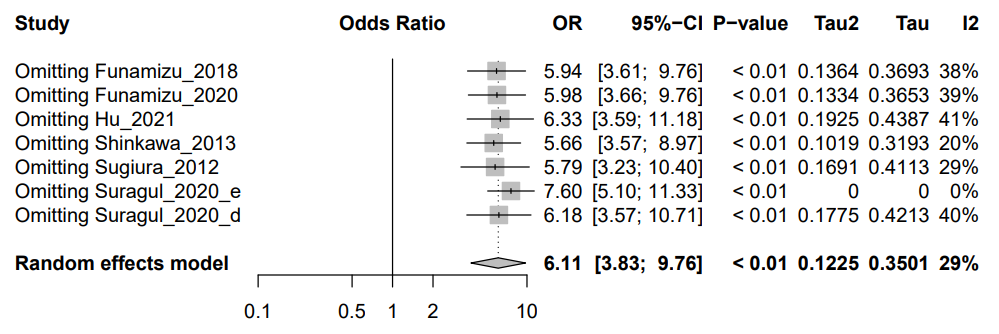 | 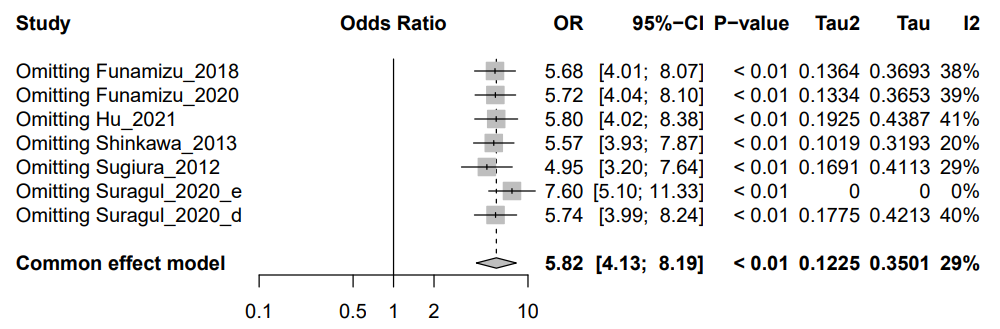 |
| **H. Pancreatic texture (Soft vs Hard)** | |
| 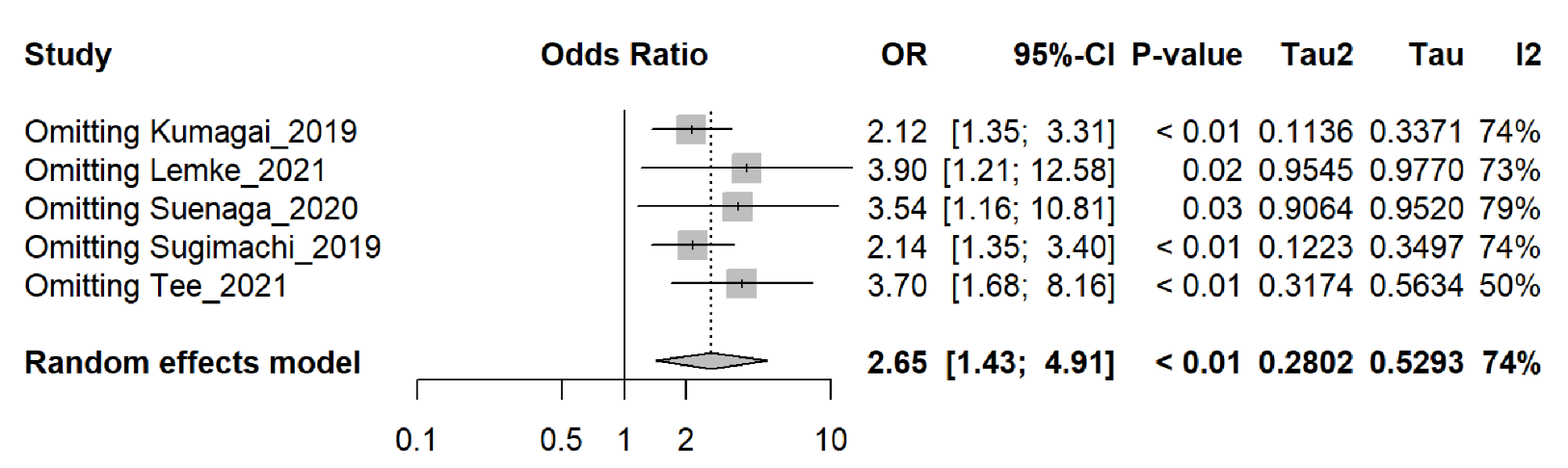 | 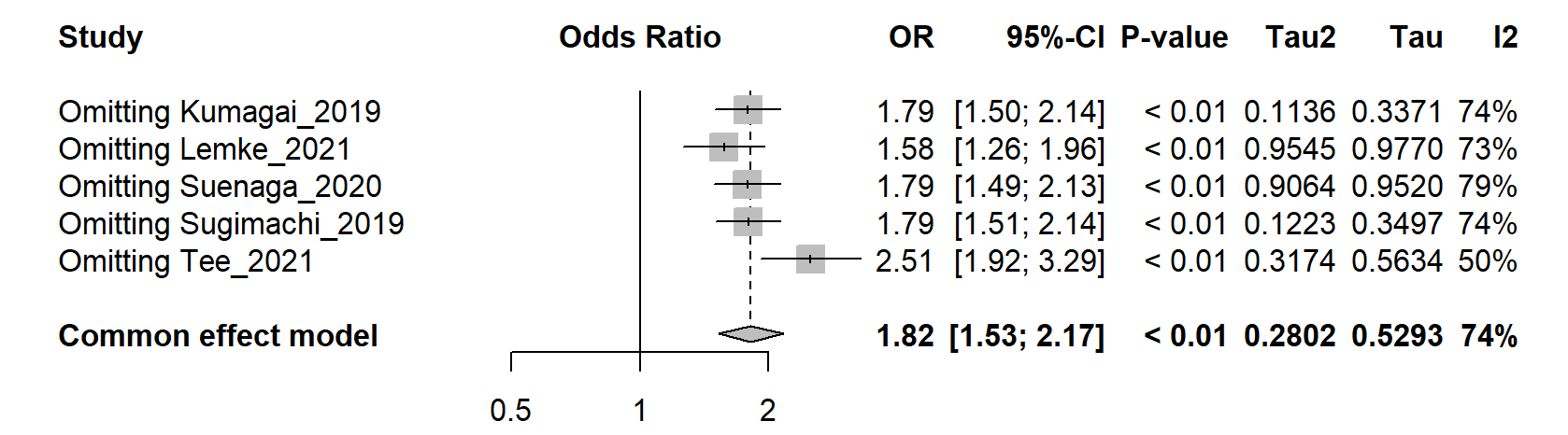 |
| **I. Preoperative albumin (Low level vs High)** | |
| 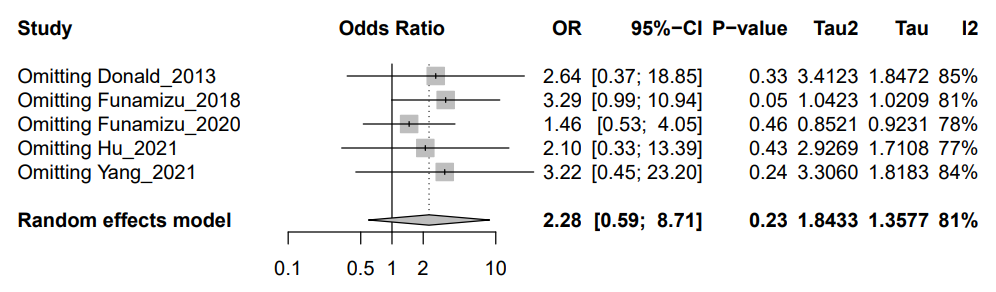 | 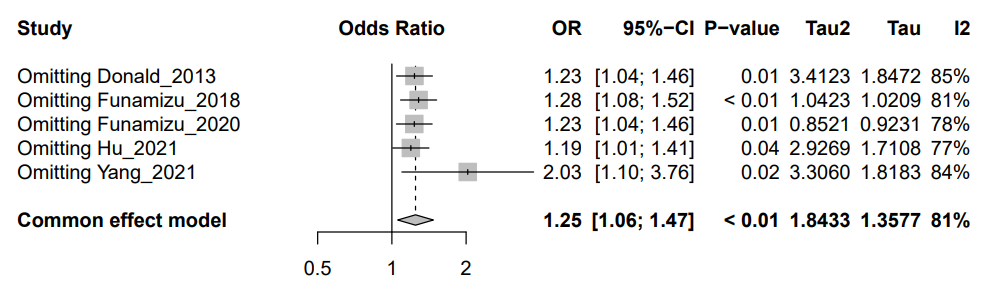 |
| **J. Diabetes mellitus** | |
| 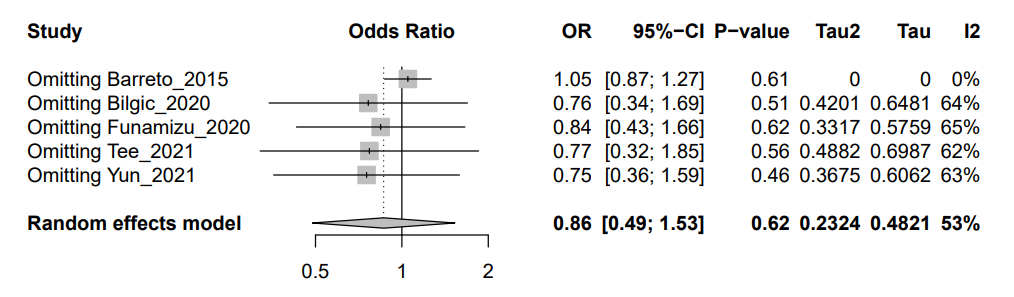 | 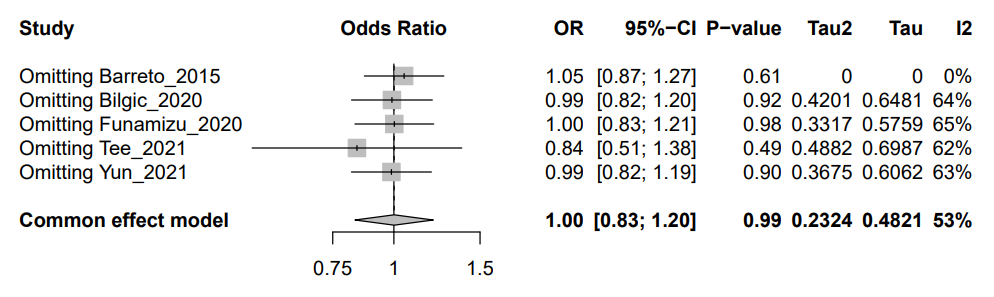 |
| **K. Cardiac disease** | |
| 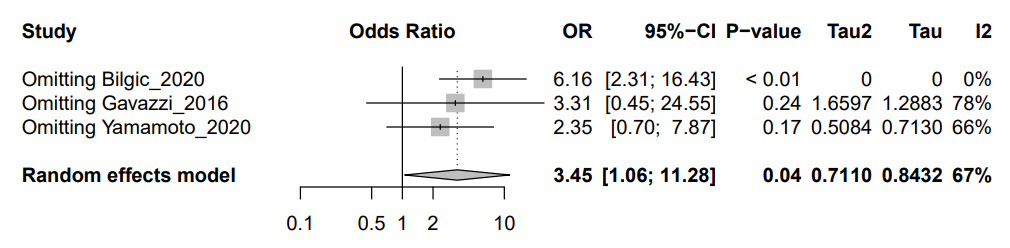 | 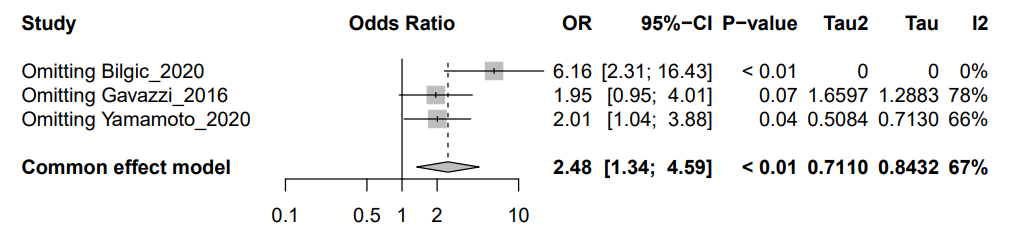 |
| **L. Age (Older vs Young)** |  |
| 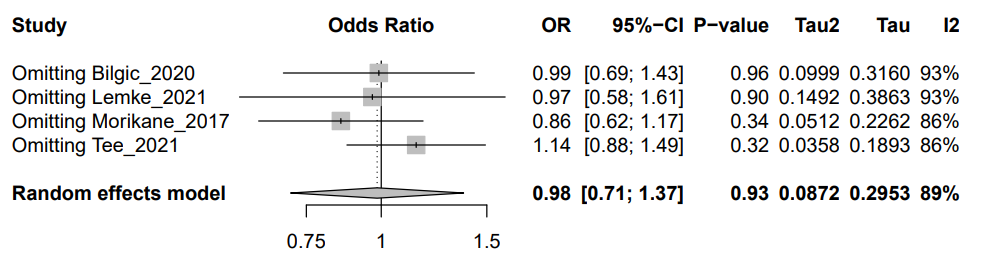 | 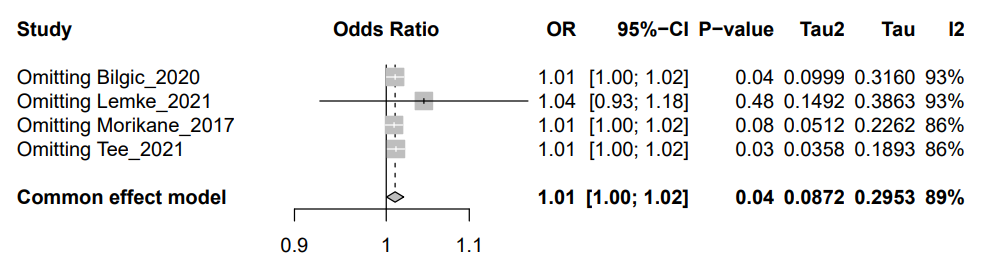 |
| **M. American Society of Anesthesiologists (ASA) classification (≥3 score vs <3score)** | |
| 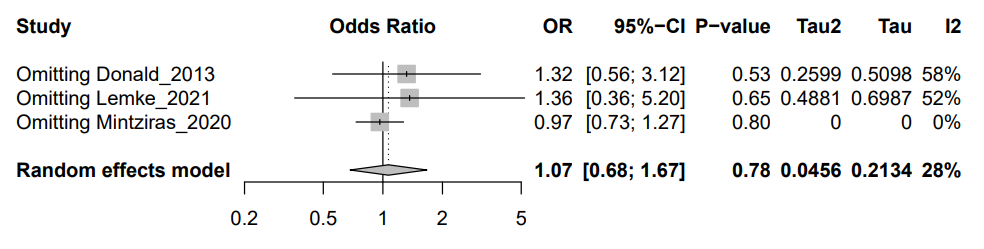 | 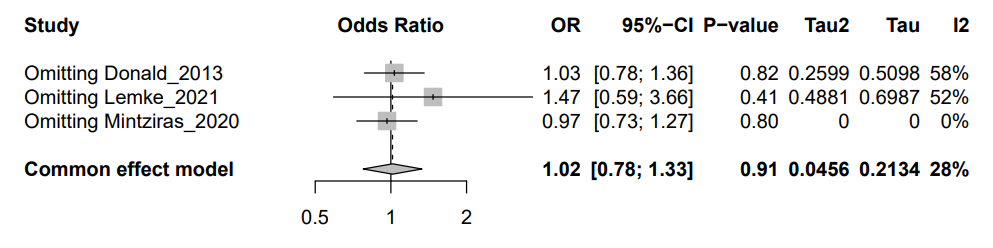 |
| **Figure 9.** Sensitivity analysis graphs of risk factors of SSIs | |

## Publication bias assessment

| **Funnel plot** | **Egger’s regression test graph** | **Trim and fill graph** |
| --- | --- | --- |
| **A. Preoperative biliary stenting** | | |
| 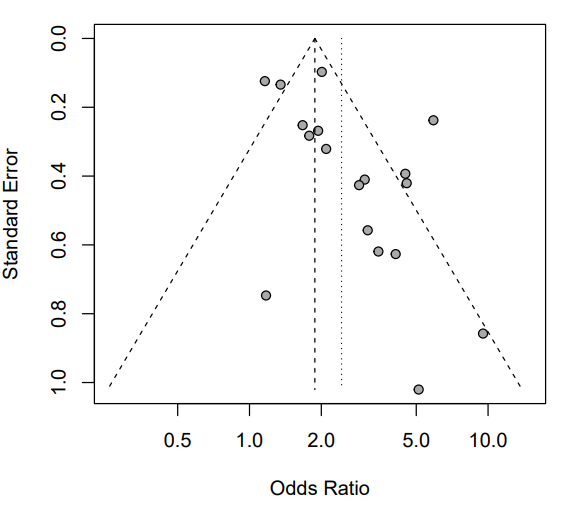 | 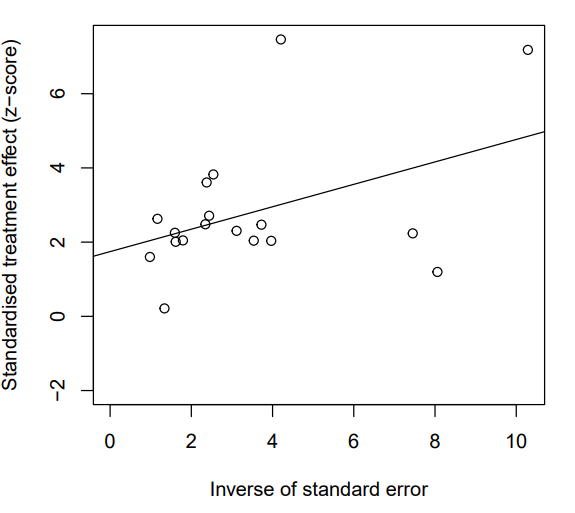 | 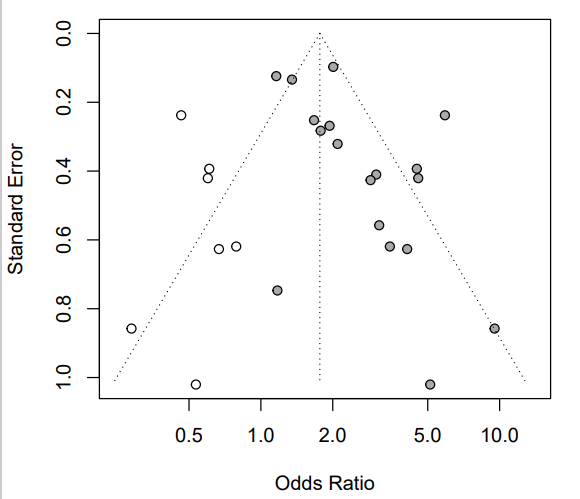 |
| **B. Body mass index (High vs Low)** | | |
| 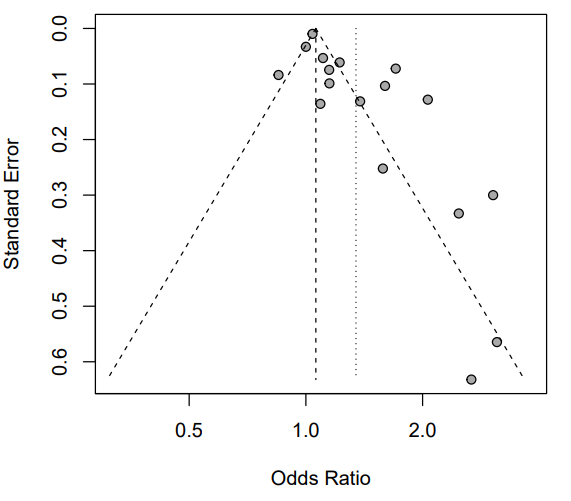 | 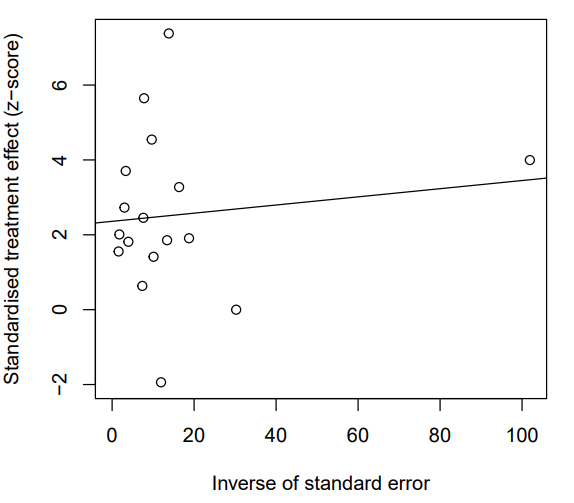 | 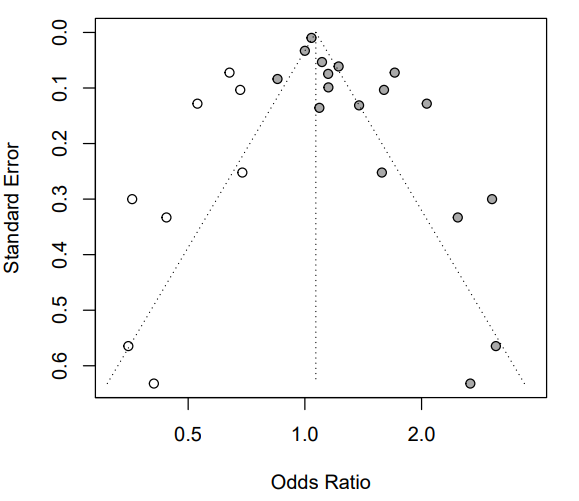 |
| **C1. Antibiotic prophylaxis (second- or third-generation cephalosporin vs First-generation)** | | |
| 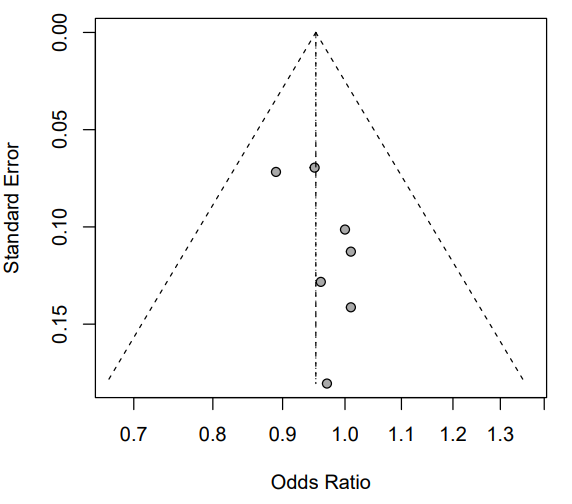 | 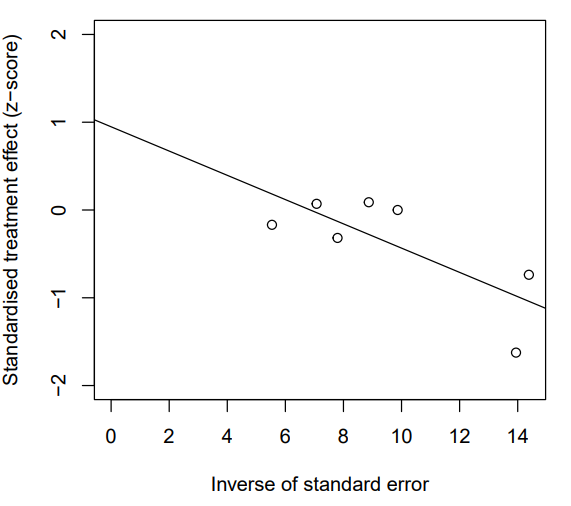 | 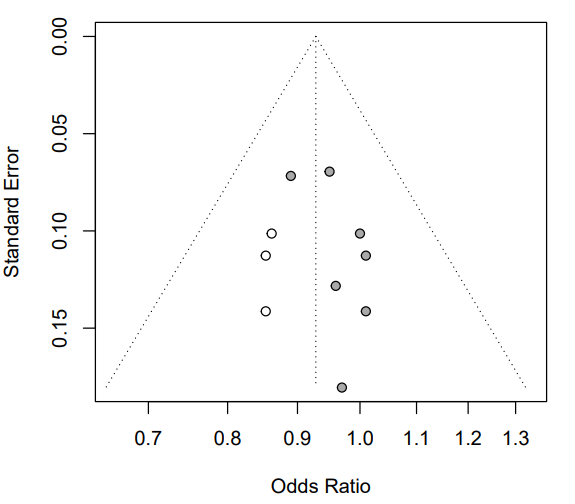 |
|  | | |
| **C2. Antibiotic prophylaxis (broad-spectrum antibiotics vs First-generation)** | | |
| 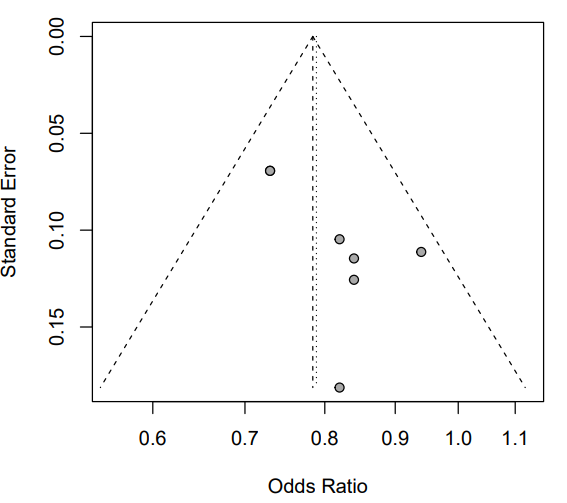 | 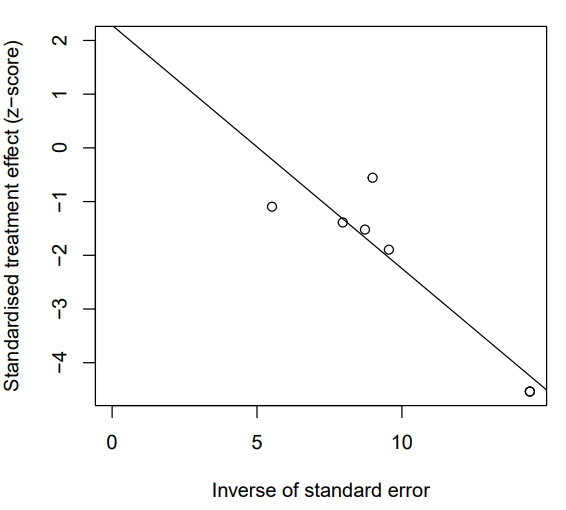 |  |
| **D. Operation time (Long vs Short)** | | |
|  |  |  |
| **E. Perioperative blood transfusion** | | |
|  |  |  |
|  | | |
| **F. Sex (Male vs Female)** | | |
|  |  |  |
| **G. Postoperative pancreatic fistula** | | |
|  |  |  |
| **H. Pancreatic texture (Soft vs Hard)** | | |
|  |  |  |
|  | | |
| **I. Preoperative albumin (Low level vs High)** | | |
|  |  |  |
| **J. Diabetes mellitus** | | |
|  |  |  |
| **K. Cardiac disease** | | |
|  |  |  |
|  | | |
| **L. Age (Older vs Young)** | | |
|  |  |  |
| **M. American Society of Anesthesiologists (ASA) classification (≥3 score vs <3 score)** | | |
|  |  |  |
| **Figure 10.** Publication bias assessment graphs of risk factors of SSIs | | |

# Results of USA

## Analysis of prevalence of SSIs

|  |
| --- |
| **Figure 11.** Forest plots of prevalence of SSIs in USA |

## Analysis of risk factors of SSIs

### Subgroup analysis

#### Group variables by study design

| **A. Preoperative biliary stenting** |
| --- |
|  |
| **Figure 12.** Forest plot of risk factors grouped by study design in USA |
| *The studies on body mass index analysis in USA are all cohort study. |

#### Group variables by type of SSIs

| **A. Preoperative biliary stenting** |
| --- |
|  |
| **B. Body mass index (High vs Low)** |
|  |
| **Figure 13.** Forest plots of risk factors grouped by type of SSIs in USA |

### Sensitivity analysis

| **Random-effect model** | **Common-effect model** |
| --- | --- |
| **A. Preoperative biliary stenting** | |
|  |  |
| **B. Body mass index (High vs Low)** |  |
|  |  |
| **Figure 14.** Sensitivity analysis of risk factors of SSIs in USA | |

### Publication bias assessment

| **Funnel plot** | **Egger’s regression test graph** | **Trim and fill graph** |
| --- | --- | --- |
| **A. Preoperative biliary stenting** | | |
|  |  |  |
| **B. Body mass index (High vs Low)** | | |
|  |  |  |
| **Figure 15.** Publication bias assessment graphs of risk factors of SSIs in USA | | |

# Results of Japan

## Analysis of prevalence of SSIs

|  |
| --- |
| **Figure 16.** Forest plots of prevalence of SSIs in Japan |

## Analysis of risk factors of SSIs

### Subgroup analysis

#### Group variables by study design

| **A. Operation time (Long vs Short)** |
| --- |
|  |
| **B. Postoperative pancreatic fistula** |
|  |
| **Figure 17.** Forest plot of risk factors grouped by study design in Japan |

#### Group variables by type of SSIs

| **A. Operation time (Long vs Short)** |
| --- |
|  |
| **B. Postoperative pancreatic fistula** |
|  |
| **Figure 18.** Forest plot of risk factors grouped by type of SSIs in Japan |

### Sensitivity analysis

| **Random-effect model** | **Common-effect model** |
| --- | --- |
| **A. Operation time (Long vs Short)** | |
|  |  |
| **B. Postoperative pancreatic fistula** |  |
|  |  |
| **Figure 19.** Sensitivity analysis of risk factors of SSIs in Japan | |

### Publication bias assessment

| **Funnel plot** | **Egger’s regression test graph** | **Trim and fill graph** |
| --- | --- | --- |
| **A. Operation time (Long vs Short)** | | |
|  |  |  |
| **B. Postoperative pancreatic fistula** | | |
|  |  |  |
| **Figure 20.** Publication bias assessment graphs of risk factors of SSIs in Japan | | |

# Other risk factors of SSIs based on insufficient evidence

| **A. Pancreatic duct (≤3mm vs 6mm)** |
| --- |
|  |
| **B. Surgical procedure (Robot-assisted vs Open)** |
|  |
| **C1. Neoadjuvant therapy (Neoadjuvant chemotherapy vs Non)** |
|  |
| **C2. Neoadjuvant therapy (Neoadjuvant radiotherapy vs Non)** |
|  |
| **D. Wound protector used** |
|  |
| **E1. Incision (Subcostal vs Midline)** |
|  |
| **E2. Incision (Unknown vs Midline)** |
|  |
| **F. Preoperative cholangitis** |
|  |
| **G1. Wound classification (Contaminated vs Clean)** |
|  |
| **G2. Wound classification (Dirty vs Clean)** |
|  |
| **Figure 21.** Forest plot of risk factors of SSIs based on insufficient evidence |
