# Supplementary material for: Prevalence of and risk factors for surgical site infections after pancreaticoduodenectomy: a systematic review and meta-analysis
Source: Ann Med Surg (Lond). 2023 Nov 7;86(1):439–55. doi: 10.1097/MS9.0000000000001455 (PMC10783382; doi:10.1097/MS9.0000000000001455)
Supplement: SUPPLEMENTARY MATERIAL [file ms9-86-439-s004.docx]

**PIRSMA Flowchart of the study selection**

| **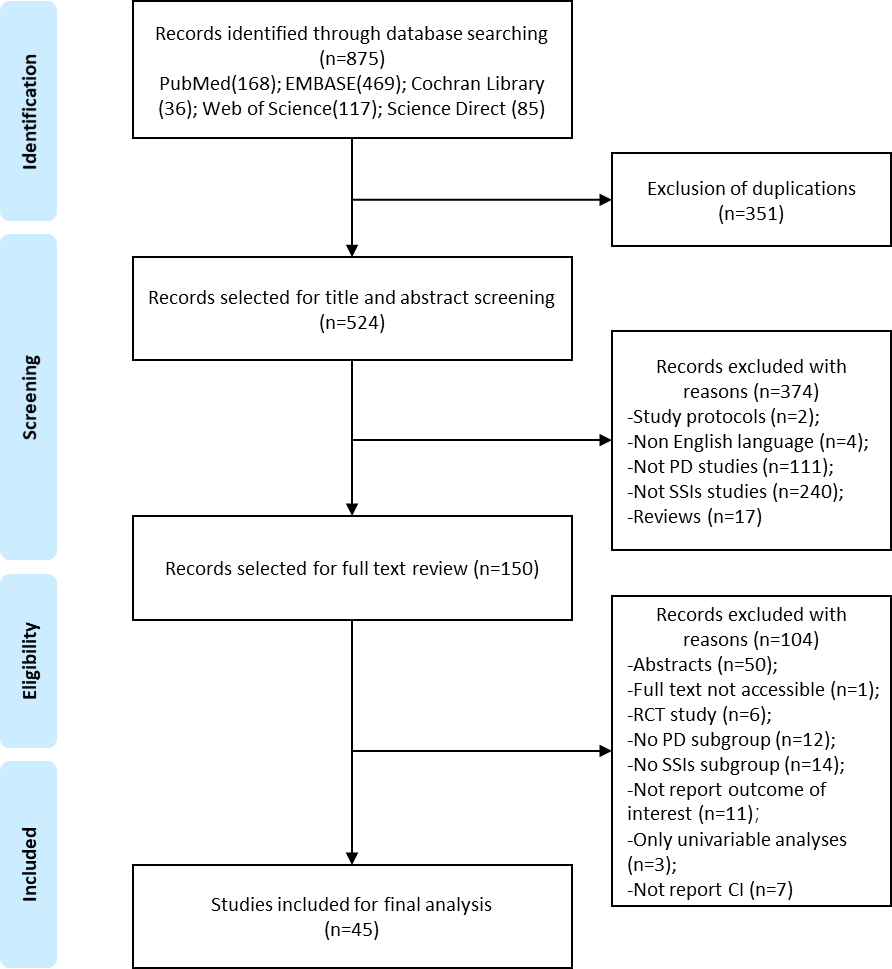** |
| --- |
| **Figure 1.** PIRSMA Flowchart of the study selection |
| **Abbreviation:** PD: Pancreaticoduodenectomy; SSIs: Surgical site infections; RCT: Randomized controlled trial; CI: Confidence interval |
